# Highly enhanced chiroptical effect from self-inclusion helical nanocrystals of tetraphenylethylene bimacrocycles†

**DOI:** 10.1039/d4sc03599b

**Published:** 2024-09-10

**Authors:** Ming Hu, Feng-Ying Ye, Wei Yu, Kang Sheng, Zhi-Rong Xu, Jin-Jin Fu, Xin Wen, Hai-Tao Feng, Minghua Liu, Yan-Song Zheng

**Affiliations:** a Key Laboratory of Material Chemistry for Energy Conversion and Storage, Ministry of Education, School of Chemistry and Chemical Engineering, Huazhong University of Science and Technology Wuhan 430074 China zyansong@hotmail.com; b AIE Research Center, Shaanxi Key Laboratory of Phytochemistry, College of Chemistry and Chemical Engineering, Baoji University of Arts and Sciences Baoji 721013 China haitaofeng907@163.com; c Beijing National Laboratory for Molecular Science (BNLMS), CAS Key Laboratory of Colloid Interface and Chemical Thermodynamics, Institute of Chemistry, Chinese Academy of Sciences Beijing 100190 China

## Abstract

The helical structure is often the key factor for forming and enhancing chiroptical properties, such as circular dichroism (CD) and circular polarized luminescence (CPL) effects. However, no matter whether helical molecules or helical aggregates, they usually display modest chiroptical signals, which limits their practical applications. Herein, chiral tetraphenylethylene (TPE) bimacrocycles prepared in almost quantitative yield show strong and repeatable CD signals up to more than 7000 mdeg, which is very rare for general organic compounds, besides emitting very strong CPL light with an absolute *g*_lum_ value up to 6.2 × 10^−2^. It is found that the superhelices formed by self-inclusion between the cavity and outward cyclohexyl ring of TPE bimacrocycles in crystal state are the key factor for highly enhanced chiroptical effect, and the self-inclusion superhelices in assemblies are confirmed by High Resolution Transmission Electron Microscopy (HR-TEM), Powder X-ray Diffraction (XRD) and Fourier Transform Infrared Spectrometry (FT-IR) data. Furthermore, the chiral TPE bimacrocycle shows great potential in chiral recognition and chiral analysis not only for chiral acids but also for chiral amines, chiral amino acids, and neutral chiral alcohol. Using self-inclusion helical nanocrystals of chiral macrocycles, this work provides a new strategy for chiroptical materials with excellent chiroptical properties.

## Abstract 2

New chiral TPE bimacrocycles exhibit helical self-inclusion in crystals, strong CD (>7000 mdeg) and CPL (absolute g_lum_ 0.062) signals, and significant potential in enantiomer analysis for diverse chiral molecules.

## Graphical abstract

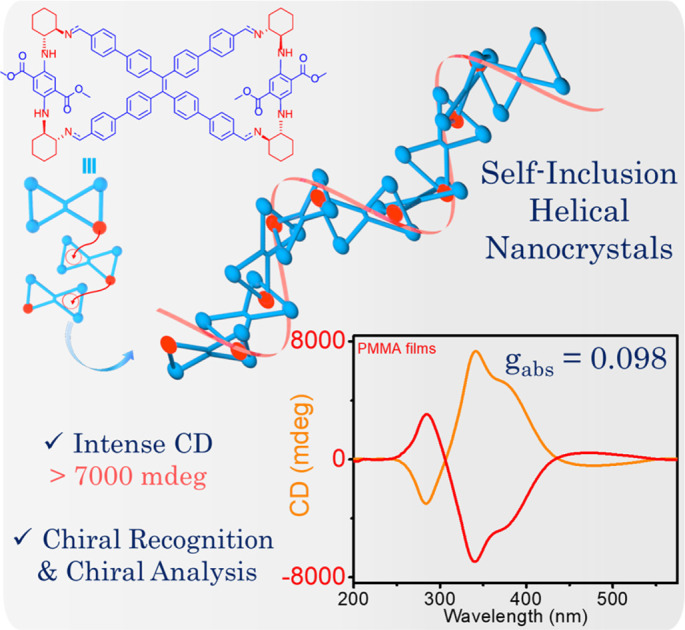

## Introduction

Chiroptical materials are attracting increasing interest due to extensive and important potential applications in light detection and ranging devices,^1,2^ 3D display,^3^ chemo/biosensing,^4,5^ information storage and processing,^6^ asymmetric optical response,^7^ and so on. However, because the dimension of most molecules is much shorter than the wavelength of light, the chiroptical response, such as circular dichroism (CD) and circular polarized luminescence (CPL), is usually very weak. Therefore, helical aggregation and helical arrangement of molecules or nanostructures is currently one of the main methods for enhancing the chiroptical effect.^8–11^ Among the helical chiroptical systems, helical assemblies of nanorods or nanoparticles of noble metals and helical liquid crystal due to plasmonic resonance and long-range ordered helical phase, respectively, can show intense CD response with the intensity of more than 1000 mdeg.^12–17^ The giant optical effect is essential in practical applications for obtaining high resolution and sensitivity of optical devices or sensors.^1–7,12–14^ However, for most helical systems, including helical molecules,^18^ helical supramolecules,^19^ helical aggregates,^20,21^ and helical polymers,^22,23^ their CD and CPL intensities are generally at about 100 mdeg. For example, the induced CD signals can be exploited in obtaining the structural information of chiral molecules and show great potential in high throughput quantitative analysis of enantiomeric purity.^24–26^ Because the induced CD signals are generally weak, chiral analysis often needs to be implemented at high concentrations, which is not beneficial for chiral drugs and products in tiny amounts.

By virtue of large size, rigidity and cavity, macrocyclic compounds are excellent candidates as a new class of chiroptical materials.^27–29^ Their chiroptical properties could not only be tuned by host–guest interactions^30,31^ but also be boosted by helical twisting^32,33^ and helical stacking,^34–36^ showing the potential in chiroptical materials. However, chiral macrocycles with excellent chiroptical properties are rare. In this paper, we report novel chiral tetraphenylethylene (TPE) bimacrocycles that could form superhelices in crystalline state by self-inclusion and exhibited intense CD signals up to more than 7000 mdeg, which is very rare for general organic compounds. Using the intense CD effect, the chiral TPE bimacrocycle showed excellent chiral recognition ability not only for chiral acids but also for chiral amines, chiral amino acids, and even neutral chiral alcohol through host–guest interactions. Meanwhile, the intense CD signals were exploited in the enantiomer excess percent of chiral amine and chiral acid compounds at low concentrations with high accuracy.

## Results and discussion

### Synthesis of TPE bimacrocycles

**Scheme 1:**
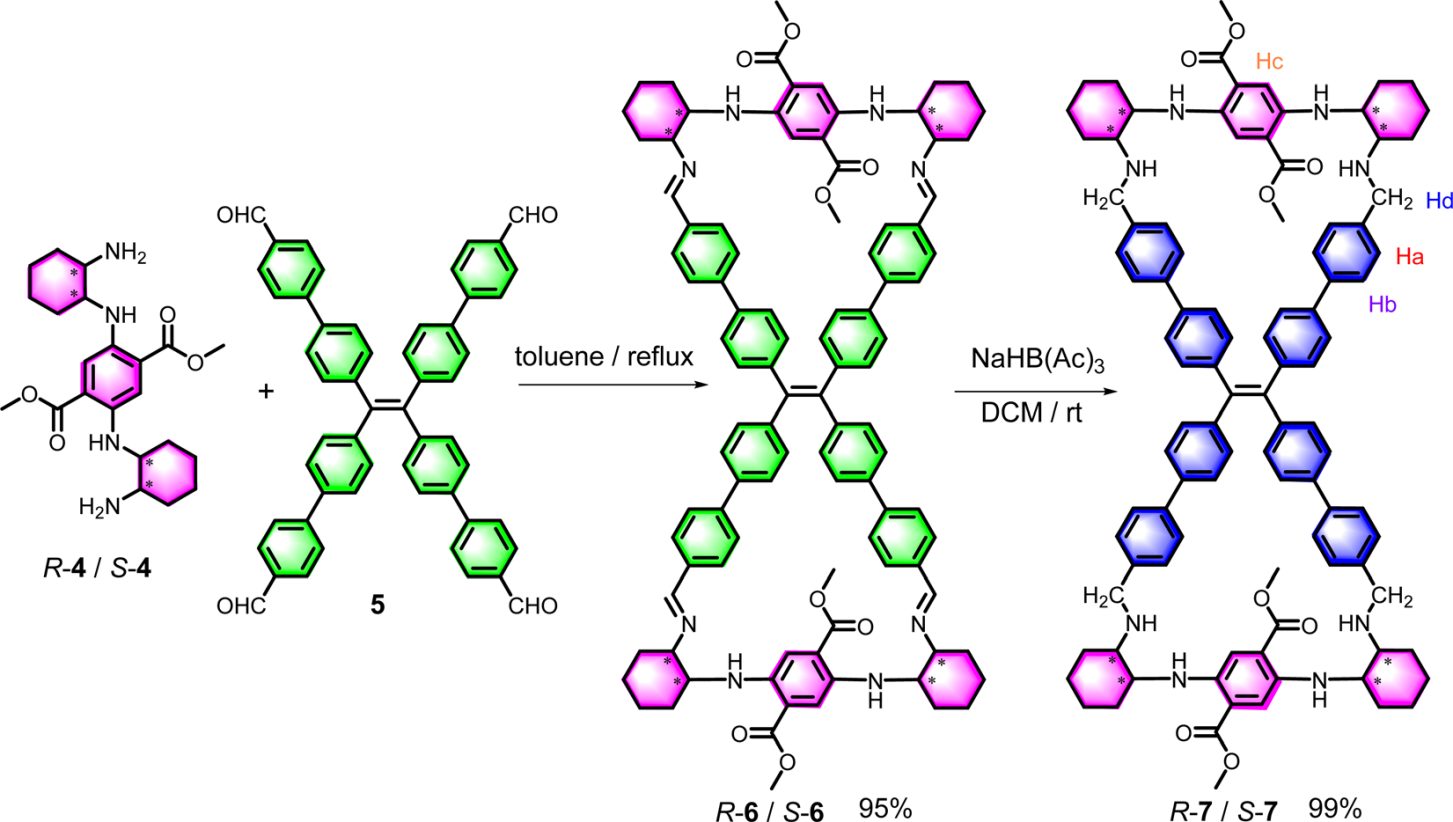
Synthetic route of the TPE bimacrocycles 6 and 7.

TPE imine bimacrocycle 6 was synthesized at a 95% yield by the condensation reaction of chiral diaminocyclohexyl-*p*-diamino-terephthalate (DATP) 4 and TPE tetrabenzaldehyde 5 (Scheme 1). After the reduction of the imine groups of 6 using sodium triacetoxyborohydride, a more stable TPE tetramine bimacrocycle 7 was obtained in the quantitative yield. The very low solubility of 6 in the reaction solvent led to its precipitation and prevented its reversible decomposition, and the use of mild agents in the reduction step prevented the destruction of the imine and ester groups. Therefore, the yield of macrocycle formation and reduction was exceptionally high. These two TPE bimacrocycles were fully characterized by ^1^H/^13^C NMR, HRMS and IR spectra (Fig. S1–S24†).

### Crystal structure

**Fig. 1:**
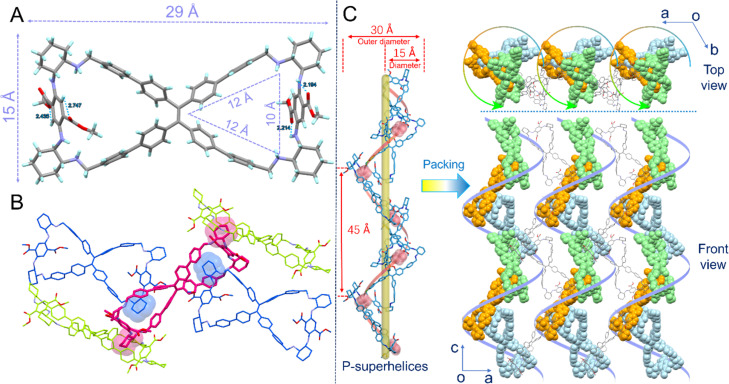
(A) Crystal structure of (*R*)-7. (B) 1 + 4 self-inclusion complex of one *R*-7 molecule with four neighboring molecules by the insertion of a cyclohexyl ring into the cavity. (C) *P*-Superhelices formed by helical self-inclusion stacking.

A single crystal of *R*-7 suitable for X-ray diffraction was obtained from a mixed solvent of methanol and 1,4-dioxane in the presence of hydrogen chloride. Its crystal structure (Fig. 1A) revealed that two cycles were formed by bridging two phenyl rings at the *cis*-position of the TPE unit. The resultant *R*-7 macrocycle had a size of 29 × 15 Å and possessed two isosceles triangle cavities with a side length of 10 × 12 × 12 Å for each one. Noticeably, the four cyclohexyl rings on the bridge chains were stretched outward away from the cavity because of the steric space limitation for forming the cavity. Due to the controlling role of the chiral cyclohexanediamine configuration, the four phenyl rings of all the TPE units were arranged in a left-hand helical (*M*-) direction, and the two DATP units possessed planar chirality with an *S*-configuration.

Very interestingly, the outward cyclohexyl rings of one *R*-7 molecule could deeply insert into the cavity of other molecules to form self-inclusion in the crystal state (Fig. 1B). Surprisingly, two cavities and four outward cyclohexyl rings of one *R*-7 molecule could be so fully exploited that one 1 + 4 self-inclusion complex *via* host–guest interactions between cyclohexyl groups and cavities was produced. As shown in Fig. 1B, the central *R*-7 molecule (red) was occupied in its two cavities by the cyclohexyl ring (blue) of the two neighboring molecules. Meanwhile, its two outward cyclohexyl rings (red) are inserted deeply into the cavity of two other neighboring molecules (green). In this host–guest interaction, the short distance between hydrogen atoms of the cyclohexyl ring and the carbon atoms of the aromatic rings was 2.878 Å, 2.803 Å, 2.757 Å and 2.736 Å from the phenyl rings of the TPE unit; 2.845 Å and 2.802 Å from phenyl rings attached to the TPE unit; and 2.798 Å, 2.800 Å and 2.850 Å from bridging phenylene groups. Numerous CH–π interactions indicated that the host–guest interaction was strong, and the resultant self-inclusion complex was stable.

Moreover, from the ob direction in the crystal cell, it was revealed that a special superhelix was formed (Fig. 1C). By the head-to-tail linkage of *R*-7 molecules through host–guest interactions between cyclohexyl groups and cavities, a right-handed (*P*-) helical superhelix was furnished. It should be noted that the helical direction of the superhelix was inverse to the *M*-helical sense of the TPE unit. The superhelix had a diameter of 15 Å that was the same as the width of the *R*-7 molecule, and an outer diameter of 30 Å and a helical pitch of 45 Å. Between two superhelices, other TPE bimacrocycle molecules can use their cyclohexyl rings at the two ends to insert into the cavities of two neighboring superhelices and connect these two superhelices together. In this way, many superhelices were aligned in parallel to form a 2D network. Meanwhile, *C*_3_ symmetry structures were observed from the top view (from the oc direction), indicating that each pitch contained three *R*-7 molecules. The self-inclusion^37^ phenomenon in macrocyclic compounds is common and easily leads to supramolecular polymers,^38–41^ but the macrocycle self-inclusion superhelix has not yet been observed to date. Overall, the macrocycle *R*-7 displayed a hierarchical chirality in its crystal state, including central chirality, planar chirality, TPE *M*-helical chirality, and long-range *P*-superhelical one.

### Photophysical properties

**Fig. 2:**
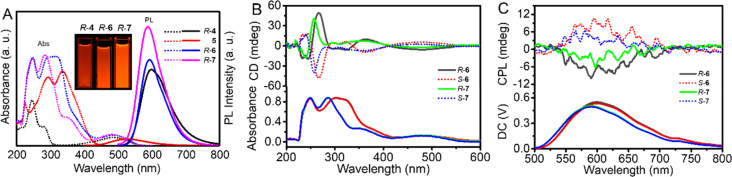
(A) Absorption (dot line) and emission spectra (solid line) of *R*-4, 5, *R*-6 and *R*-7 in DCE, inset: photographs of *R*-4, *R*-6 and *R*-7 in DCE under UV light (365 nm), [4] = [5] = [6] = [7] = 2.0 × 10^−5^ M, *λ*_ex_ = 280 nm (*R*-4) and 370 nm (5, *R*-6 and *R*-7). (B) CD and (C) CPL spectra of 6 and 7 enantiomers in CHCl_3_. [*R*-6] = [*R*-7] = 2.0 × 10^−4^ M for CD, [*R*-6] = [*R*-7] = 2.0 × 10^−3^ M for CPL, *λ*_ex_ = 350 nm.

Due to the push–pull electronic structure of the DATP unit, bridge linker 4 emitted strong red fluorescence with a fluorescent quantum yield (*Φ*) of 18.3%.^42^ Therefore, TPE bimacrocycles 6 and 7 also had strong red-color emissions at 594 and 591 nm with a *Φ* of 18.1% and 28.5%, respectively (Fig. 2A). However, no emission peak of the TPE unit was observed. For *R*-6, due to the presence of the imine group, which usually resulted in fluorescence quenching of the TPE unit,^43^ it only maintained the emission intensity of the DATP unit, such as 4. For *R*-7, because of an energy transfer from TPE to the DATP unit, it had a much stronger fluorescence than *R*-6 although the latter had larger rigidity.

As shown in Fig. 2B, the *R*-6 solution in chloroform had a negative cotton effect at 470 nm, followed by a positive one at 360 nm, and showed obvious multiple bisignate bands from 470 to 360 nm, 360 to 320 nm and 260 to 240 nm, indicating the formation of a single helical conformation of the TPE unit. As an enantiomer, *S*-6 displayed a perfect mirror-symmetric CD spectrum compared with *R*-6. At 266 nm, the CD signals of *R*-6 and *S*-6 reached their maximum, and the absorption dissymmetric factor (*g*_abs_) was ±1.2 × 10^−3^ (265 nm). For *R*-7/*S*-7, the CD signal intensity was significantly weaker than that of *R*-6, with a *g*_abs_ value of ±6.6 × 10^−4^. This was probably ascribed to the weaker rigidity of *R*-7 than of *R*-6.

Then, their CPL behaviors were investigated in chloroform first. *R*-6 and *S*-6 exhibited a CPL emission at 600 nm with a dissymmetric factor (*g*_lum_) of ±1.0 × 10^−3^, which was modestly similar to most CPL organic compounds (Fig. 2C). Accordingly, the CPL emission of *R*-7 and *S*-7 showed an emission peak at 590 nm and a smaller *g*_lum_ of ±5.1 × 10^−4^. The CPL direction was in accordance with the sense of the first CD band.

**Fig. 3:**
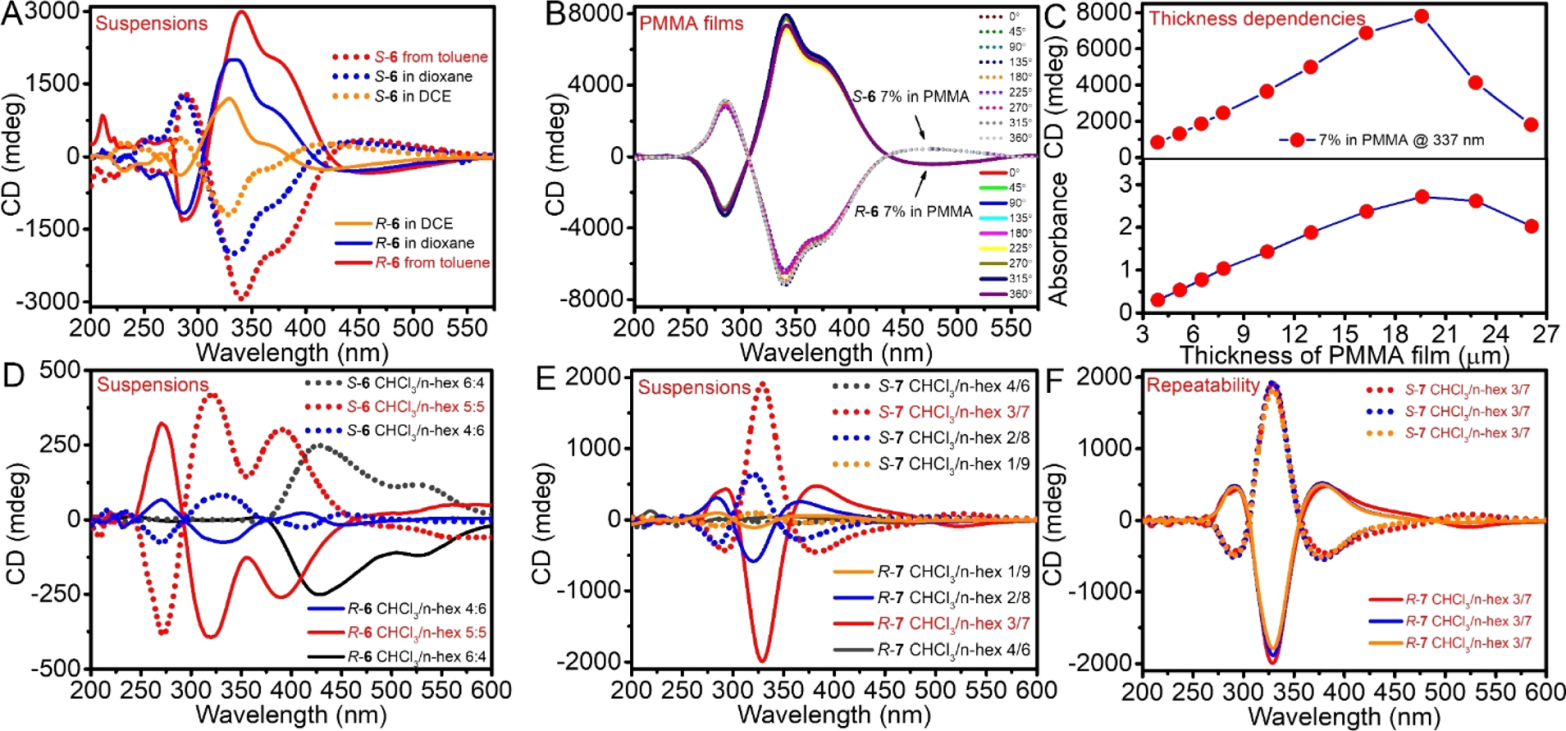
CD spectra of 6 enantiomers (A) as-prepared from toluene, suspended in 1,4-dioxane and DCE, [6] = 5.0 × 10^−4^ M. (B) PMMA films with angle change, weight%; (C) CD and absorbance intensity of *R*-6 in PMMA films with thickness change, weight%. (D and E) CD spectra of 6 and 7 enantiomers in hexane/CHCl_3_ mixed solvent, (F) the reproducibility of CD spectra of 7 enantiomers in 70% hexane/CHCl_3_, [6] = 2.0 × 10^−3^ M, and [7] = 1.0 × 10^−3^ M.

Although the TPE bimacrocycle 6 had good solubility in chloroform, it dissolved little in other common solvents, including toluene, benzene, 1,2-dichloroethane (DCE), 1,4-dioxane, and THF. Therefore, during the preparation of 6 in toluene, the product precipitated to form a suspension. To our surprise, the CD signals of the suspension as-prepared from toluene were intense and reached a value of up to 3000 mdeg, with a *g*_abs_ of ±0.039 (344 nm) (Fig. 3A). After being suspended in other solvents, such as benzene, THF, dioxane, and DCE, the CD signals could reach more than 1000 mdeg, with a *g*_abs_ of ±0.027 (351 nm), ±0.013 (334 nm), ±0.019 (340 nm), and ±0.039 (340 nm), respectively (Fig. 3A, S26, and S27†). In the drop-coating film from the benzene suspension, the CD signals also reached 2000 mdeg (Fig. S26†).

In particular, in PMMA films, 6 enantiomers displayed stronger CD intensity. For an increase in the weight fraction from 3% and 7% to 10% of 6 in PMMA, the CD signal even reached 3100, 7900 and 7500 mdeg with a *g*_abs_ of ±0.097 (335 nm), ±0.098 (340 nm) and ±0.085 (374 nm), respectively (Fig. 3B). Moreover, with measurements at different angles or by flipping, the PMMA film displayed almost the same CD intensity and invariable direction, but the intensity and direction of the linear dichroism (LD) spectrum changed with angles, indicating that CD signals did not result from the linear birefringence (LB) effect (Fig. S28–S32†). This result was very rare because general chiral organic compounds only display CD signal intensity of about 100 mdeg whether in solution or solid.^18,19,24,44,45^

To conclusively rule out the possibility of high-intensity CD signals stemming from the LD and LB effects in non-uniform samples, extensive testing was conducted on PMMA films of varying thicknesses. With the *R*-6 weight fraction maintained at 7%, both CD intensity and absorption values were observed to increase linearly with the thickness of PMMA films from 3.9 μm to 19.6 μm (Fig. 3C and S33†). The relationship between CD intensity and thickness adheres to Beer's law, thereby validating the authenticity of the CD signals observed within this thickness range.^46,47^ However, when the film thickness exceeded 19.6 μm, a notable deviation from Beer's law occurred, with both CD signals and absorption values declining. This phenomenon is likely attributed to the increased scattering caused by the large number of *R*-6 aggregates in excessively thick films. Furthermore, under optimal conditions (7% w/w weight fraction and 19.6 μm thickness), three independent parallel experiments were performed, yielding highly consistent results with maximum CD intensities consistently approaching 8000 mdeg, underscoring the excellent reproducibility of the samples (Fig. S34†).

In the suspension formed by the addition of poor solvent *n*-hexane to the solution of the TPE bimacrocycle in chloroform, the CD spectra were also measured. In 50% hexane in chloroform (volume fraction or ratio, the same below), the CD spectra of *R*-6 and *S*-6 showed stronger bisignate bands and reached more than 400 mdeg at 320 nm (Fig. 3D). Stable bimacrocyle *R*-7 also displayed increasing CD signals as the hexane fraction increased from 0% to 70%. After 70% hexane, the CD signal started to decrease but was still much larger than the solution. In particular, at the 70% hexane fraction, *R*/*S*-7 had a very strong negative Cotton effect of up to 2000 mdeg and a *g*_abs_ value of ± 1.5 × 10^−2^ at 330 nm, which was 29-fold larger than that of chloroform solution (Fig. 3E). At different hexane fractions, the CD spectra showed different shapes and wavelength shifts, but the direction of the first CD band was the same. Upon thorough examination, the exceptional high-intensity CD signals demonstrated remarkable reproducibility, consistently yielding the strongest CD signal at a 70% *n*-hexane fraction across two additional sets of *R*/*S*-7 self-assembled samples tested at 60%, 70%, and 80% *n*-hexane content, respectively (Fig. S45†). Notably, the CD spectra at the 70% hexane fraction from these replicated experiments were virtually superimposed (Fig. 3F), further corroborating their high degree of consistency.

**Fig. 4:**
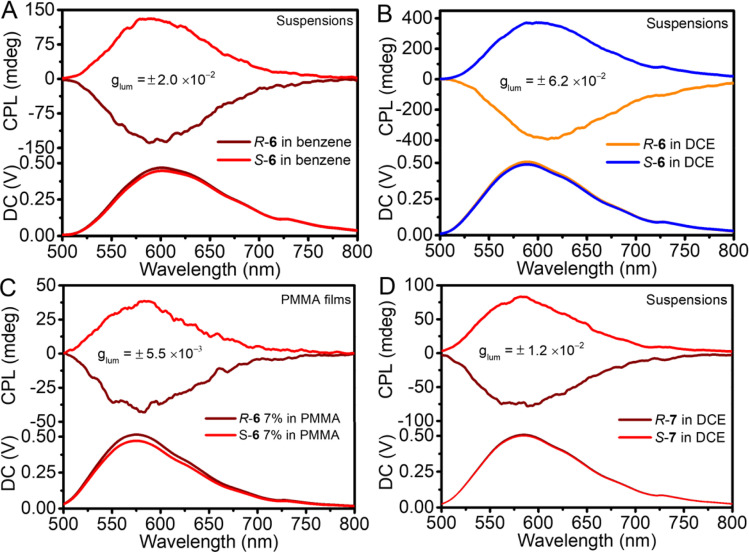
CPL spectra of (A) *R*-6 and *S*-6 in benzene, 5.0 × 10^−3^ M; (B) *R*-6 and *S*-6 in DCE, 5.0 × 10^−3^ M; (C) *R*-6 and *S*-6 in PMMA film, weight%; and (D) *R*-7 and *S*-7 in DCE, 2.0 × 10^−3^ M. *λ*_ex_ = 350 nm.

Moreover, the suspension and films of both 6 and 7 enantiomers showed enhanced CPL emission. In the suspension of 6 enantiomers in benzene, the CPL *g*_lum_ value reached ±2.0 × 10^−2^, while *R*-6/*S*-6 in DCE was able to emit a stronger CPL signal at 590 nm with a large *g*_lum_ of ±6.2 × 10^−2^, which was amplified by about 62 times over the CHCl_3_ solution (Fig. 4A and B). In PMMA and benzene suspension films, the *g*_lum_ value of 6 enantiomers was ± 5.5 × 10^−3^ and ± 1.1 × 10^−2^ (Fig. 4C and S37C†), respectively. Similarly, the CPL signals of *R*-7/*S*-7 in the DCE reached a 23.5-fold enlargement with a *g*_lum_ of ±1.2 × 10^−2^ (Fig. 4D).

**Fig. 5:**
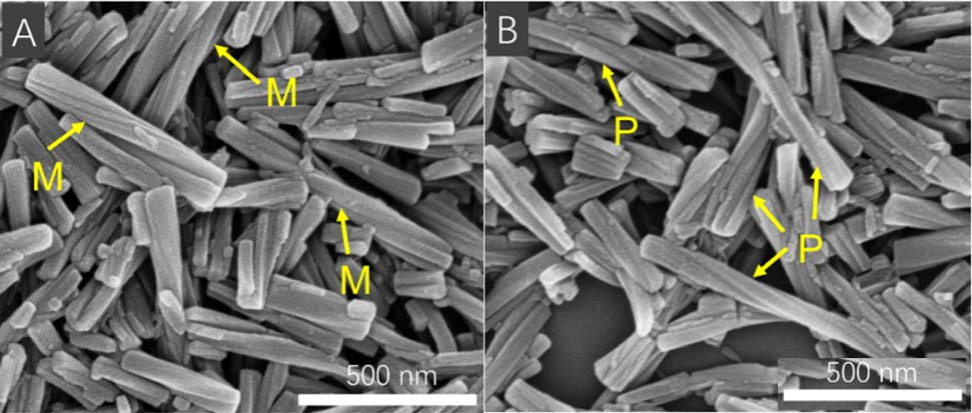
SEM images of the suspension of *R*-6 (A) and *S*-6 (B) as-prepared from toluene. [6] = 5.0 × 10^−4^ M.

The suspensions of 6 enantiomers as-prepared from toluene reaction solution were unveiled to be nanorods with a length of 20–1000 nm, with a uniform diameter of about 25 nm. Interestingly, two or more nanorods can be twined into wider helical nanorods. Noticeably, *R*-6 gave *M*-helical nanorods, while *S*-6 led to *P*-helical ones (Fig. 5). The helical direction of the nanorods was the same as that of the TPE unit but was contrary to that of the superhelix in the crystal state. In other solvents, such as benzene and DCE, the suspensions of 6 enantiomers had the same nano structure and morphology (Fig. S40 and S41†), indicating that the as-prepared nanorods could stably exist in other poor solvents and display similar intense CD signals.

**Fig. 6:**
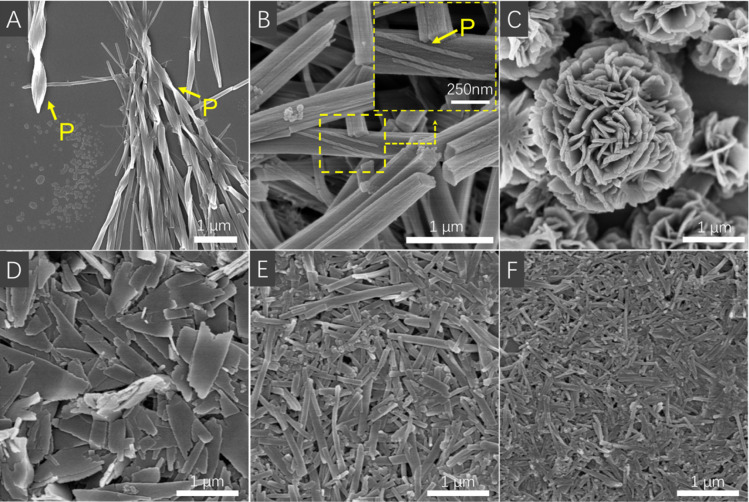
SEM images of *R*-6 in (A) 50% *n*-hexane/CHCl_3_, (B) 60% *n*-hexane/CHCl_3_; and *R*-7 in (C) 60% *n*-hexane/CHCl_3_, (D) 70% *n*-hexane/CHCl_3_, (E) 80% *n*-hexane/CHCl_3_, and (F) 90% *n*-hexane/CHCl_3_. [6] = 2.0 × 10^−3^ M and [7] = 1.0 × 10^−3^ M.

In contrast to the suspension of an as-prepared solid, the suspension formed by the addition of hexane into chloroform provided a great variety of nano structures and morphologies. When the hexane fraction in chloroform was changed from 40% and 50% to 60%, the self-assembly morphology of *R*-6/*S*-6, changed from the nanorods and twisted nanofibers to helical nanofibers. The twisted and helical directions were *P*-helical for *R*-6 and *M*-helical for *S*-6 (Fig. 6A, B and S42†), which were contrary to those of helical nanorods from the as-prepared solid but the same as those of the superhelix in the crystal state. At 60% hexane/CHCl_3_ suspension, large non-helical nanorods with a diameter of 500–1000 nm were composed of smaller helical fibers of about 40 nm diameter. Noticeably, the helical pitch of the helical nanofibers in 50% and 60% hexane/CHCl_3_ suspensions had a large difference and was significantly decreased from 1100 nm to 210 nm with an increase in the hexane fraction.

For *R*-7/*S*-7, when the hexane fraction was gradually changed from 40%, 60%, and 70% to 90%, the self-assembly morphology changed from nanoflowers, nanosheets, and nanorods, to nanorods. In sharp contrast to *R*-6/*S*-6, no surface helical morphology was found. In particular, at the 70% hexane fraction, there was an intense CD signal of up to 2000 mdeg, but only nanosheet aggregates (500–1500 nm long and 100–600 nm wide) were observed (Fig. 6C–F). Similar to the CD spectra, under the condition of a 70% hexane fraction, the assembly morphology of *R*-7/*S*-7 also exhibits excellent reproducibility, with nanosheets of similar size and shape repeatedly appearing (Fig. S46†).

**Fig. 7:**
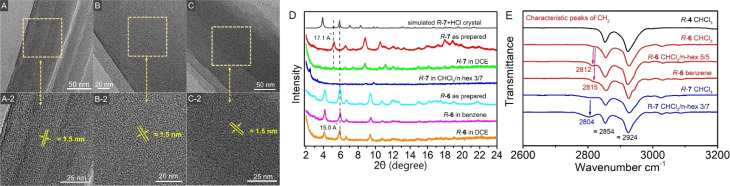
HR-TEM pictures of (A) *R*-6 in benzene (B), *R*-7 in DCE, (C) *R*-7 in 70% hexane/CHCl_3_, PXRD pattern (D) of simulated *R*-7 + HCl crystal, as prepared powders and assemblies of *R*-6 and *R*-7 under different conditions. FT-IR spectra (E) of drop-cast films of *R*-4, *R*-6 and *R*-7 from different solvents.

The fact that the aggregates did not have a surface helical pattern but showed intense CD signals motivated us to investigate the inner structure of the aggregates. It was revealed by HR-TEM images that the nanorods from the suspension of *R*-6 in benzene (Fig. 7A) and *R*-7 in DCE (Fig. 7B) showed orderly arranged crystal lattice stripes, which were about 1.5 nm wide. Similar lattice stripes were also observed in nanosheets from the suspension of *R*-7 in 70% hexane/CHCl_3_. The strip width was about 1.5 nm (Fig. 7C). This ordered strip structure in HR-TEM images hinted that the nanorods and nanosheets were crystal solid instead of amorphous ones.^11^

Similar to the simulated data from a single-crystal structure of *R*-7, the powder X-ray diffraction (PXRD) pattern of the nanorods from the suspension of *R*-6 and *R*-7 in DCE exhibited a series of diffraction peaks (Fig. 7D). Moreover, *R*-6 had a strong diffraction peak at 15.0 Å, which corresponded to the lattice fringes in the HR-TEM images. The PXRD pattern of the as-prepared product in toluene, suspension in benzene and suspension in DCE was almost the same, confirming that the as-prepared nanorods were stable when they were suspended in other poor solvents. Similarly, the nanorods of *R*-7 from the DCE also possessed a distinct diffraction peak corresponding to a distance of 17.1 Å, close to the width of the lattice fringes in the TEM image. In addition, the nanosheets from the suspension of *R*-7 in 70% hexane/CHCl_3_ showed diffraction peaks, confirming their crystalline structure observed by the TEM image.

Because the self-inclusion process occurred only in the aggregated state, it was difficult to prove the existence of the self-inclusion superhelices through ^1^H NMR data (Fig. S52†). However, the FT-IR spectroscopy of drop-cast films of *R*-6 and *R*-7 provided definitive proof of self-inclusion occurring during self-assembly (Fig. 7E). Solid *R*-4 showed two distinct peaks at 2854 cm^−1^ and 2924 cm^−1^ attributed to C–H stretching in saturated hydrocarbons,^48^ suggesting minimal intermolecular interactions on its cyclohexyl groups. Notably, *R*-6 and *R*-7 do not self-assemble in chloroform (Fig. S54†), resulting in weak CD signals and similar infrared spectra with only two peaks. In contrast, the self-assembly of *R*-6 in 50% hexane/CHCl_3_ revealed a new absorption peak at 2812 cm^−1^, with a slight shift to 2815 cm^−1^ in the benzene-derived solids. Similarly, *R*-7 self-assembled in 70% hexane/CHCl_3_ exhibits a prominent broad absorption band centered at 2804 cm^−1^. The emergence of red-shifted peaks suggests that self-inclusion suppresses C–H vibrations in parts of the cyclohexyl groups of *R*-6 and *R*-7.^49^

**Fig. 8:**
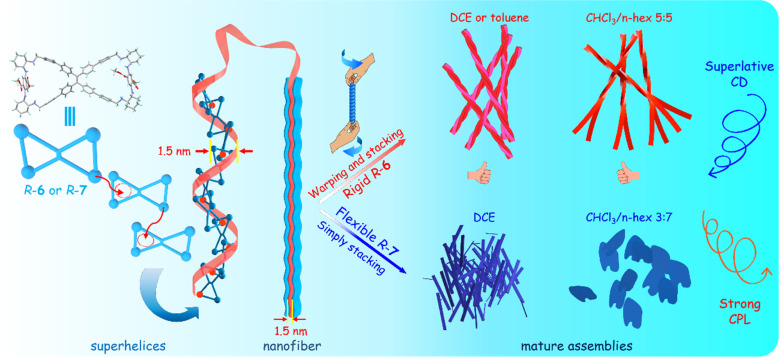
Illustration of stepwise self-assembly of *R*-6 and *R*-7 from molecular self-inclusion superhelices to nano-scale helical aggregates.

Therefore, the exceptional chiral amplification and superior chiroptical effect stemmed from the combined effects of molecular confinement, perfect helicity, and parallel stacking in the self-inclusion supramolecular structure (Fig. 8). For *R*-TPE bimacrocycles, they had a *P*-superhelical structure, which led to negative first CD band and negative CPL signals, while *S*-TPE bimacrocycles produced an *M*-superhelical structure, which aroused positive first CD band and positive CPL signals. We had previously known that the *M*-helical TPE unit produced positive CD and CPL signals, while the *P*-TPE unit led to negative ones.^50,51^ However, in this research, the *M*-TPE unit produced inverse negative chiroptical signals. It was also noted that the helical nano-aggregates of 6 had an inverse helical direction between as-prepared solids and solids from hexane–chloroform mixed solvent, but they showed the same sense of chiroptical signals. More crucially, the nanosheets from the suspension of 7 in 70% hexane/chloroform had no helical structure on the surface, but they also exhibited intense chiroptical signals. Therefore, intense chiroptical signals should contribute to the superhelical structure in the crystal state by helical self-inclusion stacking of the TPE bimacrocycles.

Both the *M*-helical structure on the surface of the nano-aggregates and the helical structure of TPE units played no key role in the direction and high intensity of the intense chiroptical signals except for probably bringing various shapes and wavelength changes of CD spectra. The reason should be that the CD signal of the large helical self-assembly or helical aggregation was so small compared with the intense CD signal of the self-inclusion superhelices and was completely overpowered by the latter. This result is very contrary to many reference results in which the helical chirality of nano-aggregates determines the direction and intensity of chiroptical signals.^8–11,19–21,44,45^

### Chiral recognition and chiral analysis

Given that bimacrocycle 7 showed intense CD signals and was stable (Fig. S57†), it could be used as a chiral reagent for chiral recognition and analysis in a diluted solution. After the suspension of *R*-7 in 80% hexane/CHCl_3_ was diluted with this mixed solvent from 10^−3^ M to 10^−5^ M concentration level, it still had an intensity of about 100 mdeg. In contrast, for most chiral molecules, their CD signal completely disappeared at 10^−5^ M concentration level.^18–25,44,45^ For the tested chiral guest molecules 8–16, their CD signals disappeared when the concentration was diluted to 1.0 × 10^−4^ M – 5.0 × 10^−5^ M (Fig. S58†).

**Fig. 9:**
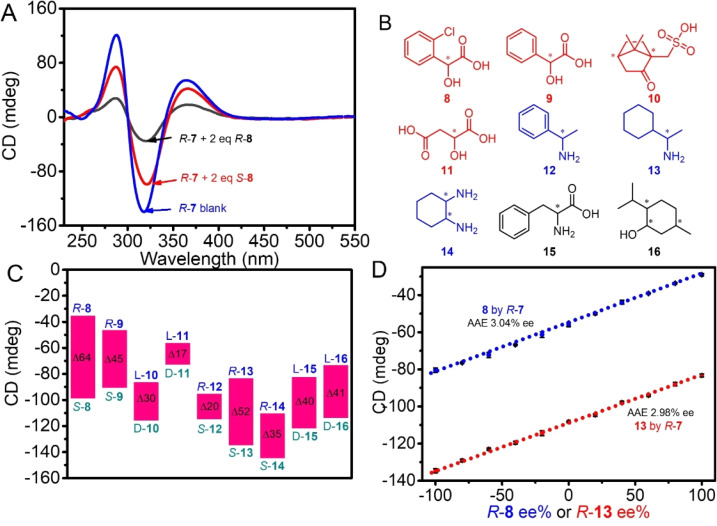
(A) Change in CD spectra of *R*-7 with two enantiomers of 8 in 80% hexane/CHCl_3_, [*R*-7] = 1/2[8] = 5.0 × 10^−5^ M. (B) Structure of chiral reagents 8–16. (C) CD intensity difference of *R*-7 aroused by two enantiomers of testing chiral reagents at intensity max wavelength in 80% hexane/CHCl_3_. (D) Change in max CD intensity of *R*-7 with ee% of *R*-8 ([*R*-7] = 1/2[8] = 4.0 × 10^−5^ M) and *R*-13 ([*R*-7] = 1/2[13] = 5.0 × 10^−5^ M).

With two enantiomers of 2-chloromandelic acid 8 for the test, the CD signal (absolute value) of *R*-7 weakened to 35 mdeg from 139 mdeg by the addition of 2 eq *R*-8, while it was only reduced to 99 mdeg by *S*-8, displaying a large difference of 64 mdeg between the two enantiomers (Fig. 9A). For mandelic acid 9, camphor sulfonic acid 10 and malic acid 11, *R*-7 also displayed obvious differences in CD signal intensity between their enantiomers, which were 45 mdeg, 30 mdeg and 17 mdeg (Fig. 9C and S59†).

More interestingly, although 7 was a secondary amine and was basic, it had a different response to the two enantiomers of chiral amine alpha-methylbenzylamine 12, 1-cyclohexylethylamine 13 and 1,2-diaminocyclohexane 14, chiral amino acid phenylalanine 15, and even neutral compound menthol 16. The resultant CD intensity differences between the two enantiomers of 12, 13, 14, 15, and 16 were 20 mdeg, 52 mdeg, 35 mdeg, 40 mdeg and 41 mdeg, respectively (Fig. 9C and S59†).

A significant change in the CD intensity of *R*-7 with chiral guests was exploited to determine the enantiomeric purity of the chiral guests. By keeping the molar ratio of *R*-7 to the mixture of *R*-8 and *S*-8 at a 1 : 2 molar ratio, it was found that the CD intensities of *R*-7 linearly decreased with ee% of *R*-8 ranging from −100% to 100% (Fig. 9D and S60†). The relationship between CD intensity and ee% was a straight line, which could be utilized as a calibration curve for determining the enantiomer purity of 8 with unknown ee%. The average absolute error (AAE) between measured ee values and actual ones was 3.04% ee, which is comparable with the CD chiral analysis in concentrated solution.^23–25^ As one more demonstration, the enantiomer purity of the chiral amine 13 could also be analyzed by *R*-7 with an AAE of 2.98% (Fig. 9D and S61†).

**Fig. 10:**
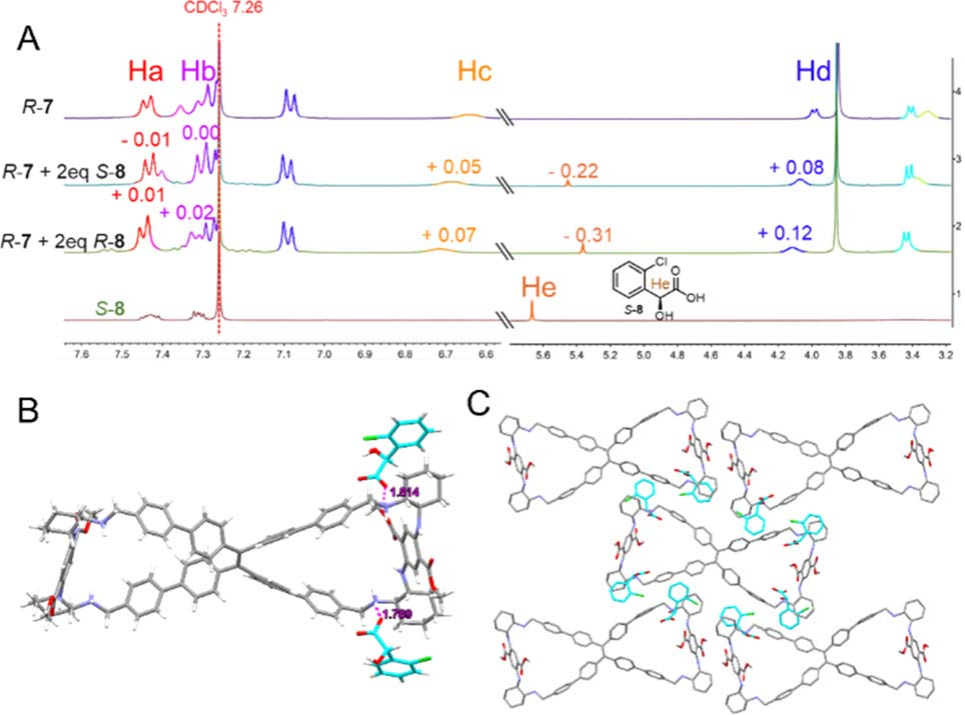
(A) ^1^H NMR spectra of *R*-7, *S*-8, *R*-7 + 2 eq *S*-8 and *R*-7 + 2 eq *R*-8. [*R*-7] = 1/2[8] = 2.0 × 10^−3^ M. (B) Crystal structure of *R*-7 + *S*-8 complex. (C) Packing mode of molecules of *R*-7 + *S*-8 complex.

To elucidate the principle of chiral recognition in depth, the ^1^H NMR spectral analysis was conducted on a mixture comprising *R*-7 and enantiomers of chiral acid 8. As illustrated in Fig. 10A, notable downfield shifts (+0.07 ppm and +0.12 ppm, respectively) in the chemical shifts of Hc and Hd, hydrogen atoms proximal to the amino group on *R*-7, were observed in the presence of *R*-8, while these shifts were attenuated (+0.05 ppm and +0.08 ppm, respectively) when mixed with *S*-8. Conversely, a more pronounced upfield shift (−0.31 ppm) of the He hydrogen atom on *R*-8 was evident in the *R*-7 + *R*-8 complex compared to the *R*-7 + *S*-8 complex (−0.22 ppm), highlighting distinct intermolecular interaction profiles. Subsequently, the binding stoichiometry between *R*-7 and both enantiomers (*R*-8 and *S*-8) was determined to be 1 : 2 using the Job plot method (Fig. S62†). Then, the respective binding constants (*K*) were calculated by applying the Benesi–Hildebrand equation for 1 : 2 association,^52^ yielding values of 2.3 × 10^8^ M^−1^ for *R*-7 + *R*-8 (*K*_*R*-__7__+*R*-__8_) and 1.1 × 10^8^ M^−1^ for *R*-7 + *S*-8 (*K*_*R*-__7__+*S*-__8_) (Fig. S63†), thus emphasizing the preferential affinity of *R*-7 towards *R*-8 over *S*-8 by *a* factor of 2.1 (*K*_*R*-__7__+*R*-__8_/*K*_*R*-__7__+*S*-__8_).

Furthermore, the fortunate acquisition of the single-crystal structures of the complexes formed between *R*-7 and *S*-8 provided invaluable insights. The crystal structures revealed that a single *R*-7 molecule bound with two *S*-8 molecules was facilitated by hydrogen bonding interactions between the carboxylate anions of *S*-8 and the protonated benzylamine groups of *R*-7 (Fig. 10B). In the packing arrangement, *S*-8 molecules encircled *R*-7, which hinders intermolecular interactions among *R*-7 molecules, consequently precluding the formation of self-inclusion superhelices within the unit cell (Fig. 10C). Therefore, it was reasonable to speculate that when a suspension of *R*-7 in 80% hexane/CHCl_3_ was exposed to the enantiomers of 8, the latter partially disrupted the self-assembly of *R*-7, resulting in attenuation of the CD signal. Notably, the stronger interaction between *R*-7 and *R*-8 compared to *R*-7 and *S*-8 leads to a more pronounced reduction in the CD signal, suggesting a higher degree of disruption in the self-assembly.

In contrast, lacking powerful acid–base interactions, *R*-7 and enantiomers of chiral amine 13 still exhibited observable ^1^H NMR chemical shift changes in their mixture, with both protons undergoing upfield shifts, suggesting potential host–guest interactions within the TPE bimacrocycle cavity. Notably, *R*-13 exhibited a more significant upfield shift than *S*-13 (Fig. S64†). The binding stoichiometry between *R*-7 and both *R*-13 and *S*-13 was determined to be 1 : 2 (Fig. S65†), with respective binding constants of 8.2 × 10^7^ M^−1^ (*K*_*R*-__7__+*R*-__13_) and 4.6 × 10^7^ M^−1^ (*K*_*R*-__7__+*S*-__13_) (Fig. S66†). The *R*-7 + *R*-13 complex displayed greater variations in H-shift and possessed a superior binding constant, ultimately leading to a more fragmented assembly (Fig. S67†). This phenomenon was consistent with the more pronounced alteration in CD intensity observed upon the addition of *R*-13 compared to *S*-13.

## Conclusions

In conclusion, novel chiral TPE bimacrocycles were synthesized in almost a quantitative yield. The TPE bimacrocycles could self-assemble into a superhelical structure through host–guest interactions between their cavity and outward cyclohexyl ring. The HR-TEM and P-XRD data revealed that the assemblies with high-intensity CD signals exhibited distinct crystalline characteristics, while the FT-IR spectra offered direct evidence of the presence of self-inclusion structures within these assemblies. For the first time, it was found that the self-inclusion superhelices in crystalline state could generate intense and repeatable CD signals of up to more than 2000 mdeg and can even reach more than 7000 mdeg in PMMA film, in addition to emitting very strong CPL signals with an absolute *g*_lum_ value of up to 6.2 × 10^−2^. Furthermore, although the chiral TPE bimacrocycle was an amine compound, it could recognize the two enantiomers of not only chiral acids but also chiral amines, chiral amino acids, and even neutral chiral alcohol through host–guest interactions besides acid–base interactions. Very exceptionally, the intense CD signals could be exploited in the analysis of the enantiomeric purity of chiral amine and acid compounds at low concentrations with high accuracy, providing the first example of chiral analysis by CD intensity change in diluted solution. Using helical self-inclusion nanocrystals of macrocycles to obtain an intense chiroptical effect, this work opens a new way for chiroptical materials with excellent properties.

## Author contributions

Y.-S. Z. and M. H. designed the experiments. M. H. conducted the synthesis and characterization of all compounds and wrote the paper. F.-Y. Y. conducted partial synthesis and data analysis. W. Y., K. S., Z.-R. X, J.-J. F, and X. W. provided necessary assistance during the experimental process. Y.-S. Z., H.-T. F. and M. L. provided scientific advice and revised the draft.

## Conflicts of interest

There are no conflicts to declare.

## Supplementary Material

SC-015-D4SC03599B-s001

SC-015-D4SC03599B-s002

## Data Availability

Compound characterization data and experimental procedures are available in the ESI.† Crystallographic data of *R*-7 hydrochloride and *R*-7–*S*-8 complex have been deposited at the CCDC under 2317906 and 2351711 and can be obtained from https://doi.org/10.1039/d4sc03599b.
