# Supplementary material for: Highly enhanced chiroptical effect from self-inclusion helical nanocrystals of tetraphenylethylene bimacrocycles
Source: Chem Sci. 2024 Sep 10;15(40):16627–36. doi: 10.1039/d4sc03599b (PMC11414835; doi:10.1039/d4sc03599b)
Supplement: SC-015-D4SC03599B-s001 [file SC-015-D4SC03599B-s001.pdf]

Supplementary Information (SI) for Chemical Science.
This journal is © The Royal Society of Chemistry 2024

Supporting Information

Highly Enhanced Chiroptical Effect from Self-Inclusion Helical Nanocrystals of Tetraphenylethylene Bimacrocycles

Ming Hu,† Feng-Ying Ye,† Wei Yu,† Kang Sheng,† Zhi-Rong Xu,† Jin-Jin Fu,† Xin Wen,§ Hai-Tao Feng,*,‡ Minghua Liu,§ and Yan-Song Zheng*,†

† Key Laboratory of Material Chemistry for Energy Conversion and Storage, Ministry of Education, School of Chemistry and Chemical Engineering, Huazhong University of Science and Technology, Wuhan 430074, China. Email: zyansong@hotmail.com.

‡ AIE Research Center, Shaanxi Key Laboratory of Phytochemistry, College of Chemistry and Chemical Engineering, Baoji University of Arts and Sciences, Baoji 721013, China. Email: haitaofeng907@163.com.

§ Beijing National Laboratory for Molecular Science (BNLMS), CAS Key Laboratory of Colloid Interface and Chemical Thermodynamics, Institute of Chemistry, Chinese Academy of Sciences, Beijing 100190, China

Materials and Methods

Materials: All reagents and solvents were chemical pure (CP) grade or analytical reagent (AR) grade and were bought from China National Pharmaceutical Group Corporation, Aladdin (Shanghai) Bio-Chem Technology Co Ltd, and Meryer (Shanghai) Chemical Technology Co Ltd et al. These reagents and solvents were used as received unless otherwise indicated.

Measurements: ^{1}H NMR and ^{13}C NMR spectra were measured on a Bruker AV 400 spectrometer at 298 K in deuterated reagents. Infrared spectra were recorded on Bruker EQUINAX55 spectrometer. Mass spectrum was measured on an Ion Spec 4.7 Tesla FTMS instrument. Absorption spectra were recorded on a Hewlett Packard 8453 UV–Vis spectrophotometer. Fluorescent spectra were collected on a Shimadzu RF-5301 fluorophotometer at 298 K. The single crystal data was collected on Rigaku Saturn diffractometer with CCD area detector. The fluorescence quantum yield was measured by Edinburgh F55500w. Circular dichroism (CD) spectra were recorded on JASCO J-810, JASCO J-1500, J-1700 or Chirascan VX spectrometers. Circular polarized luminescence (CPL) spectra were measured on a JASCO CPL200 spectrometer. scanning electron microscope (SEM) images were recorded on a GeminiSEM300 electron microscopy. Transmission Electron Microscope (TEM) images were recorded on a FEI Technai G2 20 electron microscopy at 200 kV.

Single crystal growing: The single crystals of hydrochlorides of *R*-**7** were obtained through injecting HCl gas to the methanol suspension of *R*-**7** until reaching clear solution, and then 1,4-dioxane is added as the poor-solvent. The crystal is cultured by slow evaporation of the mixed solvent.

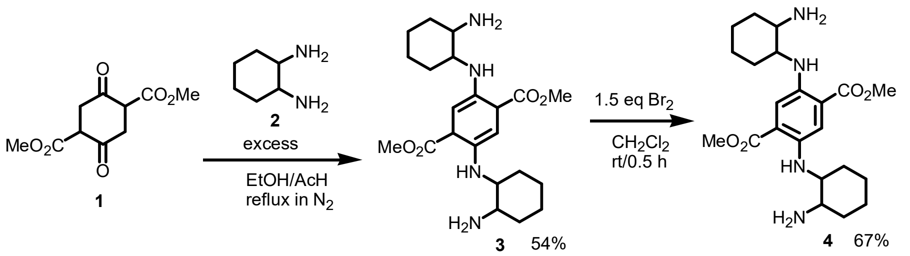

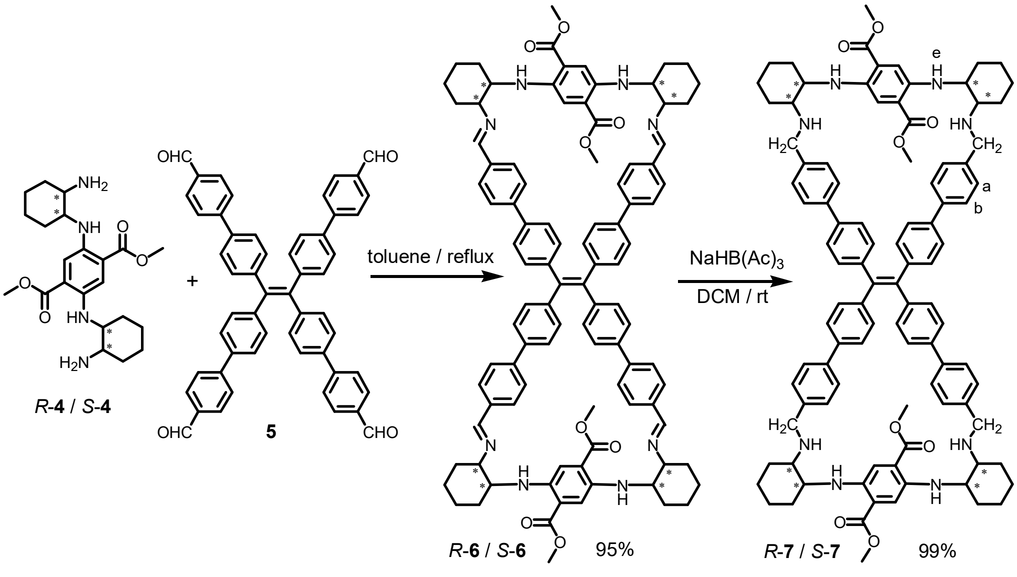

Synthesis of *R*-3 or *S*-3

To a reaction glass bottle was added *R*-**2** or *S*-**2** (25 g, 219 mmol) and 200 mL ethanol and the solution was bubbled by nitrogen for 20 minutes to remove oxygen. Then **1** (5 g, 21.9 mmol) and 10 mL acetic acid were added in order. The reaction bottle was sealed and stirred at 80 °C for 24 h. After the reaction was finished, the solution was cooled to room temperature and frozen for 4 h, and the resultant sediment was collected by filtering and rinsed with methanol to get white solid *R*-**3** or *S*-**3** (4.87 g, 8.57 mmol, 52.9%) without additional purification steps.

Synthesis of *R*-4

To *R*-**3** (4.50 g, 11.9 mmol) in 200 ml dried dichloromethane was slowly dropped liquid bromine (2.85 g, 17.8 mmol) in 10 ml at room temperature. After the solution was stirring for 30 minutes, saturated Na_2CO_3 solution and excessive dichloromethane were added. Under vigorously stirring, the reaction mixture was reverted to a bright red color. The solution was dried by Na_2SO_4 and then purified by column chromatography on silica gel with dichloromethane/methanol/triethylamine (500:40:0.5) to give compound *R*-**4** as a red solid (3.00 g, 7.2 mmol 66.7%). $[\alpha]^{25}_D = -445°$ ($CHCl_3$, c = 2 mg/mL). Mp = 176-178 °C. ^1H NMR (400 MHz, $CDCl_3$) δ 7.42 (s, 2H), 6.71 (d, J = 8 Hz, 2H), 3.89 (s, 6H), 3.07 (d, J = 8 Hz, 2H), 2.64 (dd, J = 8, 4.0 Hz, 2H), 2.11 – 1.94 (m, 4H), 1.78 – 1.67 (m, 4H), 1.62 (s, 4H), 1.33 (d, J = 8 Hz, 6H), 1.22 – 1.04 (m, 2H). ^{13}C NMR (101 MHz, $CDCl_3$) δ 168.55, 141.24, 117.33, 115.30, 59.91, 55.89, 51.94, 34.75, 32.31, 25.28, 24.86. IR (KBr) ν 3450, 3358, 3295, 3022, 2991, 2922, 2854, 1687, 1535, 1436, 1419, 1223, 1191, 1107, 875, 788. ESI^+ HRMS m/z calcd for $C_{22}H_{35}N_4O_4^+$ 419.2658, $[M + H]^+$, found 419.2675 $[M + H]^+$

Synthesis of *S*-4

The synthetic procedure was the same as *R*-**4**. $[\alpha]^{25}_{D}$ = + 458° ($CHCl_3$, c = 2 mg/mL). Mp = 176-178 °C. ^{1}H NMR (400 MHz, $CDCl_3$) δ 7.41 (s, 2H), 6.71 (d, J = 8 Hz, 2H), 3.88 (s, 6H), 3.07 (s, 2H), 2.65 (d, J = 4 Hz, 2H), 2.13 – 1.93 (m, 4H), 1.72 (s, 4H), 1.61 (s, 4H), 1.31 (d, J =8 Hz, 6H), 1.22 – 1.04 (m, 2H). ^{13}C NMR (101 MHz, $CDCl_3$) δ 168.55, 141.24, 117.33, 115.30, 59.91, 55.89, 51.94, 34.75, 32.31, 25.28, 24.86. IR (KBr) ν 3357, 3292, 3022, 2991, 2922, 2854, 1687, 1535, 1436, 1419, 1223, 1191, 1107, 875, 788. ESI$^+$ HRMS m/z calcd for $C_{22}H_{35}N_4O_4^+$ 419.2658, $[M + H]^+$, found 419.2690 $[M + H]^+$

Synthesis of *R*-6

R-**4** (223.5 mg, 0.535 mmol) and **5** (200 mg, 0.267 mmol) were added into 40 mL dried toluene. The reaction bottle was sealed and then was stirred at 110 °C for 12 hours under nitrogen protection. The product precipitated from the solution as the reaction proceeded. Excessive acetonitrile was added to make product completely precipitate and the solid was filtered and rinsed repeatedly with acetonitrile to furnish compound *R*-**6** (383.5 mg, 0.254 mmol 95%). $[\alpha]^{25}_{D}$ = – 664° ($CHCl_3$, c = 2 mg/mL). Mp > 300 °C. ^{1}H NMR (400 MHz, $CDCl_3$) δ 8.32 (s, 4H), 7.70 (d, *J* = 8 Hz, 8H), 7.50 (d, *J* = 8 Hz, 8H), 7.36 (s, 4H), 7.32 (d, *J* = 8 Hz, 8H), 7.08 (d, *J* = 8 Hz, 8H), 6.58 (d, *J* = 8 Hz, 3H), 3.82 (s, 12H), 3.41 (s, 5H), 3.08 (q, *J* = 8 Hz, 4H), 2.19 (d, *J* = 12 Hz, 4H), 1.92 – 1.73 (m, 16H), 1.60 (s, 6H), 1.42 (d, *J* = 8 Hz, 8H), 1.24 (s, 8H). ^{13}C NMR (101 MHz, $CDCl_3$) δ 168.61, 159.82, 141.30, 135.42, 131.62, 128.62, 126.55, 126.04, 116.82, 115.04, 58.01, 51.65, 25.29, 24.55. IR (KBr) ν 3353, 3080, 3027, 2988, 2926, 2853, 1907, 1690, 1643, 1572,1535, 1438, 1415, 1217, 1108, 973, 801, 727, 607, 500, 438. ESI$^+$ HRMS m/z calcd for $C_{98}H_{97}N_8O_8^+$ 1513.7429, $[M + H]^+$, found 1513.7476 $[M + H]^+$.

Synthesis of *S*-6

The synthetic procedure was the same as *R*-**6**. $[\alpha]^{25}_{D}$ = + 670° ($CHCl_3$, c = 2 mg/mL). Mp > 300 °C ^{1}H NMR (400 MHz, $CDCl_3$) δ 8.32 (s, 4H), 7.70 (d, *J* = 8.0 Hz, 8H), 7.50 (d, *J* = 8.0 Hz, 8H), 7.36 (s, 4H), 7.32 (d, *J* = 8.0 Hz, 8H), 7.08 (d, *J* = 8 Hz, 8H), 6.58 (d, J = 8 Hz, 3H), 3.82 (s, 12H), 3.41 (s, 4H), 3.08 (d, *J* = 8.0 Hz, 4H), 2.19 (d, *J* = 12 Hz, 5H), 1.87 – 1.71 (m, 16H), 1.62 (s, 4H), 1.42 (d, *J* = 8.0 Hz, 8H), 1.25 (s, 8H). ^{13}C NMR (101 MHz, $CDCl_3$) δ 168.61, 159.82, 141.30, 135.42, 131.62, 128.62, 126.55, 126.04, 116.82, 115.04, 58.01, 51.65, 25.29, 24.55. IR (KBr) ν 3353, 3080, 3027, 2988, 2926, 2853, 1907, 1690, 1643, 1572,1535, 1438, 1415, 1217, 1108, 973, 801, 727, 607, 500, 438. ESI$^+$ HRMS m/z calcd for $C_{98}H_{97}N_8O_8^+$ 1513.7429, $[M + H]^+$, found 1513.7485 $[M + H]^+$.

Synthesis of *R*-7

R-**6** (300 mg, 198 mmol) was dissolved into dried dichloromethane and excess sodium triacetoxyborohydride was then added. The reaction mixture was stirred at room temperature and was monitored by TLC until clear product spot appeared. At the end of the reaction, Na_2CO_3 solution was used to neutralize the reaction to be basic, and the separated organic phase was dried by Na_2SO_4 and concentrated. Then excessive acetonitrile was added to precipitate the product. After filtration, the filter cake was rinsed with methanol and acetonitrile to give pure *R*-**7** (298 mg, 196 mmol, 99%) as orange-red solid. $[\alpha]^{25}_{D}$ = – 602° ($CHCl_3$, c = 2 mg/mL). Mp > 300 °C. ^{1}H NMR (400 MHz, $CDCl_3$) δ 7.44 (d, *J* = 8 Hz, 8H), 7.35 (s, 4H), 7.31 (s, 4H), 7.29 (s, 8H), 7.27 (s, 4H), 7.09 (d, *J* = 8.0 Hz, 8H), 6.64 (d, *J* = 8 Hz, 4H), 3.97 (d, *J* = 8 Hz, 4H), 3.84 (s, 13H), 3.41 (d, *J* = 8 Hz, 4H), 3.30 (d, *J* = 8 Hz, 4H), 2.51 (s, 4H), 2.29 (d, *J* = 8 Hz, 4H), 2.01 (d, *J* = 12 Hz, 6H), 1.93 (s, 4H), 1.83 – 1.71 (m, 8H), 1.33 (s, 8H), 1.23 (d, *J* = 16 Hz, 8H). ^{13}C NMR (101 MHz, $CDCl_3$) δ 168.54, 142.36, 141.12, 139.37, 139.30, 138.80, 131.69, 129.27, 126.85, 126.11, 117.27, 114.80, 63.54, 56.79, 52.53, 51.76, 33.06, 31.11, 25.35, 24.45.

IR (KBr) *ν* 3340, 5025, 2991, 2924, 2853, 2805, 1905, 1691, 1534, 1437, 1319, 1216, 1103, 1004, 812, 790,619, 577. ESI$^+$ HRMS m/z calcd for $C_{98}H_{105}N_8O_8^+$ 1522.8089, [M + H]$^+$, found 1522.7991 [M + H]$^+$.

Synthesis of *S*-7

The synthetic procedure was the same as *R*-**7**. $[\alpha]^{25}_D$ = + 606° ($CHCl_3$, c = 2 mg/mL). Mp > 300 °C. ^{1}H NMR (400 MHz, $CDCl_3$) *δ* 7.43 (d, *J* = 8 Hz, 8H), 7.35 (s, 4H), 7.31 (s, 4H), 7.29 (s, 8H), 7.27 (s, 4H), 7.08 (d, *J* = 8.0 Hz, 8H), 6.64 (d, *J* = 8 Hz, 4H), 3.98 (d, *J* = 8 Hz, 4H), 3.84 (s, 12H), 3.41 (d, *J* = 8 Hz, 4H), 3.36 – 3.23 (m, 4H), 2.51 (s, 4H), 2.29 (s, 4H), 2.17 (s, 6H), 2.00 (d, *J* = 12Hz, 4H), 1.77 (m, 8H), 1.33 (s, 8H), 1.22 (d, *J* = 16 Hz, 8H). ^{13}C NMR (101 MHz, $CDCl_3$) *δ* 168.54, 142.36, 141.12, 139.37, 139.30, 138.80, 131.69, 129.27, 126.85, 126.11, 117.27, 114.80, 63.54, 56.79, 52.53, 51.76, 33.06, 31.11, 25.35, 24.45. IR (KBr) *ν* 3340, 5025, 2991, 2924, 2853, 2805, 1905, 1691, 1534, 1437, 1319, 1216, 1103, 1004, 812, 790,619, 577. ESI$^+$ HRMS m/z calcd for $C_{98}H_{105}N_8O_8^+$ 1522.8089, [M + H]$^+$, found 1522.8055 [M + H]$^+$.

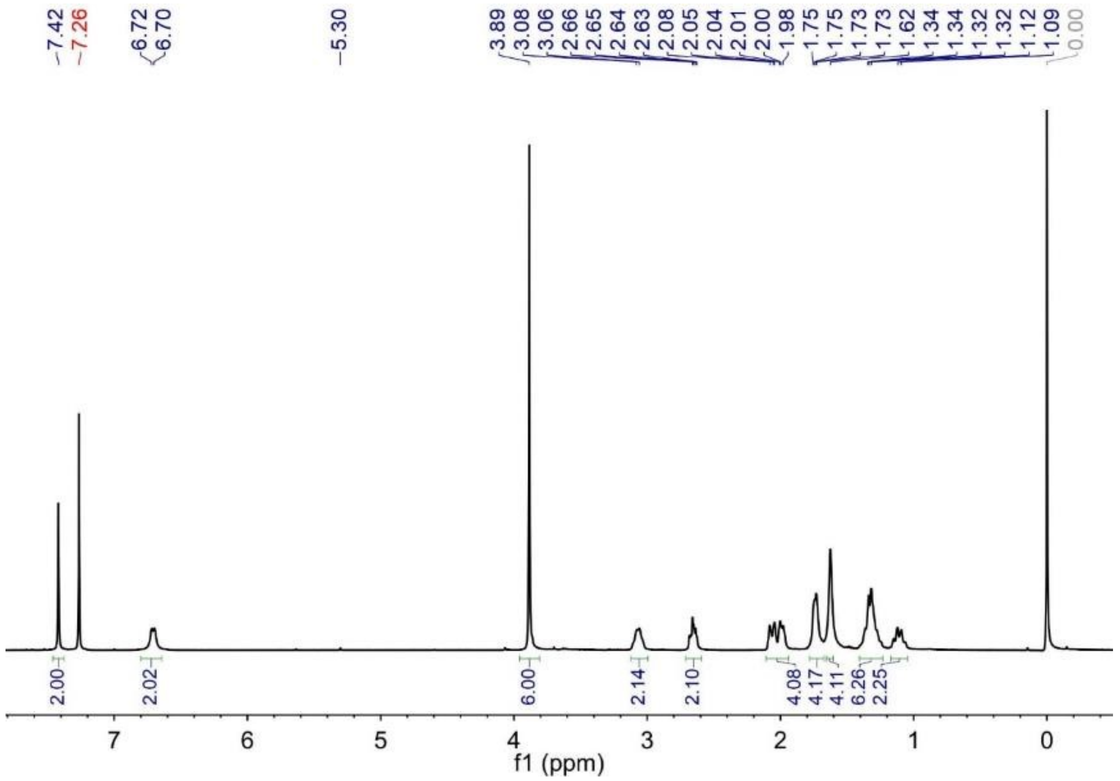

Fig. S1. ^{1}H NMR spectrum of compound *R*-**4** in $CDCl_3$.

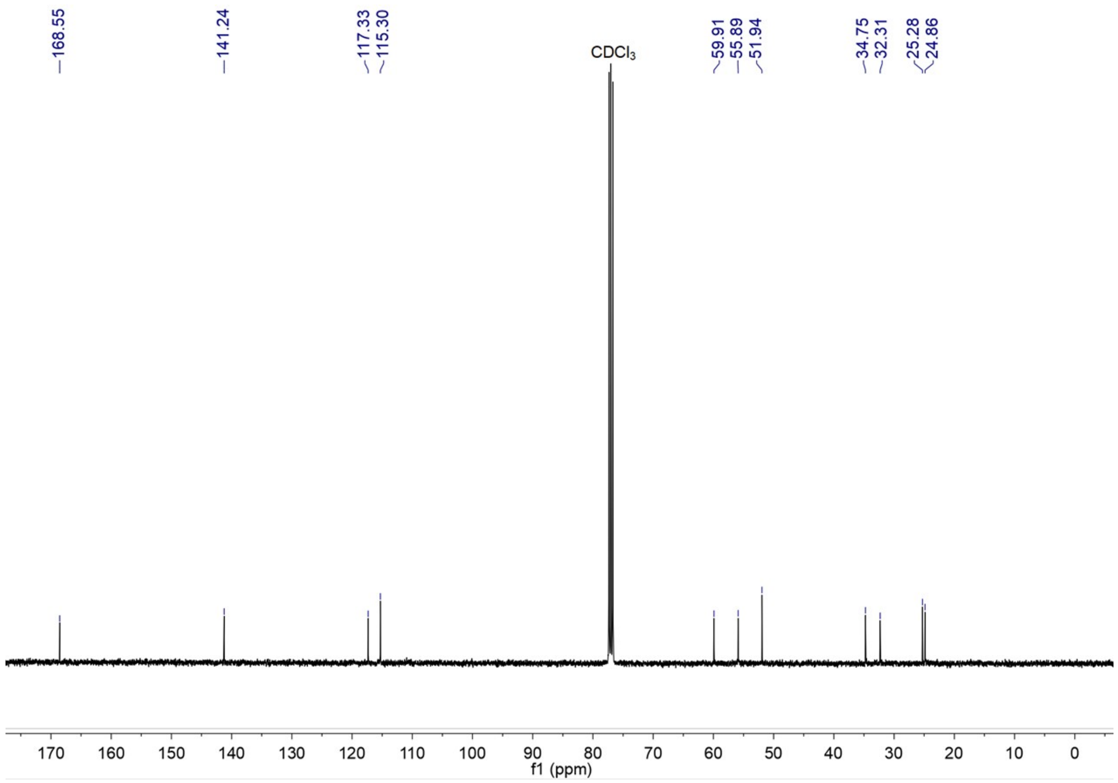

Fig. S2. ^{13}C NMR spectrum of compound *R*-**4** in $CDCl_3$.

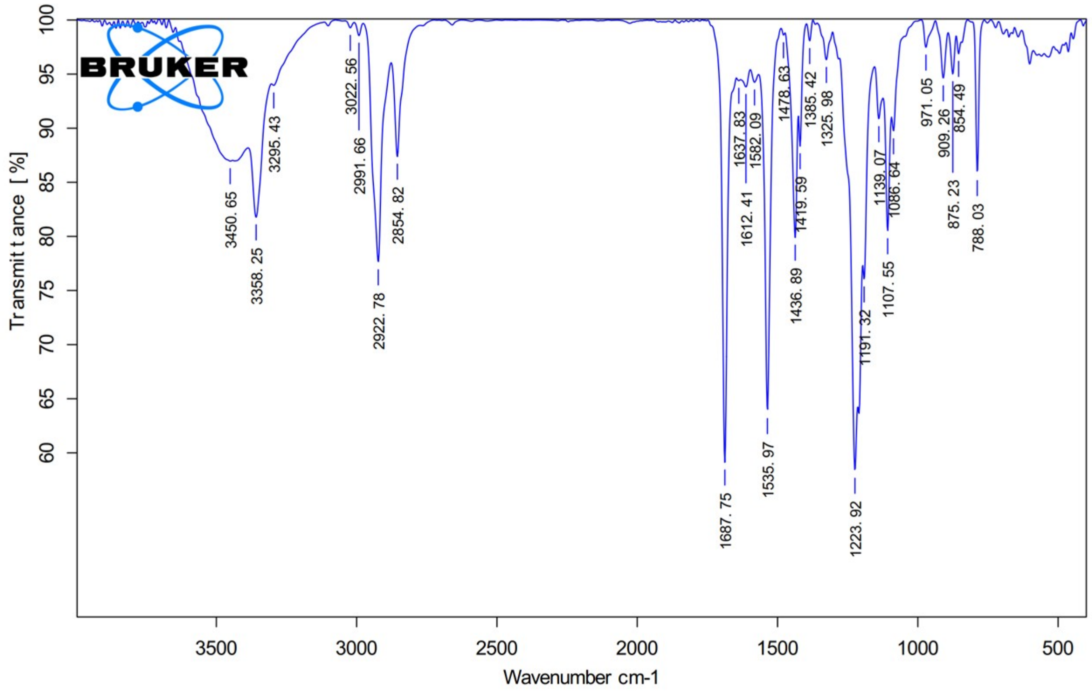

Fig. S3. IR spectrum of compound *R*-**4**.

Mass Spectrum List Report

Analysis Info

Analysis Name	D:\Data\ZhengYS\zheng-huming20230914-H1.d	Acquisition Date	9/14/2023 11:12:51 AM
Method	tune_wide.m	Operator	BDAL@DE
Sample Name	zheng-huming20230914-H1	Instrument / Ser#	micrOTOF 10401
Comment			

Acquisition Parameter

Source Type	ESI	Ion Polarity	Positive	Set Nebulizer	0.3 Bar
Focus	Active			Set Dry Heater	180 °C
Scan Begin	50 m/z	Set Capillary	4500 V	Set Dry Gas	4.0 l/min
Scan End	3000 m/z	Set End Plate Offset	-500 V	Set Divert Valve	Waste

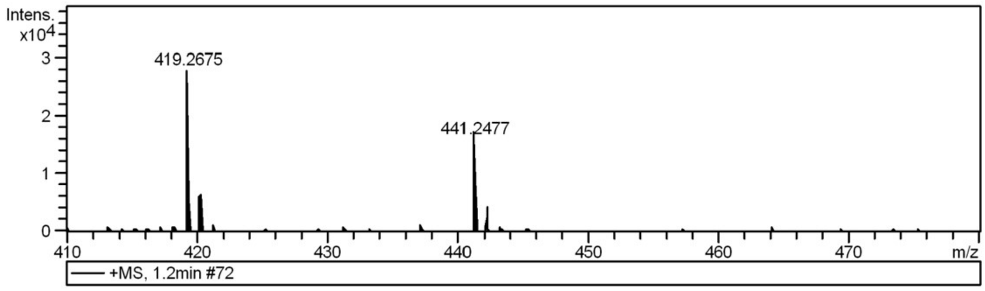

#	m/z	Res.	S/N	I	FWHM
1	419.2675	7257	139.7	27989	0.0578
2	420.2707	5811	32.8	6567	0.0723
3	441.2477	6106	79.0	16094	0.0723
4	442.2534	5708	21.2	4324	0.0775

Fig. S4. HRMS spectrum of compound *R*-**4**.

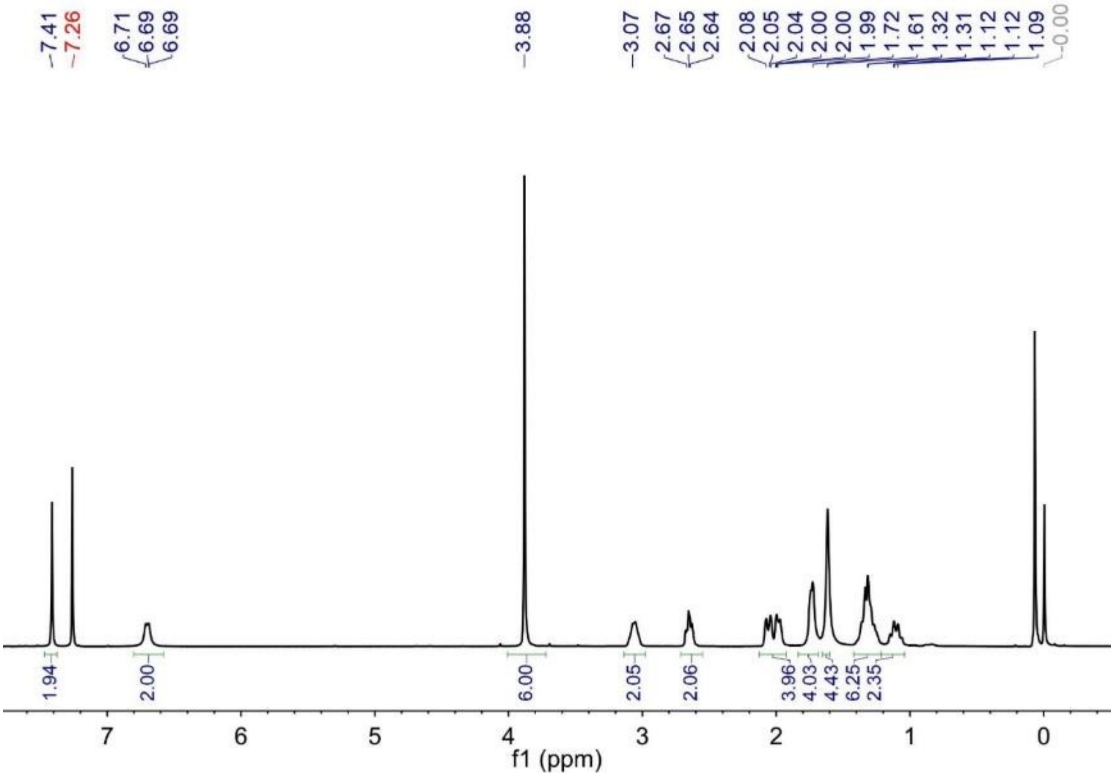

Fig. S5. ^{1}H NMR spectrum of compound *S*-**4** in $CDCl_3$.

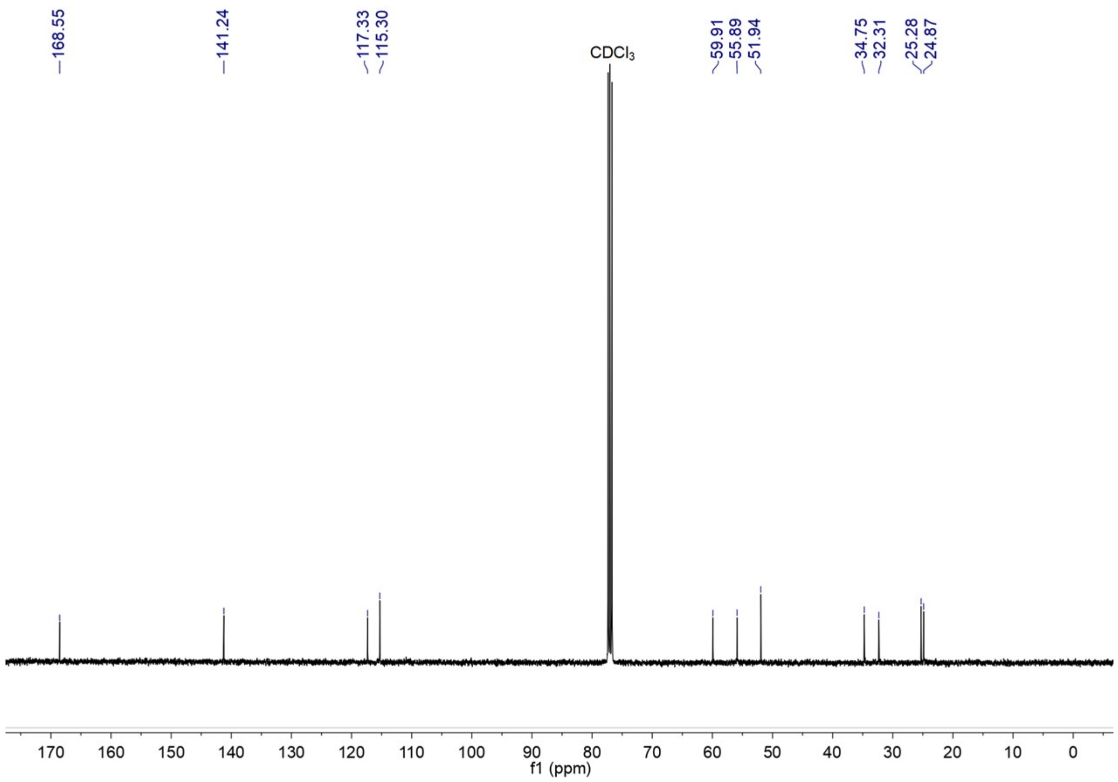

Fig. S6. ^{13}C NMR spectrum of compound *S*-**4** in $CDCl_3$.

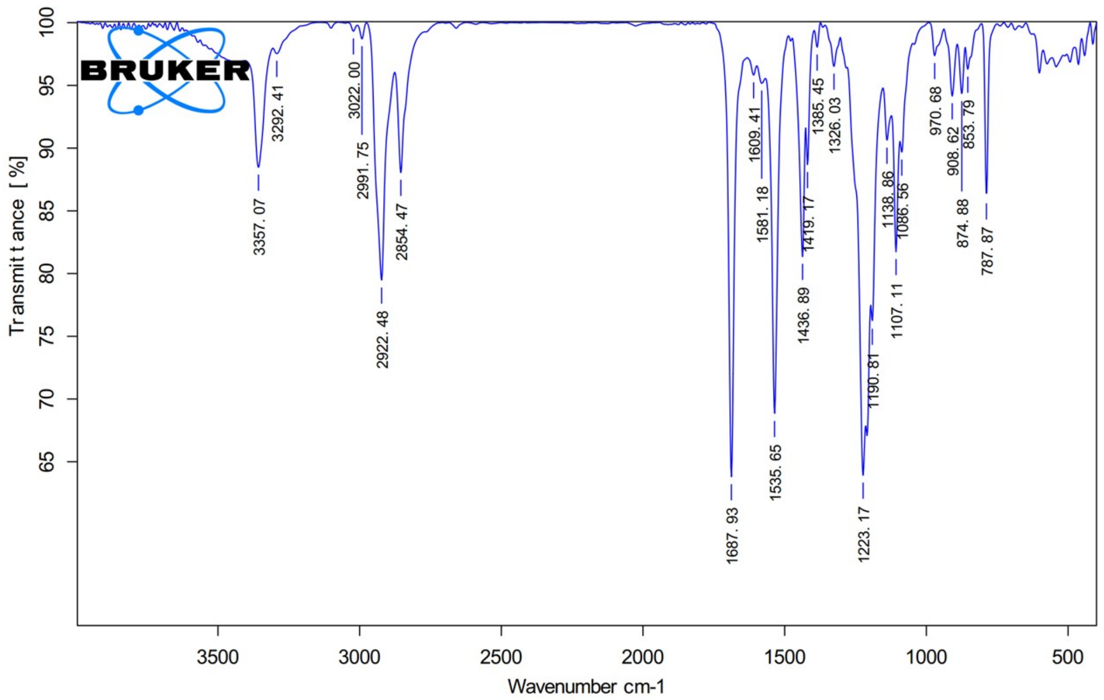

Fig. S7. IR spectrum of compound *S*-**4**.

Mass Spectrum List Report

Analysis Info

Analysis Name	D:\Data\ZhengYS\zheng-huming20231009-1.d	Acquisition Date	10/9/2023 2:47:37 PM
Method	tune_wide.m	Operator	BDAL@DE
Sample Name	zheng-huming20231009-1	Instrument / Ser#	micrOTOF 10401
Comment			

Acquisition Parameter

Source Type	ESI	Ion Polarity	Positive	Set Nebulizer	0.3 Bar
Focus	Active			Set Dry Heater	180 °C
Scan Begin	50 m/z	Set Capillary	4500 V	Set Dry Gas	4.0 l/min
Scan End	3000 m/z	Set End Plate Offset	-500 V	Set Divert Valve	Waste

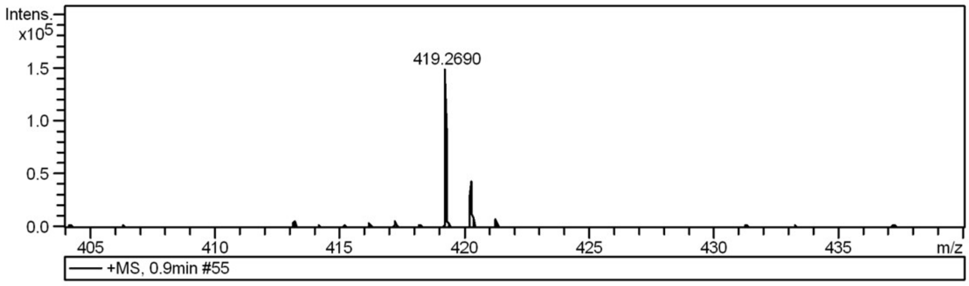

#	m/z	Res.	S/N	I	FWHM
1	419.2690	8213	1880.8	149487	0.0510
2	420.2677	6598	534.0	42446	0.0637
3	421.2679	6614	103.5	8226	0.0637

Fig. S8. HRMS spectrum of compound *S*-**4**.

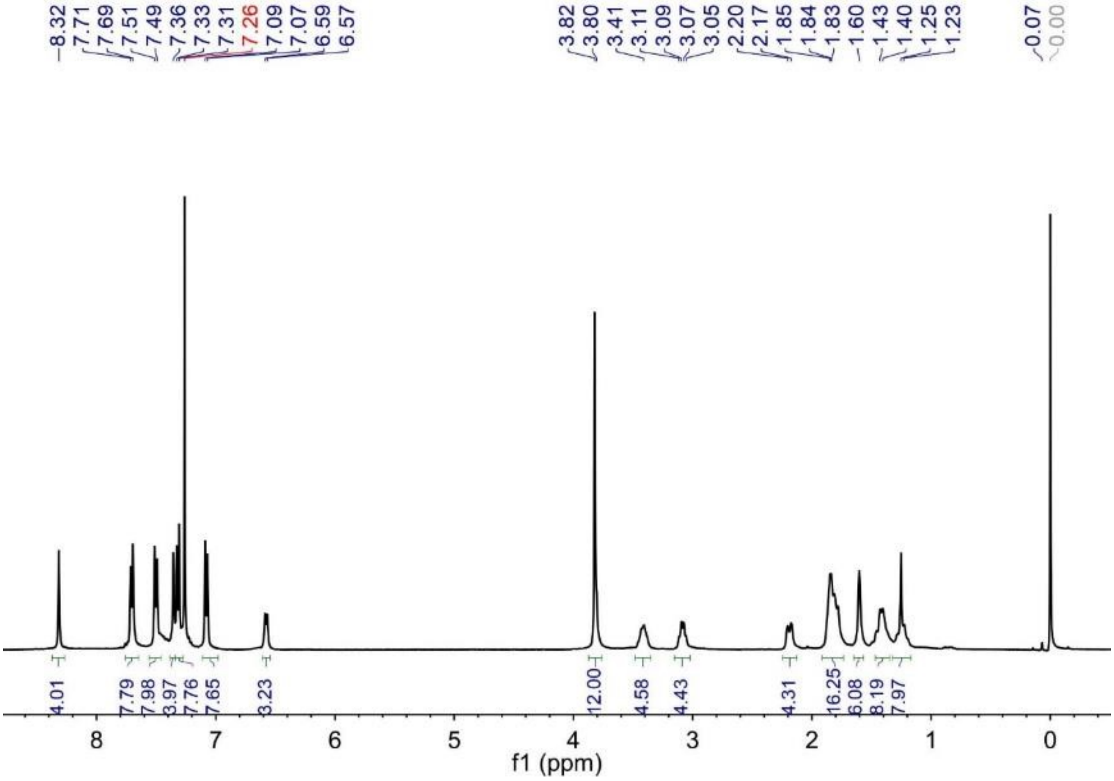

Fig. S9. ^{1}H NMR spectrum of compound *R*-**6** in $CDCl_3$.

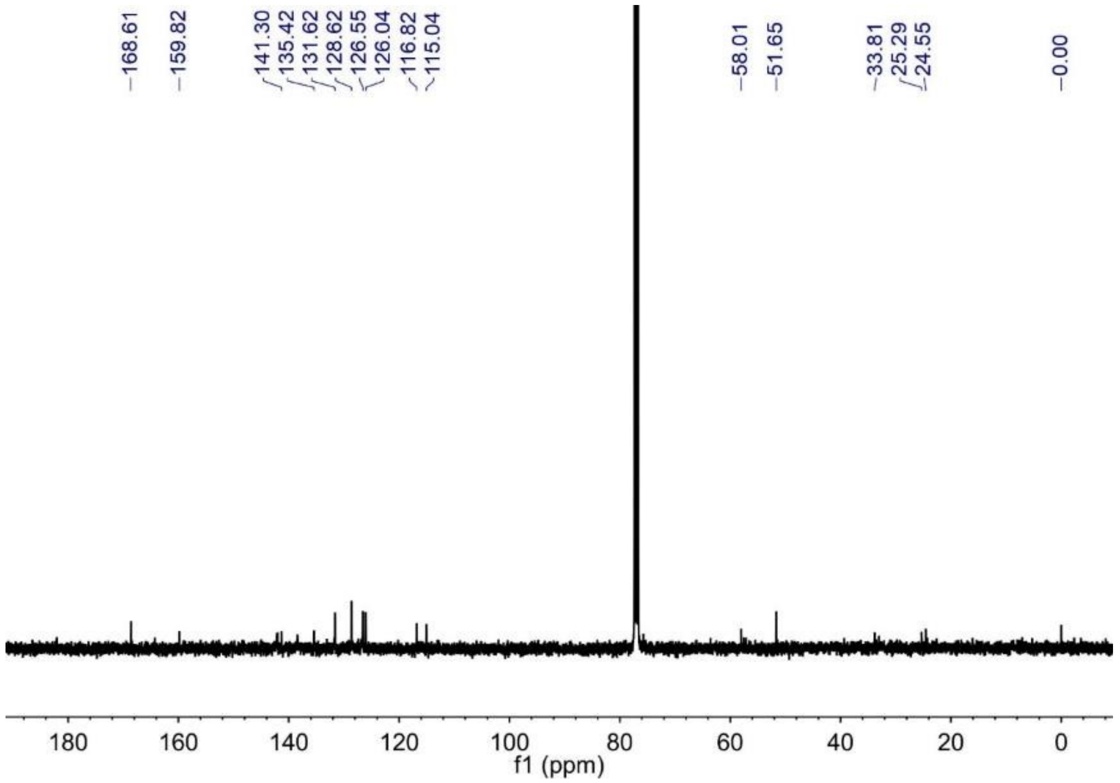

Fig. S10. ^{13}C NMR spectrum of compound *R*-**6** in $CDCl_3$.

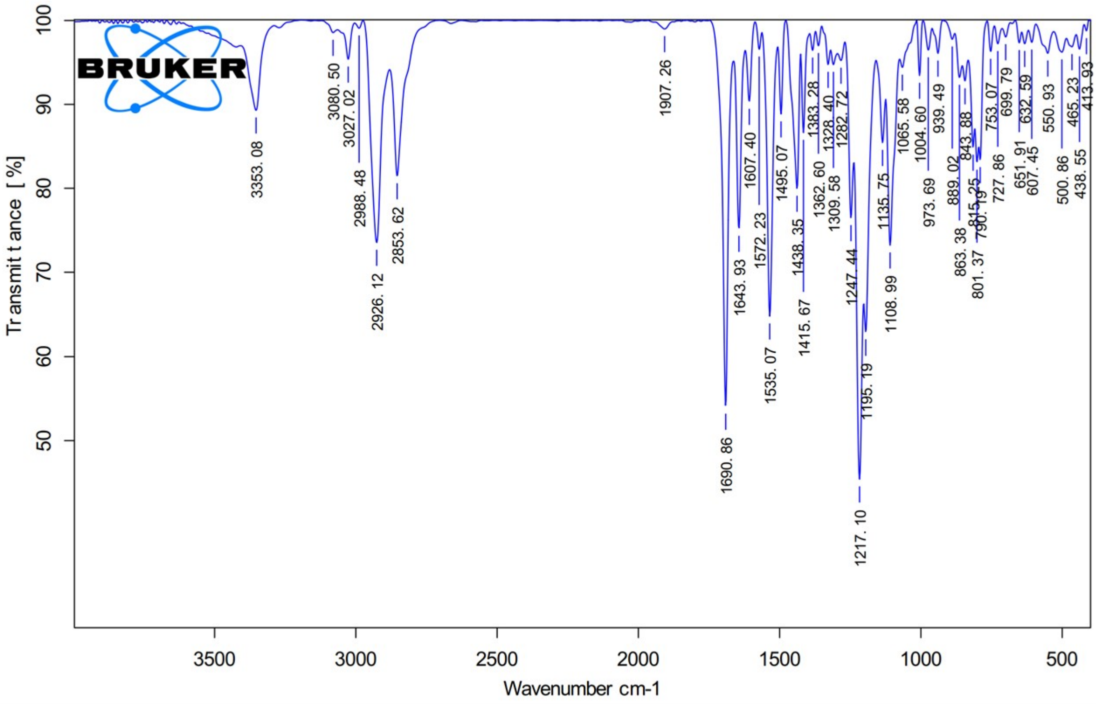

Fig. S11. IR spectrum of compound *R*-**6**.

Mass Spectrum List Report

Analysis Info

Analysis Name	D:\Data\ZhengYS\zheng-huming20230914-H5.d	Acquisition Date	9/14/2023 4:22:03 PM
Method	tune_wide.m	Operator	BDAL@DE
Sample Name	zheng-huming20230914-H5	Instrument / Ser#	micrOTOF 10401
Comment			

Acquisition Parameter

Source Type	ESI	Ion Polarity	Positive	Set Nebulizer	0.3 Bar
Focus	Active			Set Dry Heater	180 °C
Scan Begin	50 m/z	Set Capillary	4500 V	Set Dry Gas	4.0 l/min
Scan End	3000 m/z	Set End Plate Offset	-500 V	Set Divert Valve	Waste

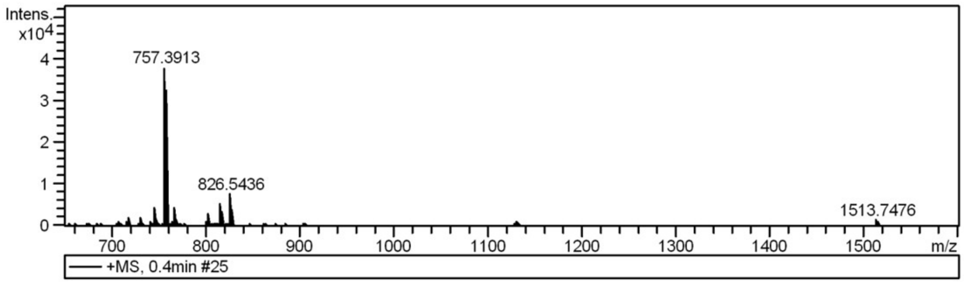

#	m/z	Res.	S/N	I	FWHM
1	745.3393	7159	27.3	3988	0.1041
2	757.3913	5987	240.3	34545	0.1265
3	757.8953	5934	225.4	32389	0.1277
4	758.3972	5959	122.5	17585	0.1273
5	758.8989	5527	44.0	6316	0.1373
6	766.3978	6206	28.2	4010	0.1235
7	802.9029	7038	22.1	2996	0.1141
8	826.5436	7407	58.1	7616	0.1116
9	1513.7476	8237	23.6	1456	0.1838
10	1514.7663	7039	22.2	1345	0.2152
11	1515.7614	8171	11.8	716	0.1855

Fig. S12. HRMS spectrum of compound *R*-**6**.

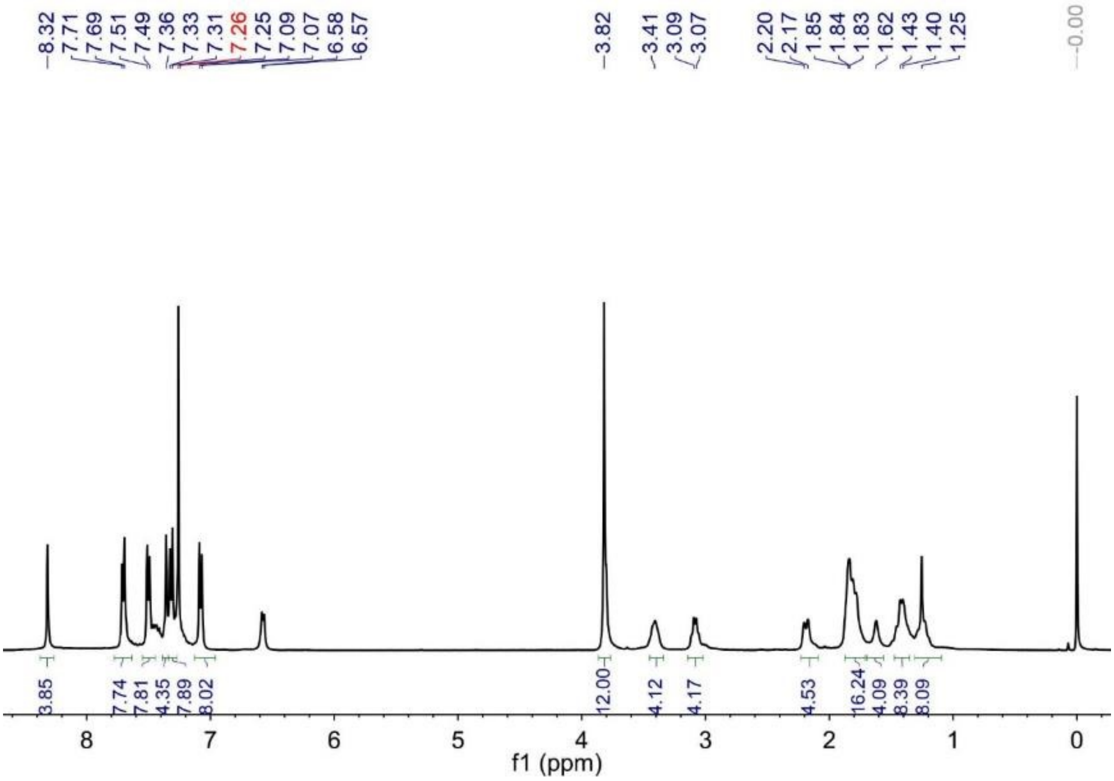

Fig. S13. ^{1}H NMR spectrum of compound *S*-**6** in $CDCl_3$.

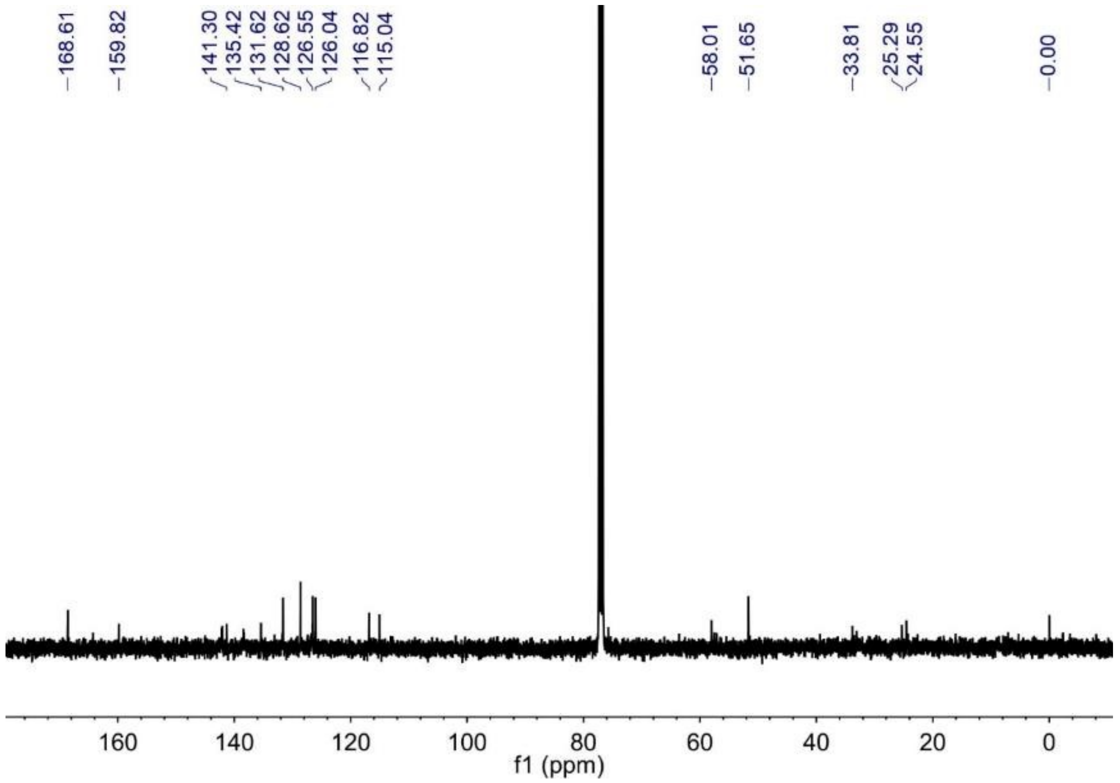

Fig. S14. ^{13}C NMR spectrum of compound *S*-**6** in $CDCl_3$.

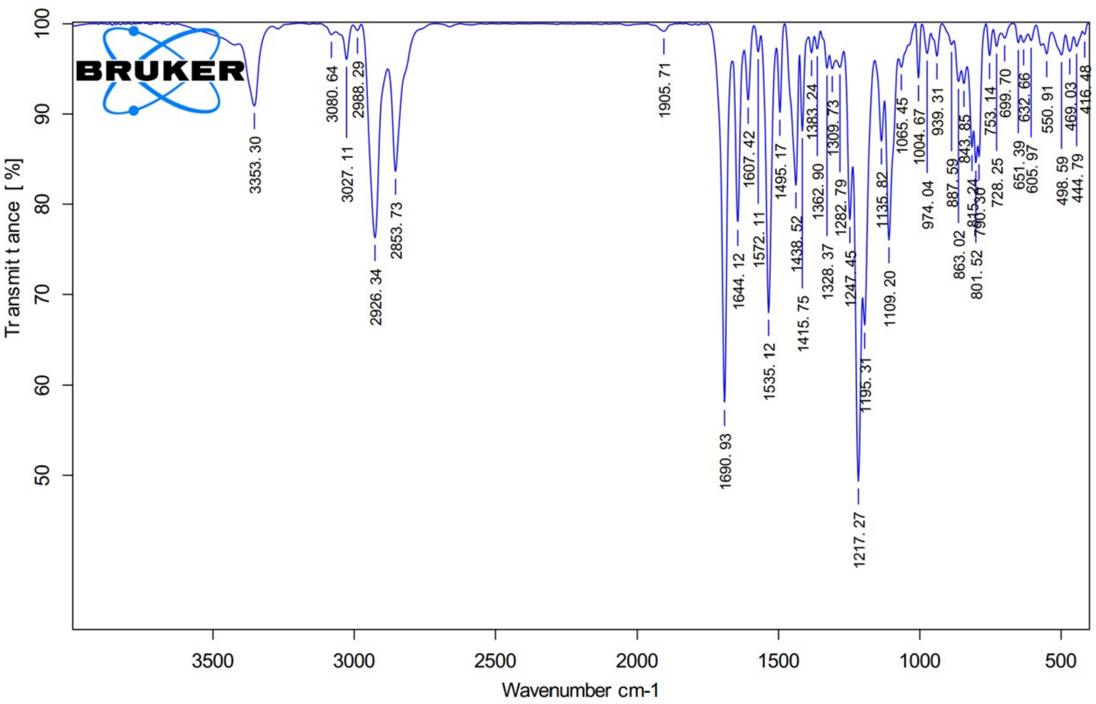

Fig. S15. IR spectrum of compound *S*-**6**.

Mass Spectrum List Report

Analysis Info

Analysis Name	D:\Data\ZhengYS\zheng-huming20231009-2.d	Acquisition Date	10/9/2023 2:54:01 PM
Method	tune_wide.m	Operator	BDAL@DE
Sample Name	zheng-huming20231009-2	Instrument / Ser#	micrOTOF 10401
Comment			

Acquisition Parameter

Source Type	ESI	Ion Polarity	Positive	Set Nebulizer	0.3 Bar
Focus	Active			Set Dry Heater	180 °C
Scan Begin	50 m/z	Set Capillary	4500 V	Set Dry Gas	4.0 l/min
Scan End	3000 m/z	Set End Plate Offset	-500 V	Set Divert Valve	Waste

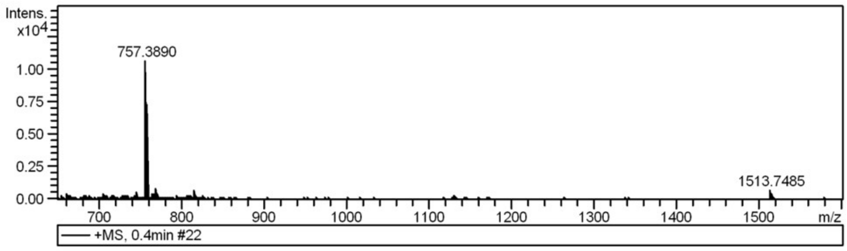

#	m/z	Res.	S/N	I	FWHM
1	756.3792	5740	48.8	3916	0.1318
2	756.8881	5964	118.7	8808	0.1269
3	757.3890	5500	143.7	10655	0.1377
4	757.8934	4976	99.0	7333	0.1523
5	758.3959	5089	45.4	3359	0.1490
6	758.8878	7093	19.4	1435	0.1070
7	1512.7552	7555	8.8	328	0.2002
8	1513.7485	7268	19.6	730	0.2083
9	1514.7478	7160	10.5	390	0.2115
10	1515.7697	10957	6.3	236	0.1383
11	1516.8078	13156	4.2	155	0.1153

Fig. S16. HRMS spectrum of compound *S*-**6**.

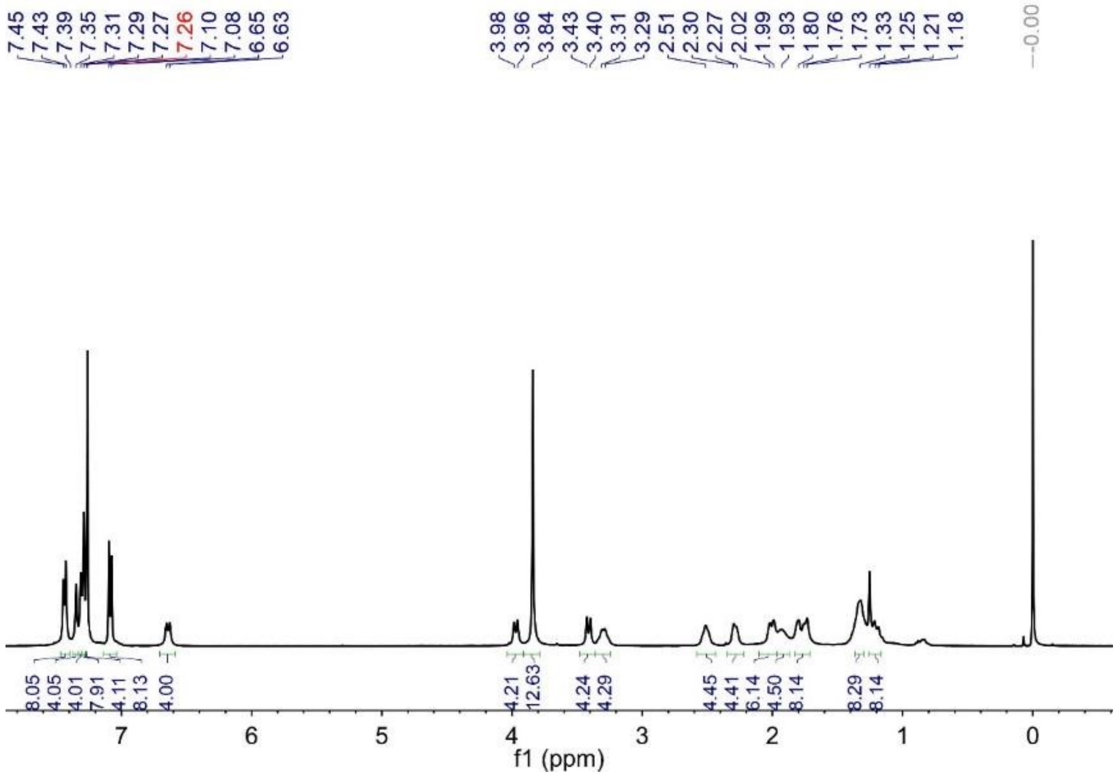

Fig. S17. ^{1}H NMR spectrum of compound *R*-**7** in $CDCl_3$.

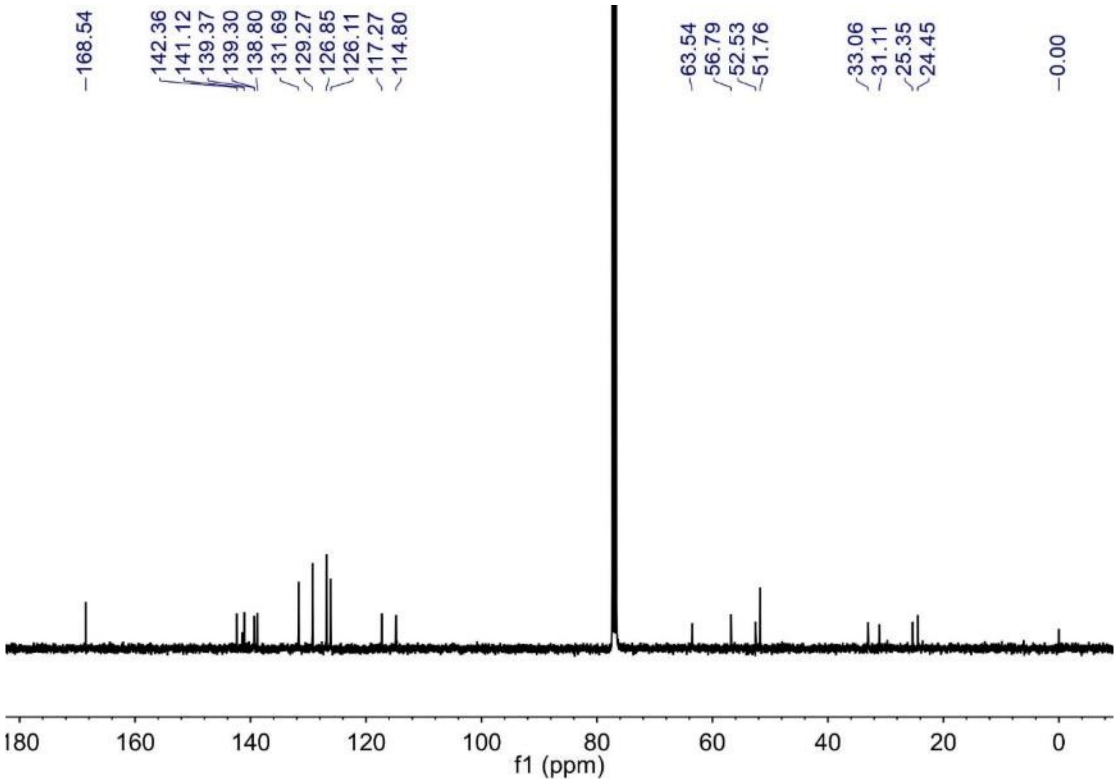

Fig. S18. ^{13}C NMR spectrum of compound *R*-**7** in $CDCl_3$.

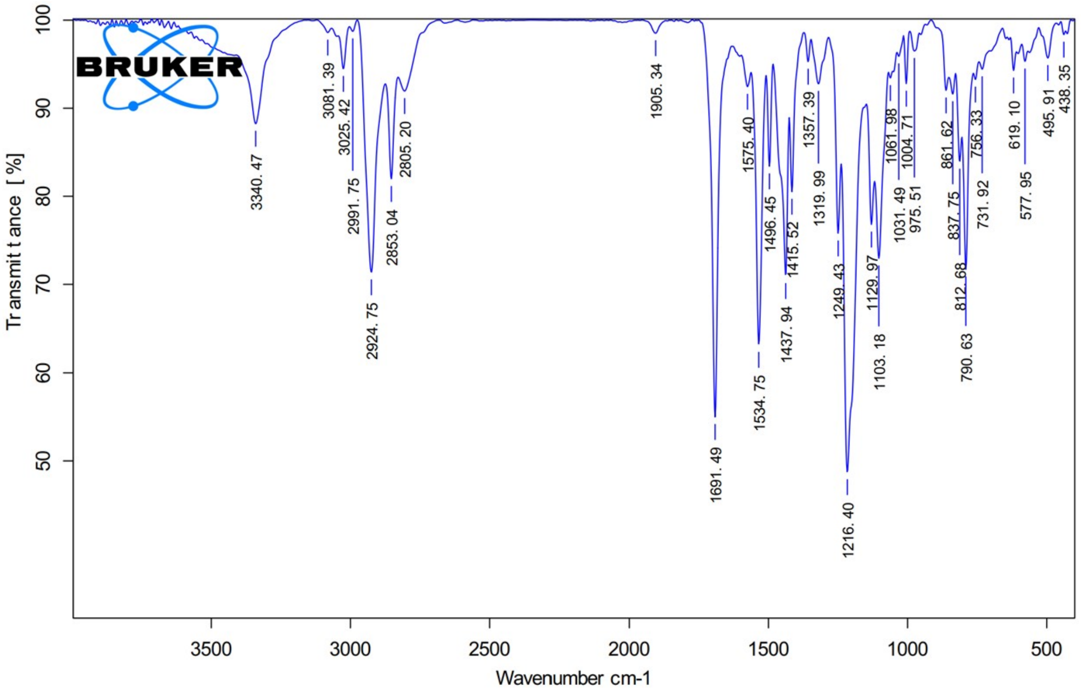

Fig. S19. IR spectrum of compound *R*-**7**.

Mass Spectrum List Report

Analysis Info

Analysis Name	D:\Data\ZhengYS\zheng-huming20230914-H6-1.d	Acquisition Date	9/14/2023 4:53:34 PM
Method	tune_wide.m	Operator	BDAL@DE
Sample Name	zheng-huming20230914-H6-1	Instrument / Ser#	micrOTOF 10401
Comment			

Acquisition Parameter

Source Type	ESI	Ion Polarity	Positive	Set Nebulizer	0.3 Bar
Focus	Active			Set Dry Heater	180 °C
Scan Begin	50 m/z	Set Capillary	4500 V	Set Dry Gas	4.0 l/min
Scan End	3000 m/z	Set End Plate Offset	-500 V	Set Divert Valve	Waste

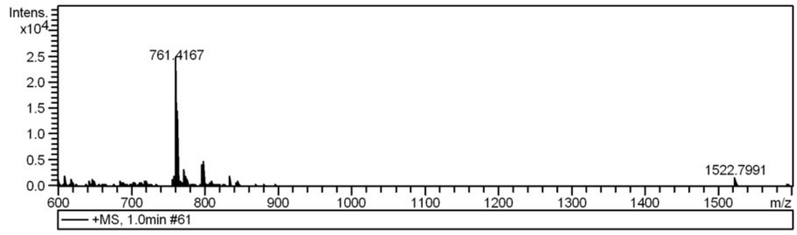

#	m/z	Res.	S/N	I	FWHM
1	761.4167	6179	236.7	23659	0.1232
2	761.9201	5863	231.1	23077	0.1300
3	762.4210	5810	144.8	14446	0.1312
4	762.9222	5903	56.4	5623	0.1293
5	772.4075	6215	29.6	2911	0.1243
6	772.9104	5246	26.2	2571	0.1473
7	797.4301	6776	44.4	4194	0.1177
8	797.9297	6604	49.2	4649	0.1208
9	833.9413	5529	18.4	1632	0.1508
10	1521.8028	6701	24.6	1255	0.2271
11	1522.7991	8226	31.5	1567	0.1851
12	1523.8131	7986	17.1	848	0.1908

Fig. S20. HRMS spectrum of compound *R*-**7**.

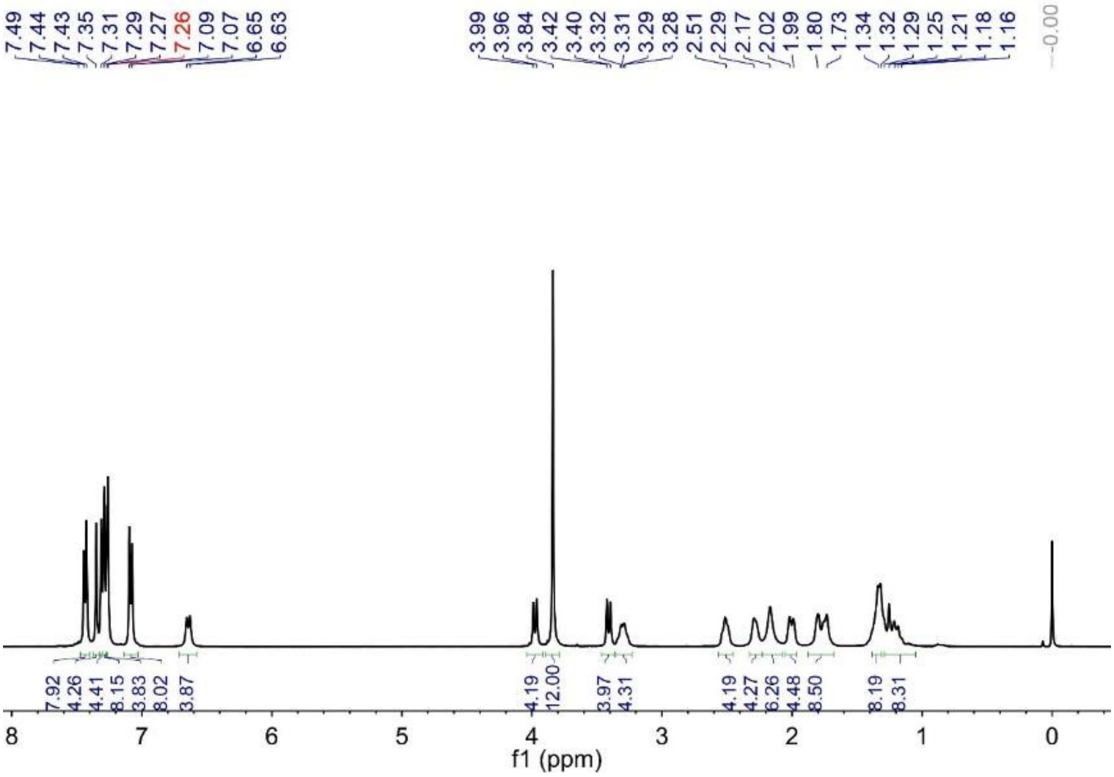

Fig. S21. ^{1}H NMR spectrum of compound *S*-**7** in $CDCl_3$.

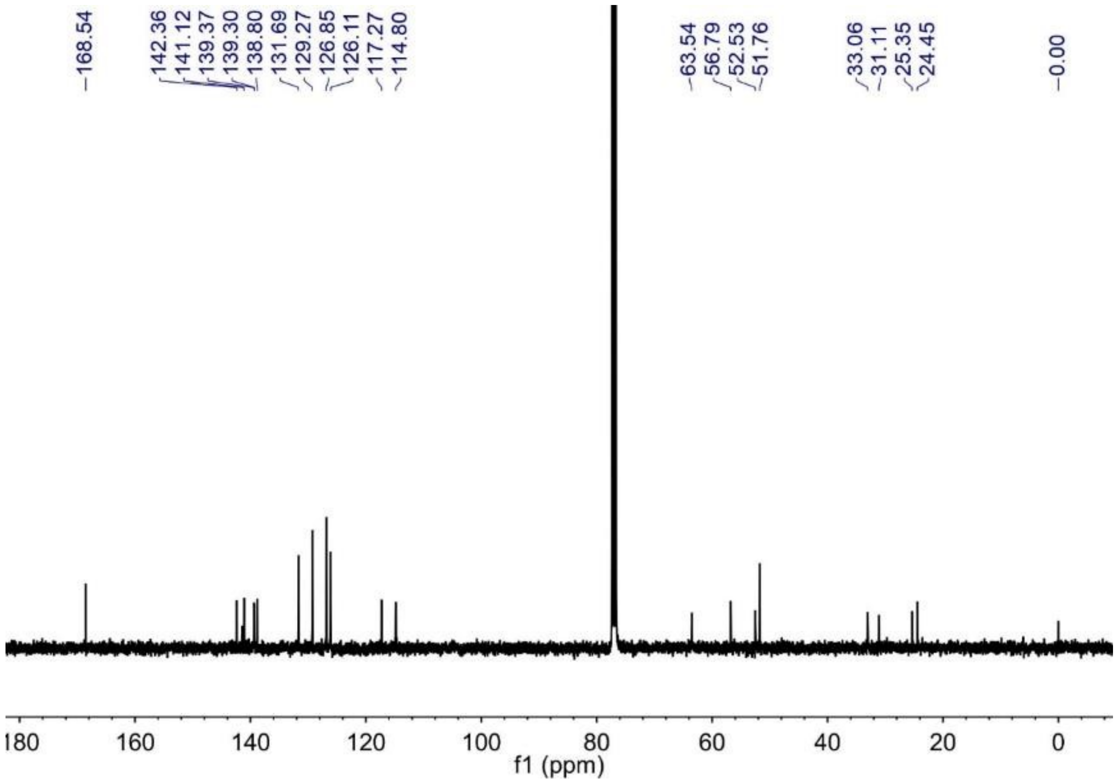

Fig. S22. ^{13}C NMR spectrum of compound *S*-**7** in $CDCl_3$.

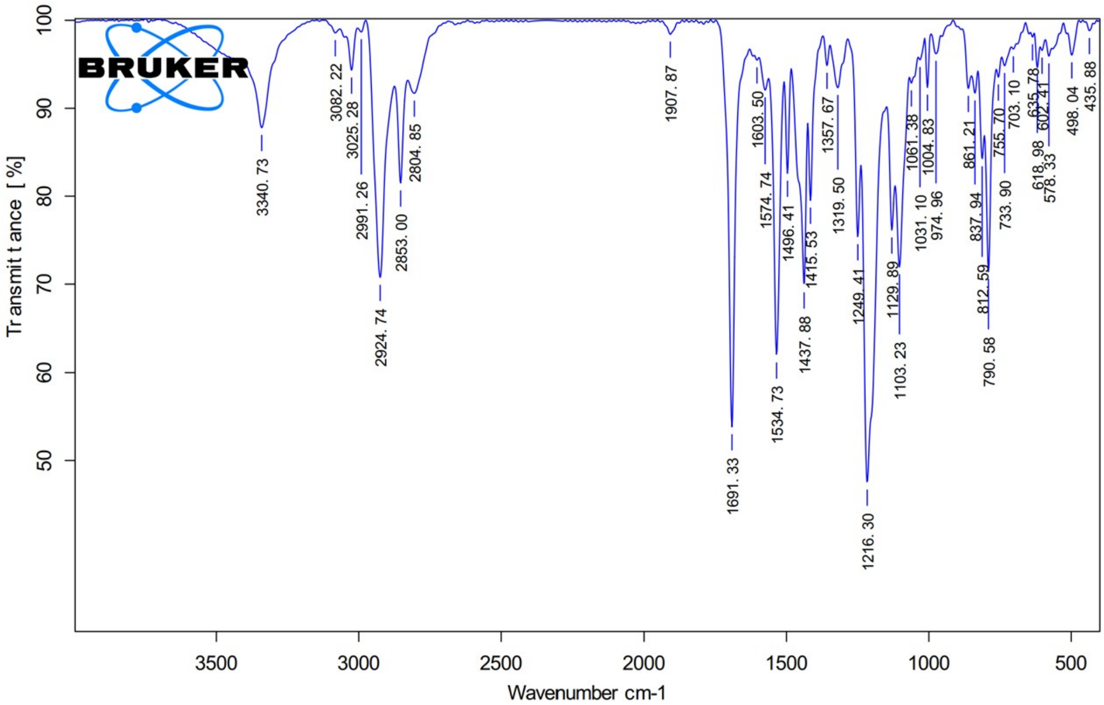

Fig. S23. IR spectrum of compound *S*-**7**.

Mass Spectrum List Report

Analysis Info

Analysis Name	D:\Data\ZhengYS\zheng-huming20231009-3.d	Acquisition Date	10/9/2023 3:00:59 PM
Method	tune_wide.m	Operator	BDAL@DE
Sample Name	zheng-huming20231009-3	Instrument / Ser#	micrOTOF 10401
Comment			

Acquisition Parameter

Source Type	ESI	Ion Polarity	Positive	Set Nebulizer	0.3 Bar
Focus	Active			Set Dry Heater	180 °C
Scan Begin	50 m/z	Set Capillary	4500 V	Set Dry Gas	4.0 l/min
Scan End	3000 m/z	Set End Plate Offset	-500 V	Set Divert Valve	Waste

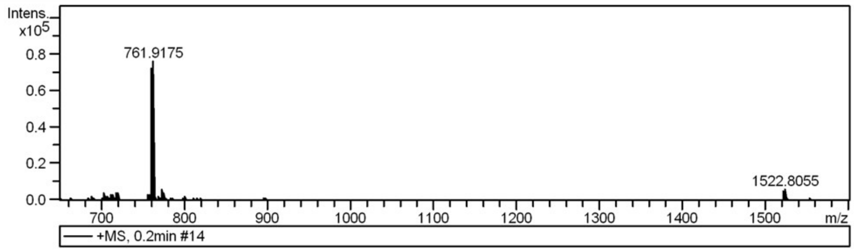

#	m/z	Res.	S/N	I	FWHM
1	761.4163	5828	410.9	66830	0.1307
2	761.9175	6545	467.8	76016	0.1164
3	762.4191	5828	263.5	42770	0.1308
4	762.9202	5231	110.2	17867	0.1459
5	1521.7936	7591	62.1	5103	0.2005
6	1522.8055	5384	68.9	5631	0.2829
7	1523.8079	8257	35.9	2941	0.1845
8	1524.8108	6484	14.5	1186	0.2352

Fig. S24. HRMS spectrum of compound *S*-**7**.

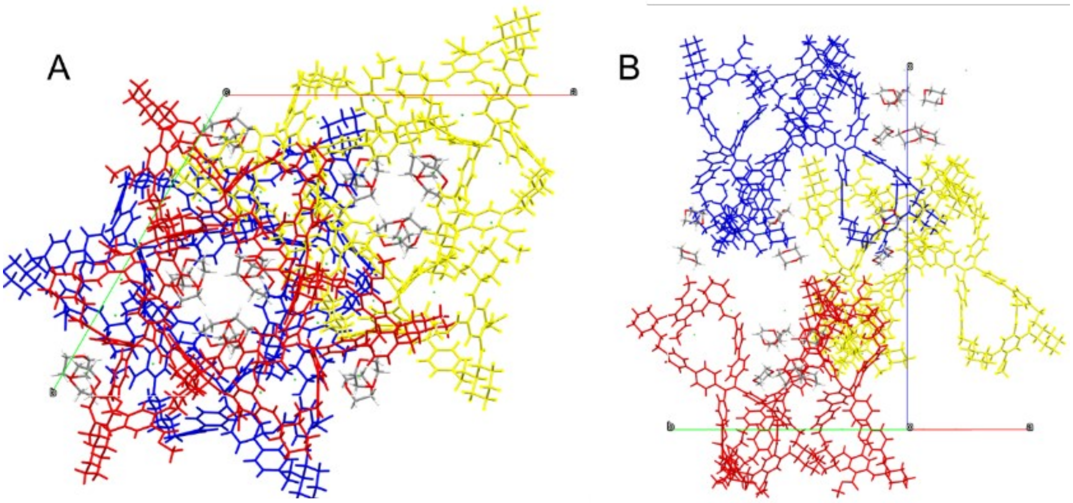

Fig. S25. The top view (A) and side view (B) of crystal cell of *R*-**7** hydrochloride (solvent molecules: 1,4-dioxane).

CD, CPL and SEM of TPE bimacrocycle 6

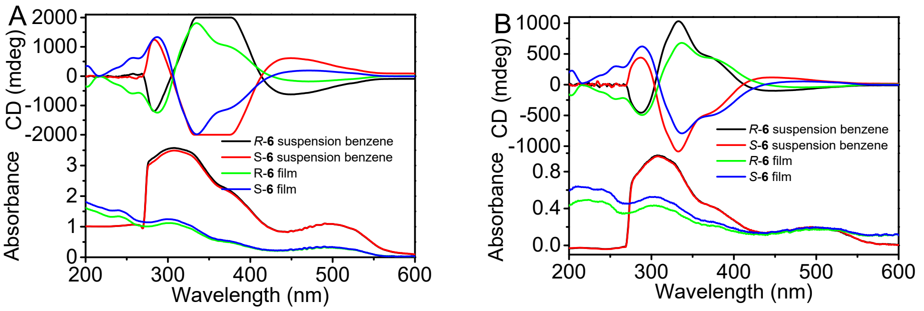

Fig. S26. CD and absorption spectra of suspension of *R*-**6** and *S*-**6** in benzene and in a drop-coating film (A) at 1.0 × 10^{-3} M and (B) at 2.0 × 10^{-4} M.

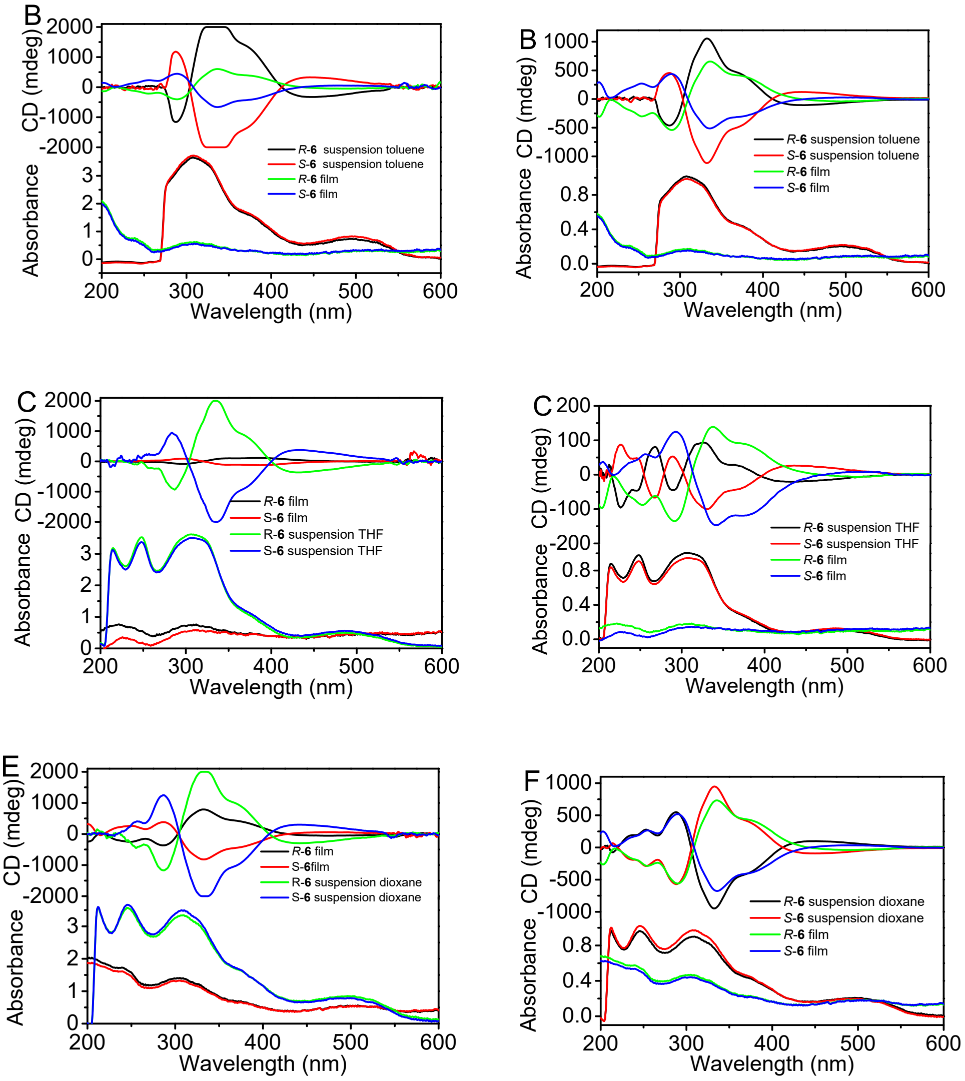

Fig. S27. CD and absorption spectra of suspension of *R*-**6** and *S*-**6** in toluene at 5.0 × 10^{-4} M (A) and 2.0 × 10^{-4} M (B), in THF at 5.0 × 10^{-4} M (C) and 2.0 × 10^{-4} M (D), in 1,4-dioxane at 5.0 × 10^{-4} M (E) and 2.0 × 10^{-4} M (F) as well as in a drop-coating film prepared from corresponding suspension.

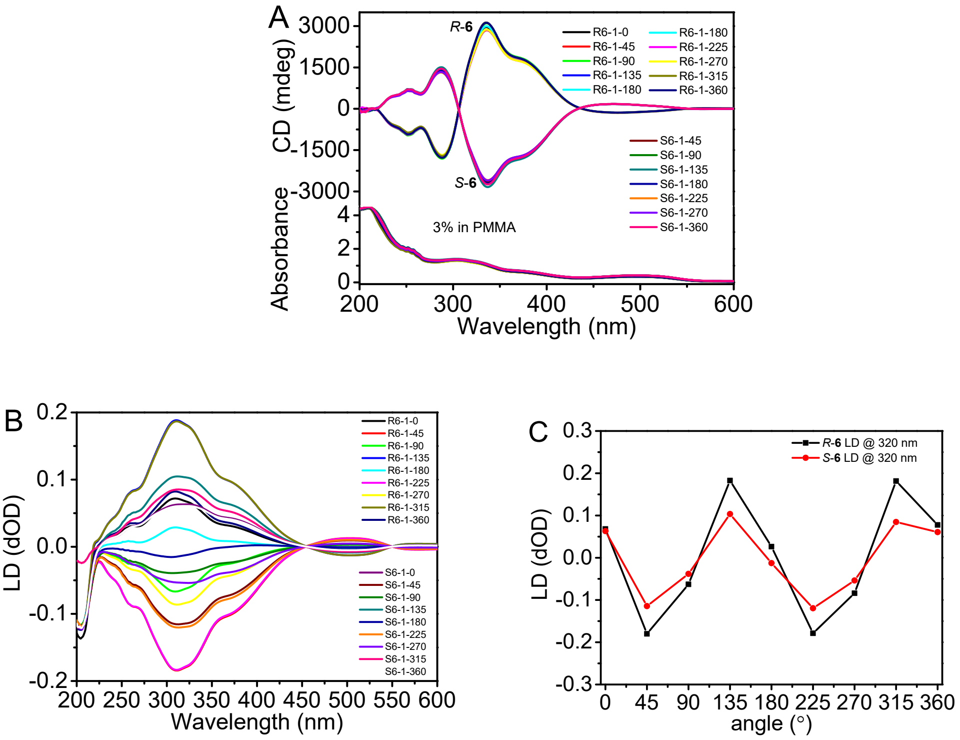

Fig. S28. Change in CD (A) and LD (B) spectra of *R*-**6** / *S*-**6** in PMMA films with different angles, and Intensity of LD vs angles (C). 3 weight% of **6** in PMMA.

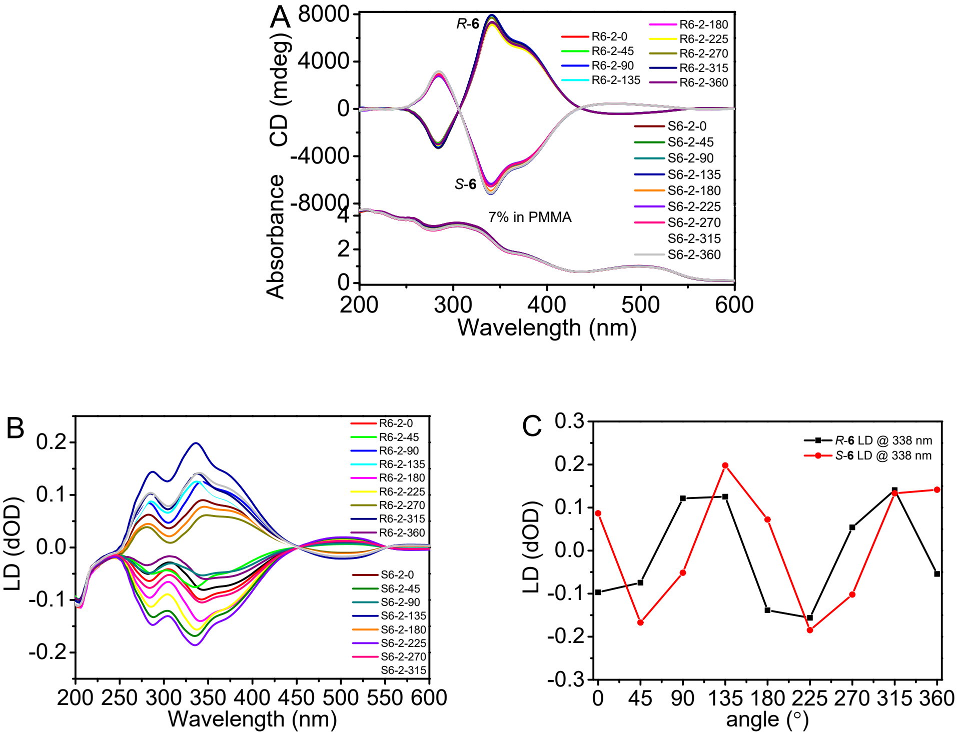

Fig. S29. Change in CD (A) and LD (B) spectra of *R*-**6** / *S*-**6** in PMMA films with different angles, and Intensity of LD vs angles (C). 7 weight% of **6** in PMMA.

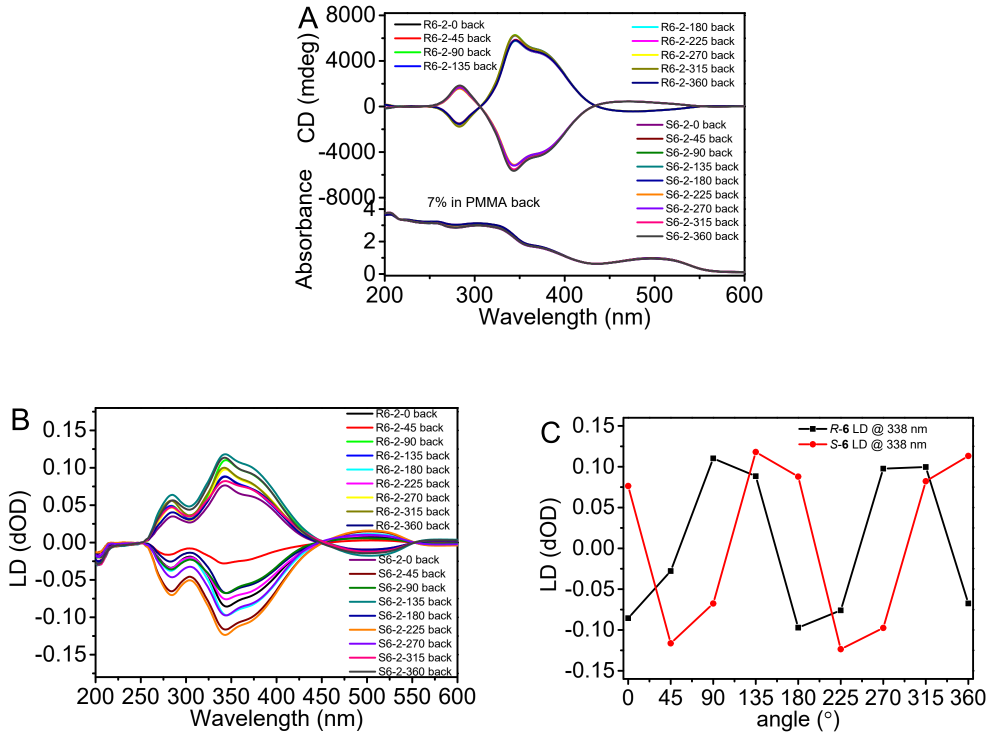

Fig. S30. In the back of the PMMA film, change in CD (A) and LD (B) spectra of *R*-**6** / *S*-**6** in PMMA films with different angles, and Intensity of LD vs angles (C). 7 weight% of **6** in PMMA.

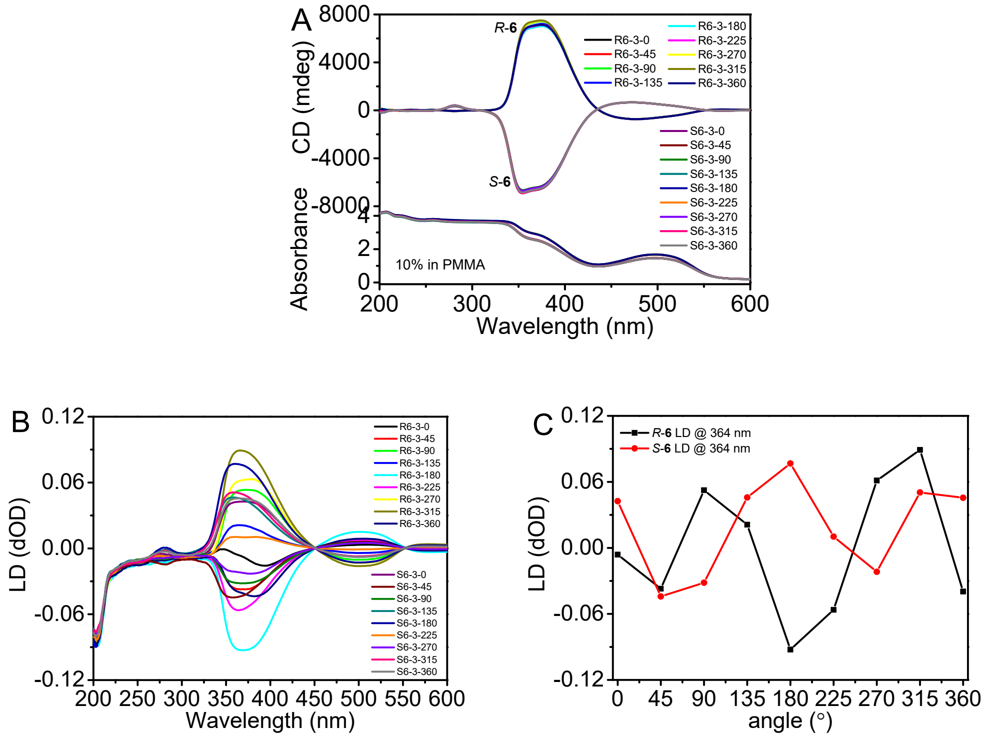

Fig. S31. Change in CD (A) and LD (B) spectra of *R*-**6** / *S*-**6** in PMMA films with different angles, and Intensity of LD vs angles (C). 10 weight% of **6** in PMMA.

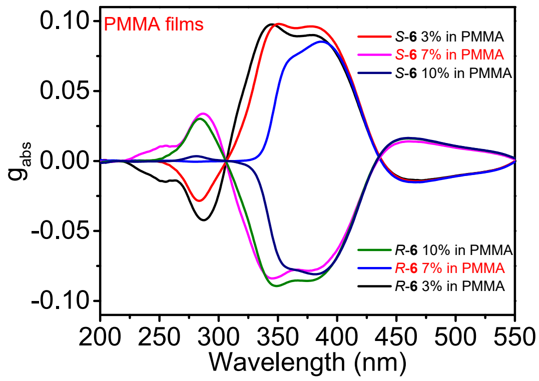

Fig. S32. Changing of g_{abs} with wavelength of *R*-**6** or *S*-**6** in PMMA films and weight% of **6** in PMMA.

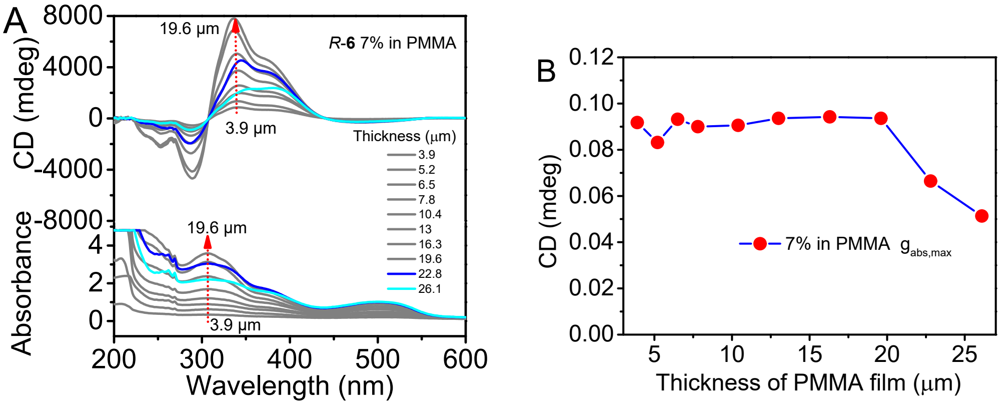

Fig. S33. Change in CD and adsorption spectra (A) and maximum of g_{abs} (B) of *R*-**6** in PMMA films with different thickness. 7 weight% of **6** in PMMA

The preparation method for PMMA films involves initially creating a toluene solution of PMMA at a concentration of 30 mg/mL. Subsequently, a corresponding amount of compound **6** or **7** is weighed based on the mass ratio between **6** or **7** and PMMA, and dispersed into the solution. The mixture is then homogenized through 30 minutes of ultrasonication to form a uniform emulsion. Using a pipette, an appropriate volume (from 60 μL to 400 μL) of the blended emulsion is evenly dispensed onto a 2×2 cm^2 quartz substrate. Upon evaporation of toluene, a uniformly thick PMMA film with an area of 4 cm^2 is obtained.

The approximate thickness of the PMMA film can be calculated based on the mass of PMMA (neglecting the added mass of compound **6** or **7**), density of PMMA (1.15 g/mL), and the film's area (4 cm^2). For instance, if 300 μL of the mixed solution are used for film preparation, the PMMA mass amounts to 9 mg, resulting in a calculated film thickness of approximately 19.6 μm.

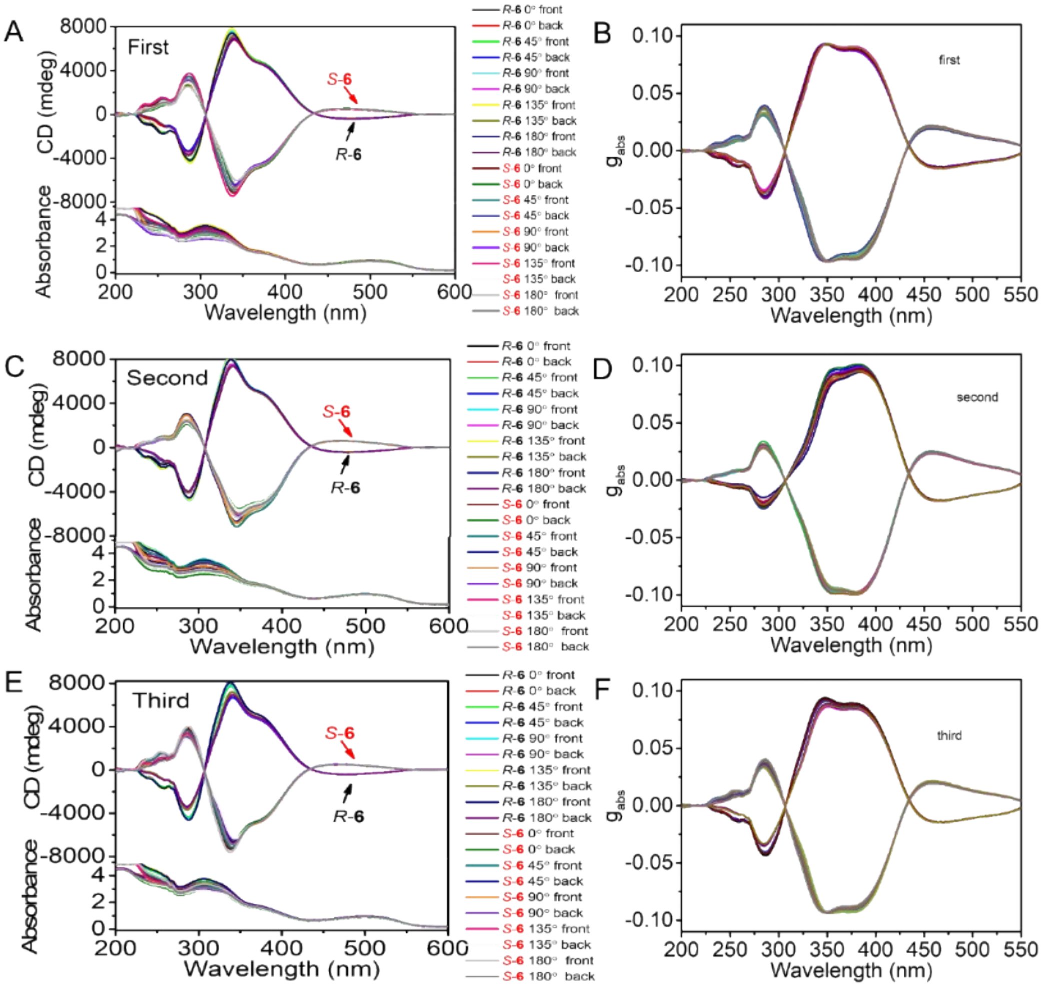

Fig. S34. Change in CD spectra (A, C, E) and change of g_{abs} with wavelength (B, D, F) of *R*-**6** / *S*-**6** in PMMA films with different angles. 7 weight% of **6** in PMMA, thickness 19.6 μm. Parallel experiments were carried out on three groups of newly prepared PMMA films.

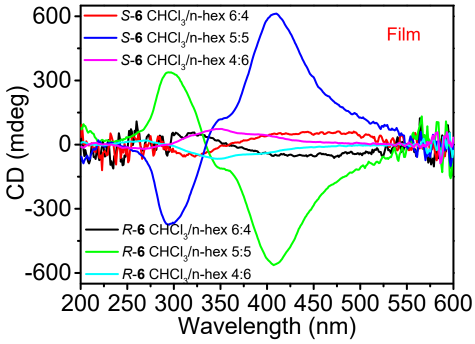

Fig. S35. CD spectra of drop-coating films of *R*-**6** and *S*-**6** in $CHCl_3$/n-hexane mixed solvent at different solvent ratios. [**6**] = 2.0 × 10^{-3} M.

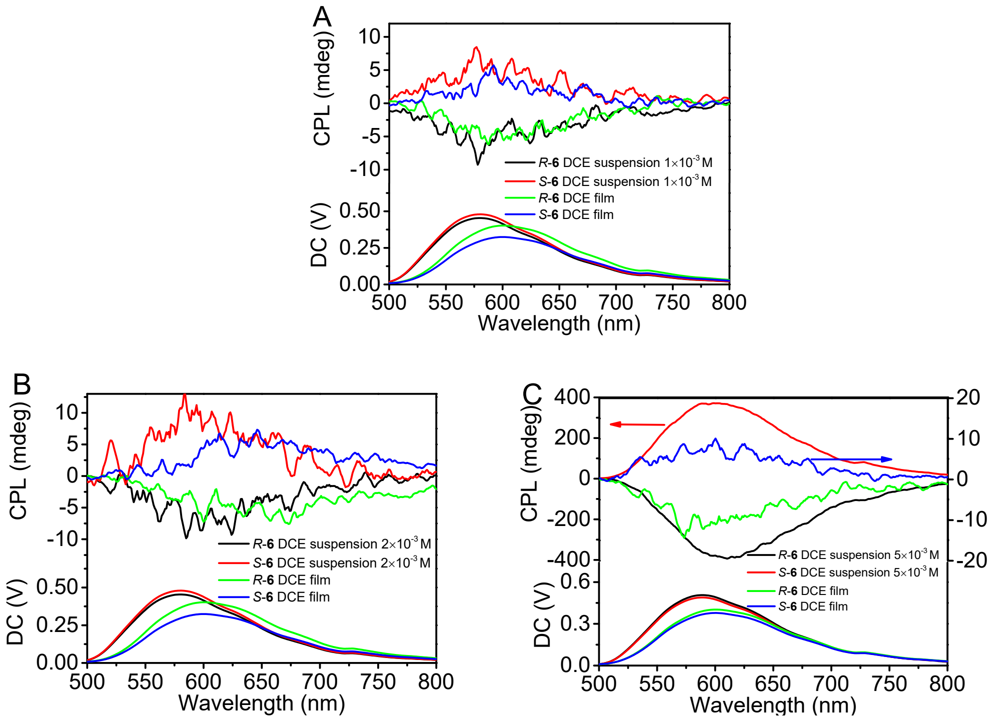

Fig. S36. CPL spectra of suspension of *R*-**6** and *S*-**6** in DCE at different concentration and in a drop-coating film (A) [**6**] = 1.0 × 10^{-3} M, (B) [**6**] = 2.0 × 10^{-3} M, (C) [**6**] = 5.0 × 10^{-3} M, (λ_{ex} = 350 nm).

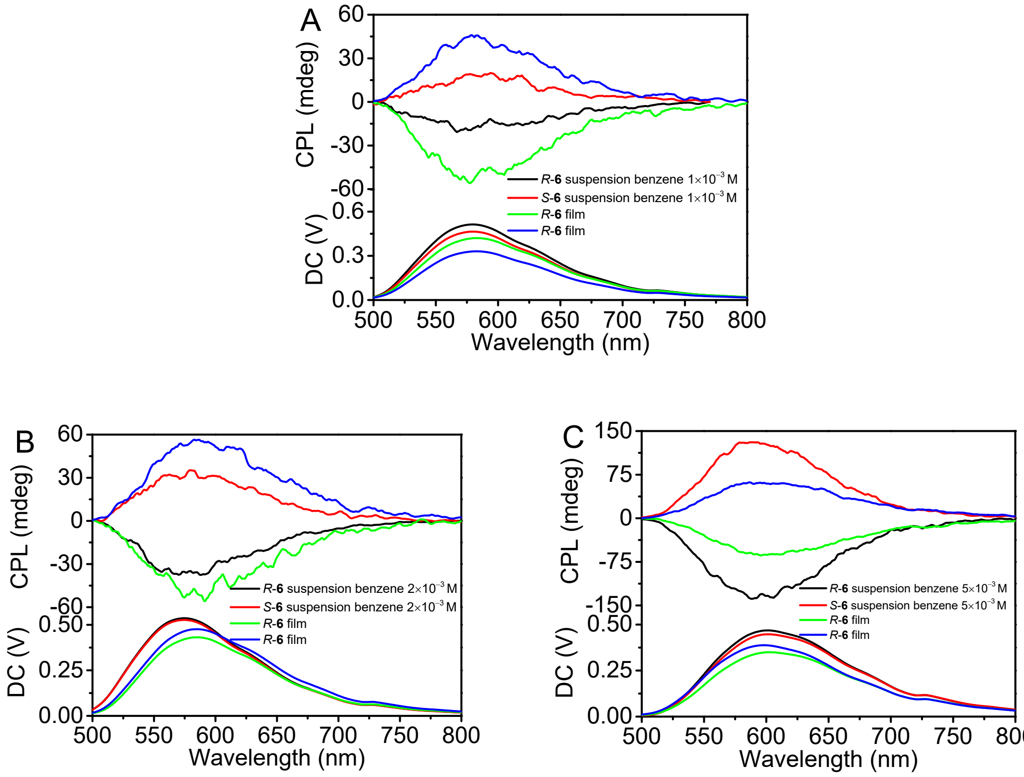

Fig. S37. CPL spectra of suspension of *R*-**6** and *S*-**6** in benzene and in a drop-coating film, (A) [**6**] = 1.0 × 10^{-3} M, (B) [**6**] = 2.0 × 10^{-3} M, (C) [**6**] = 5.0 × 10^{-3} M. λ_{ex} = 350 nm.

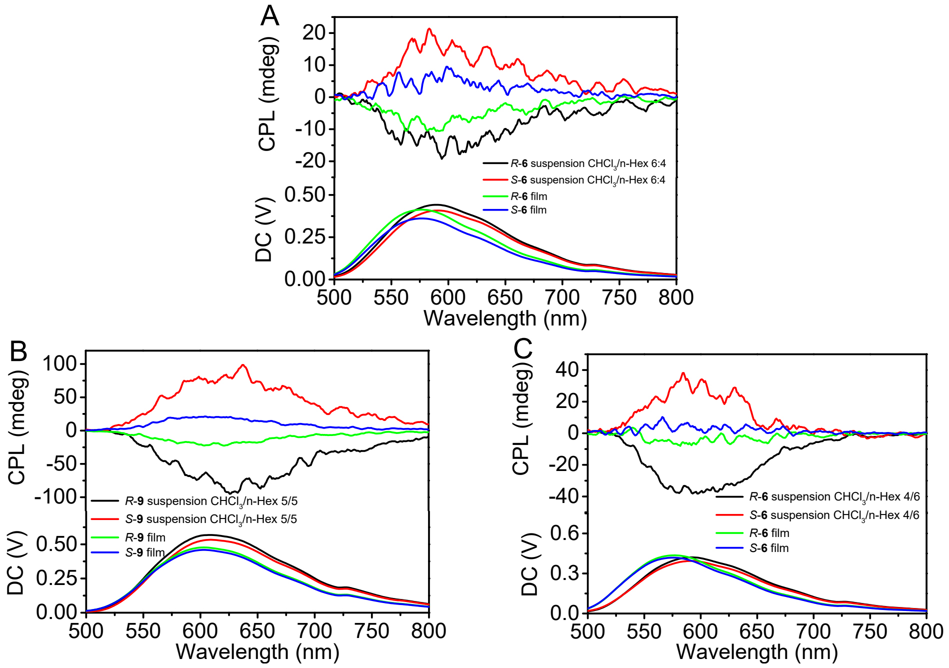

Fig. S38. CPL spectra of *R*-**6** and *S*-**6** in $CHCl_3$/n-hexane mixed solvent at different solvent ratios and in a drop-coating film (A) $CHCl_3$/n-hexane 6:4, (B) $CHCl_3$/n-hexane 5:5, (C) $CHCl_3$/n-hexane 4:6. λ_{ex} = 350 nm, [**6**] = 2.0 × 10^{-3} M.

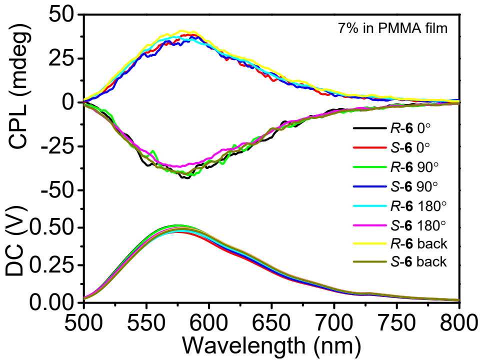

Fig. S39. Change in CPL spectra of *R*-**6** and *S*-**6** in PMMA film with different angles and faces. weight%, λ_{ex} = 350 nm.

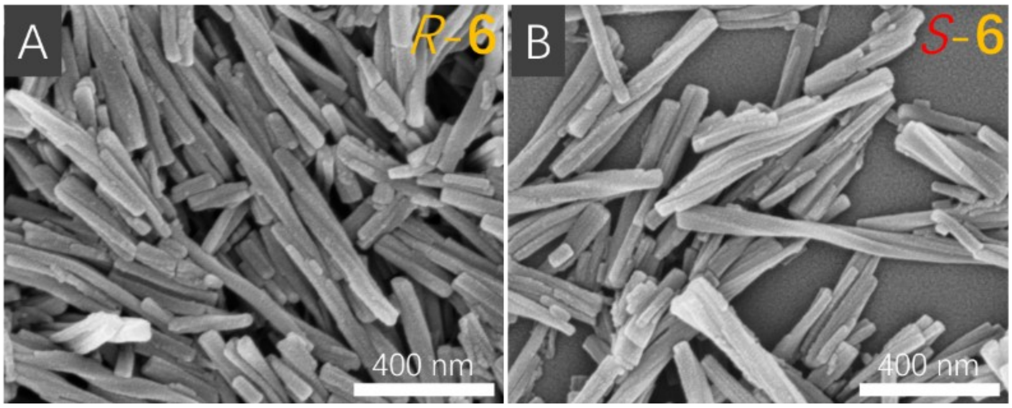

Fig. S40. SEM images of suspension of *R*-**6** and *S*-**6** in benzene. [**6**] = 5.0 × 10^{-4} M.

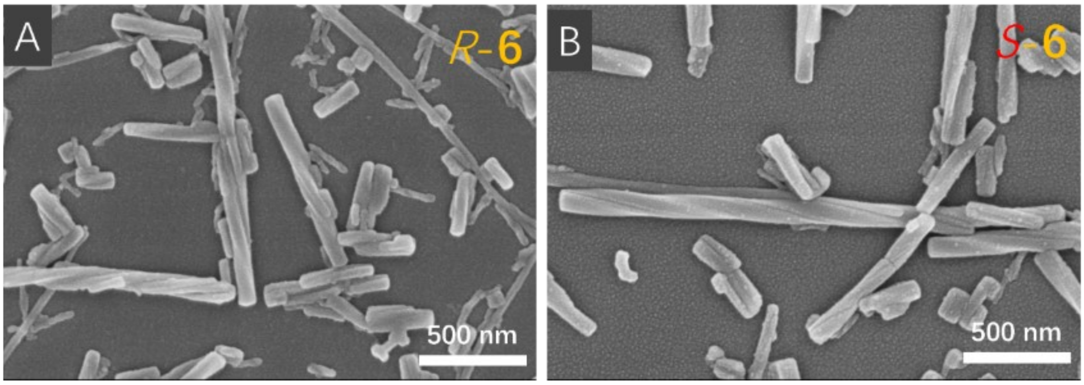

Fig. S41. SEM images of *R*-**6** and *S*-**6** in DCE suspension. [**6**] = 5.0 × 10^{-4} M.

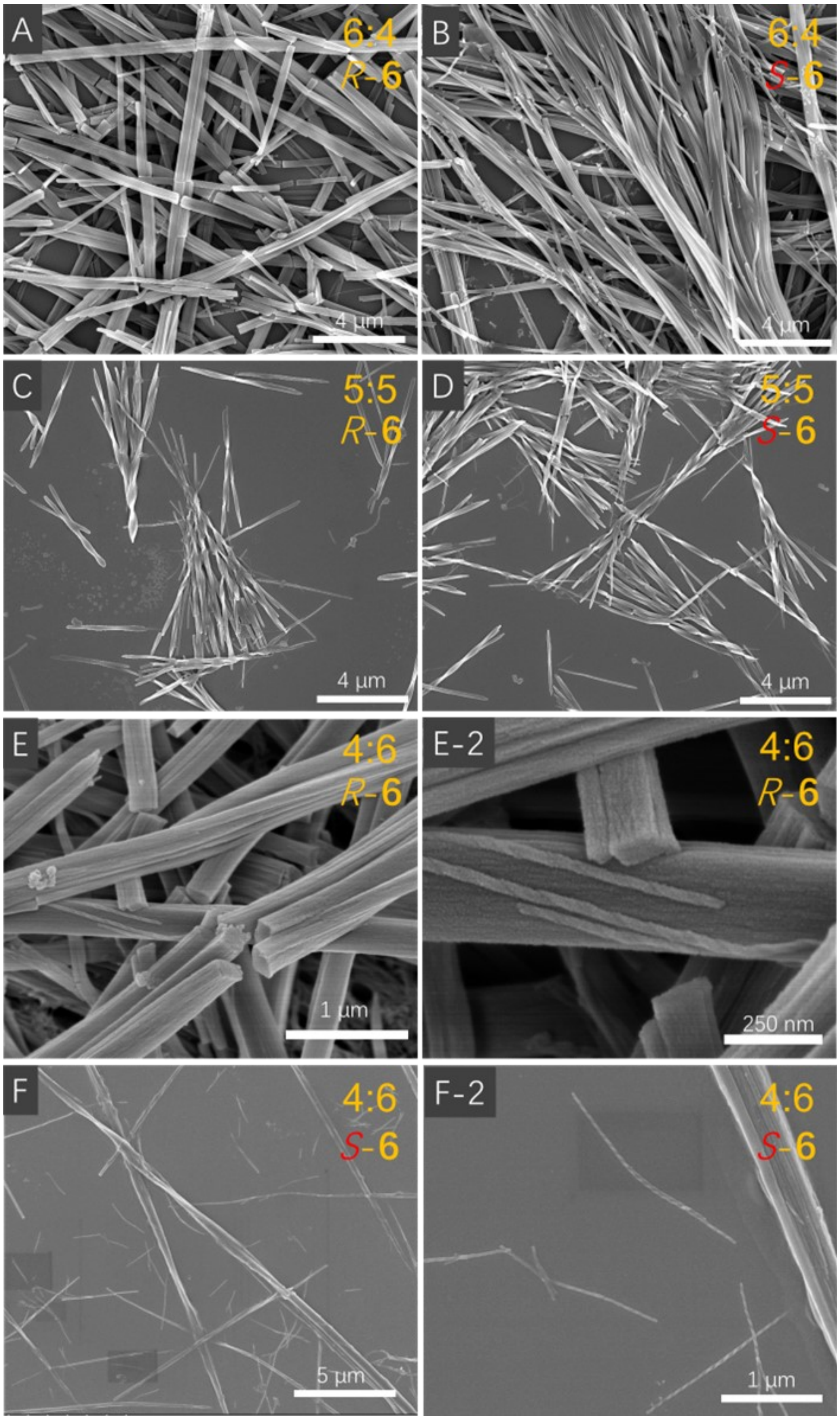

Fig. S42. SEM images of *R*-**6** or *S*-**6** in (A, B) $CHCl_3$/n-hexane 6:4, (C, D) $CHCl_3$/n-hexane 5:5, (E, F) $CHCl_3$/n-hexane 4:6. [**6**] = 2.0×10^{-3} M.

CD, CPL, SEM, and TEM of TPE bimacrocycle 7

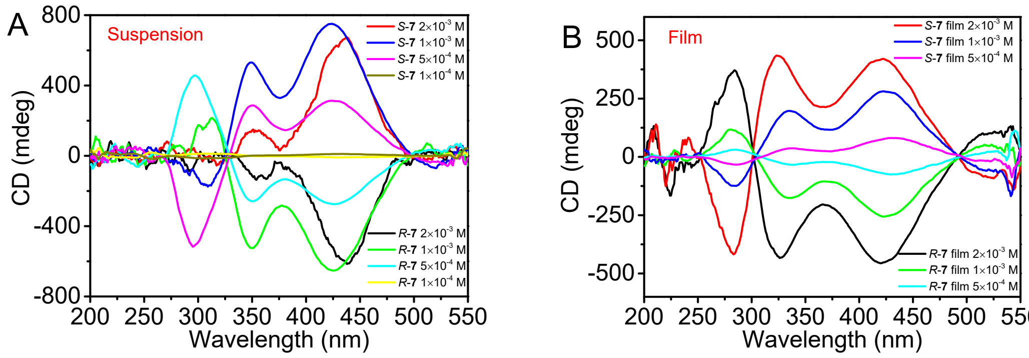

`

Fig. S43. CD spectra of suspension of *R*-**7** and *S*-**7** in DCE at different concentration (A) and in a drop-coating film (B).

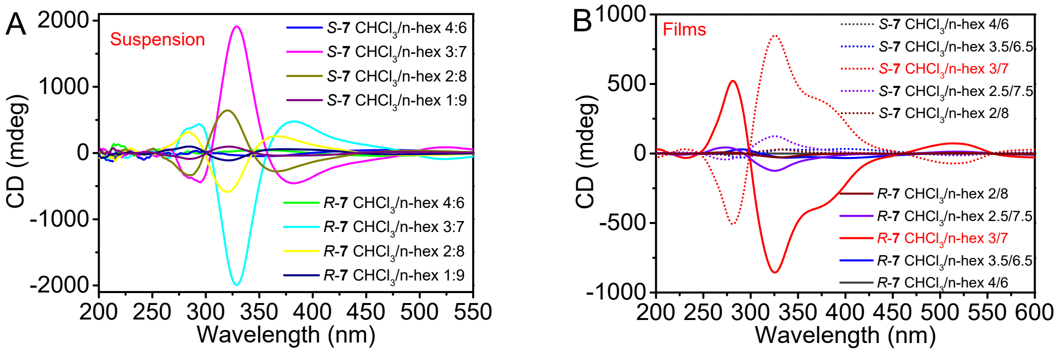

Fig. S44. CD spectra of *R*-**7** and *S*-**7** in $CHCl_3$/n-hexane mixed solvent at different solvent ratios (A) and in drop-coating films (B). [**7**] = 1.0×10^{-3} M.

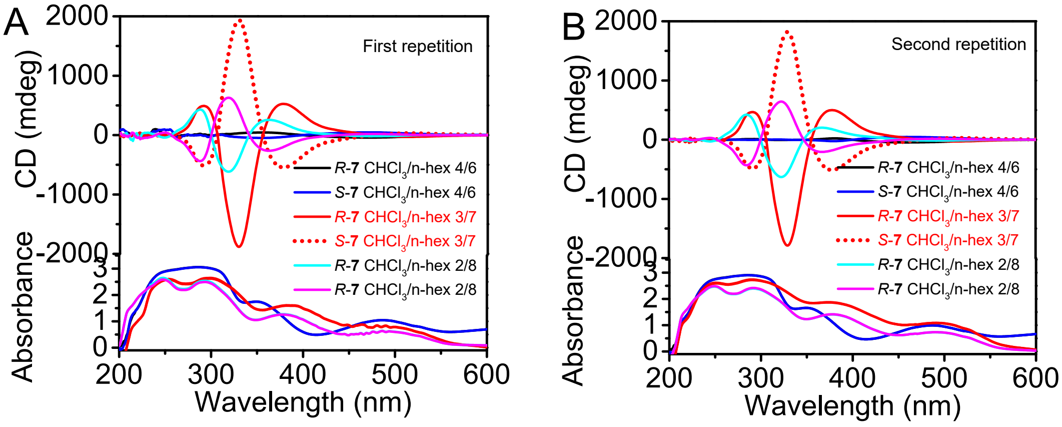

Fig. S45. CD and adsorption spectra of *R*-**7** and *S*-**7** in $CHCl_3$/n-hexane mixed solvent at different solvent ratios of the first (A) and second (B) parallel experiments.[**7**] = 1.0×10^{-3} M, spectra measured from two independent sets of samples.

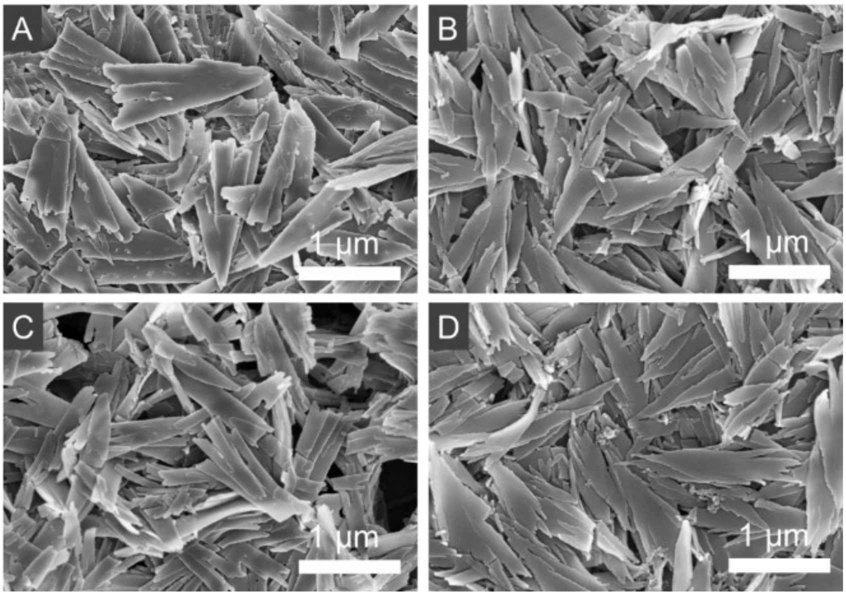

Fig. S46. SEM pictures of *R*-**7** or *S*-**7** in 70% $CHCl_3$/n-hexane mixed solvent of the first (A for *R*-**7**, B for *S*-**7**) and second (C for *R*-**7**, D for *S*-**7**) parallel experiments.[**7**] = 1.0 × 10^{-3} M, SEM measured from two independent sets of samples.

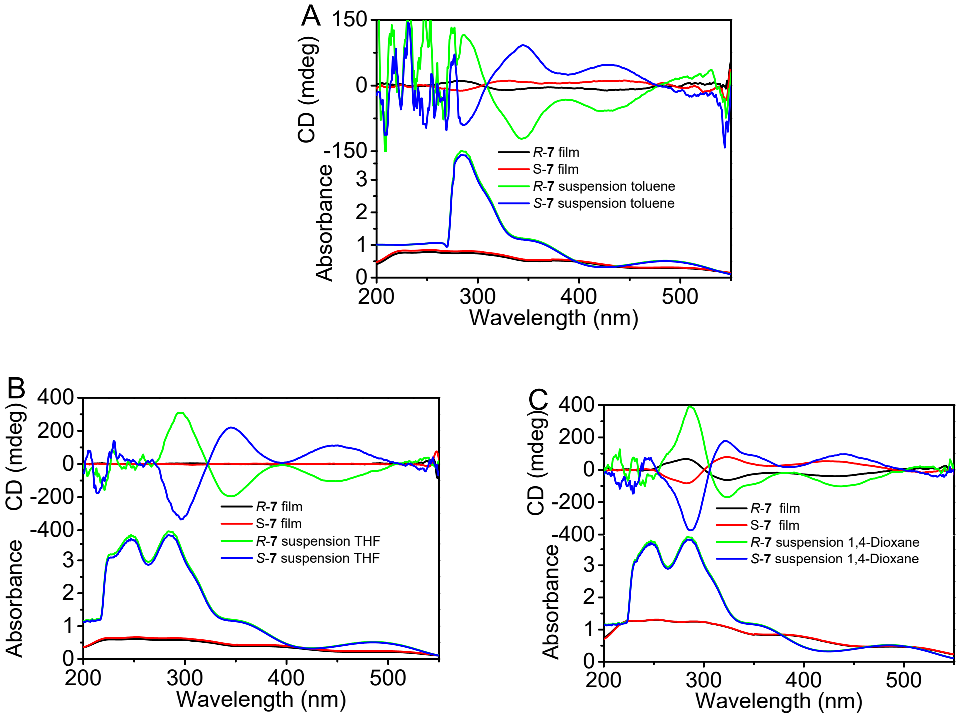

Fig. S47. CD and absorption spectra of suspension of *R*-**7** and *S*-**7** in different solvents and in a drop-coating film. (A) toluene, (B) THF, (C) 1,4-dioxane. [**7**] = 5.0 × 10^{-4} M.

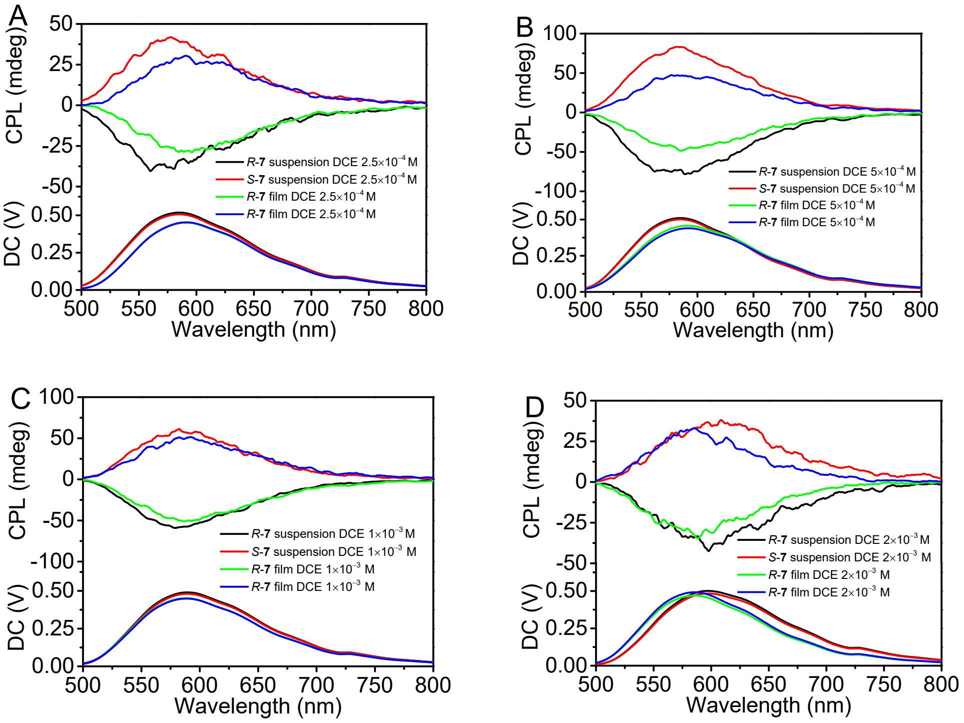

Fig. S48. CPL spectra of suspension of *R*-**7** and *S*-**7** in DCE at different concentration and in a drop-coating film. (A) [**7**] = 2.5 × 10^{-4} M, (B) [**7**] = 5.0 × 10^{-4} M, (C) [**7**] = 1.0 × 10^{-3} M, (D) [**7**] = 2.0 × 10^{-3} M. λ_{ex} = 350 nm.

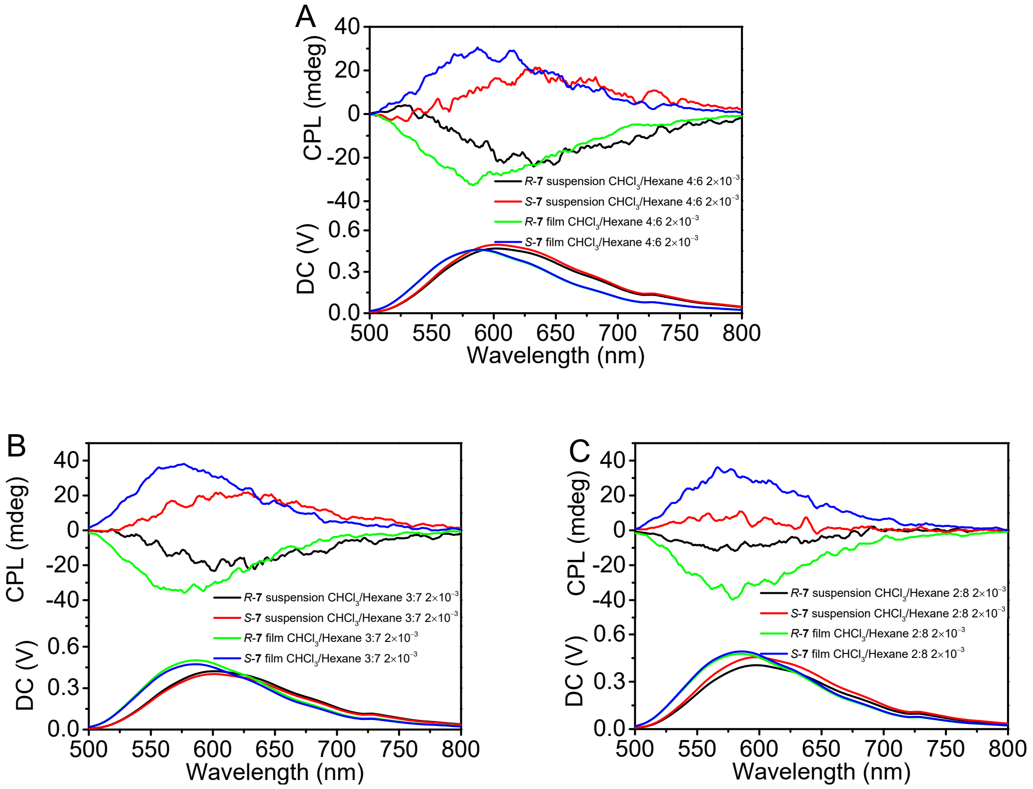

Fig. S49. CPL spectra of *R*-**7** and *S*-**7** in $CHCl_3$/n-hexane mixed solvent at different solvent ratios and in a drop-coating film. (A) $CHCl_3$/n-hexane 4:6, (B) $CHCl_3$/n-hexane 3:7, (C) $CHCl_3$/n-hexane 2:8. λ_{ex} = 350 nm, [**7**] = 2.0 × 10^{-3} M.

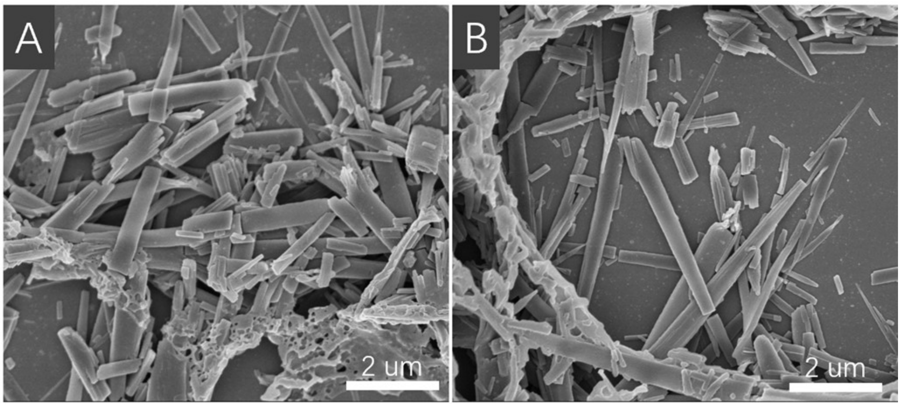

Fig. S50. SEM images of suspension of *R*-**7** (A) and *S*-**7** (B) in DCE. [**7**] = 5.0 × 10^{-4} M.

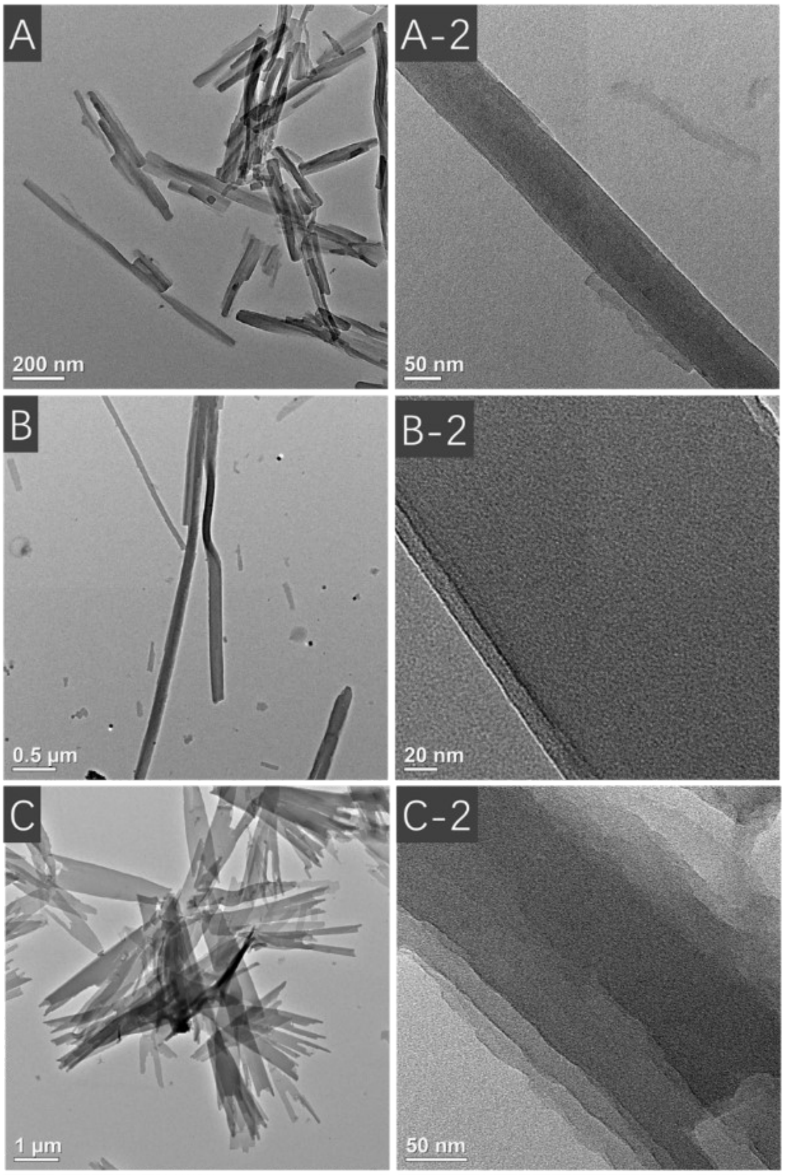

Fig. S51. HRTEM images of suspension of *R*-**6** in benzene (A), *R*-**7** in DCE (B), *R*-**7** in $CHCl_3$/n-hexane 3:7 (C). [**6**] = [**7**] =5.0 × 10^{-4} M.

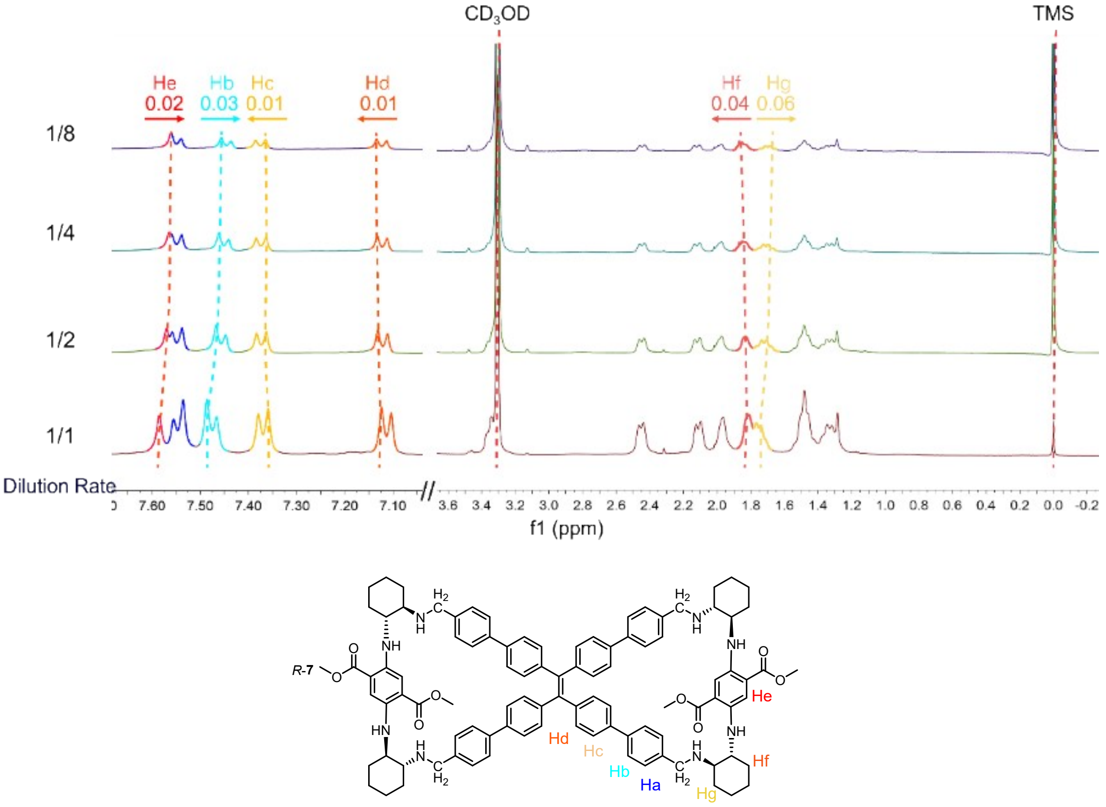

Fig. S52. Change in ^{1}H NMR spectra of *R*-**7**-4HCl in CD_3OD with concentration. [*R*-**7**-4HCl] = 2.5×10^{-3} M which was diluted to 1/2, 1/4, and 1/8. After the concentration was diluted by eight fold, the ^{1}H NMR spectra of *R*-**7**-4HCl in CD_3OD showed the largest shift for methylene protons (–0.06 ppm and +0.04 ppm), and large shift for the phenyl protons of benzyl groups (–0.03 ppm and +0.025 ppm) and for the protons of DATP (–0.02 ppm). This result demonstrated that the cyclohexyl ring of one molecule was included into the cavity of other molecule and a self-inclusion complex was formed in solution.

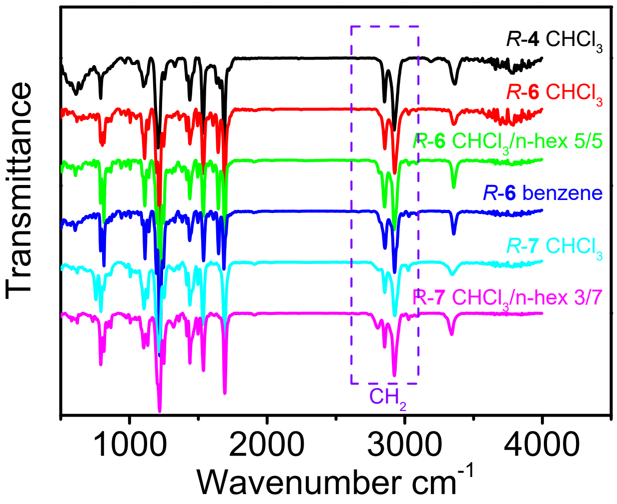

Fig. S53. FTIR spectra (E) of drop-cast films of *R*-**4**, *R*-**6** and *R*-**7** from different solvents.

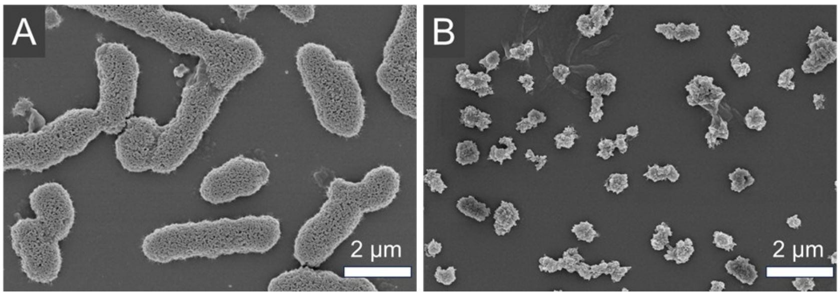

Fig. S54. SEM images of dropped-film of *R*-**6** (A) and *R*-**7** (B) from $CHCl_3$ solution. [**6**] = [**7**] = 5.0 × 10^{-4} M.

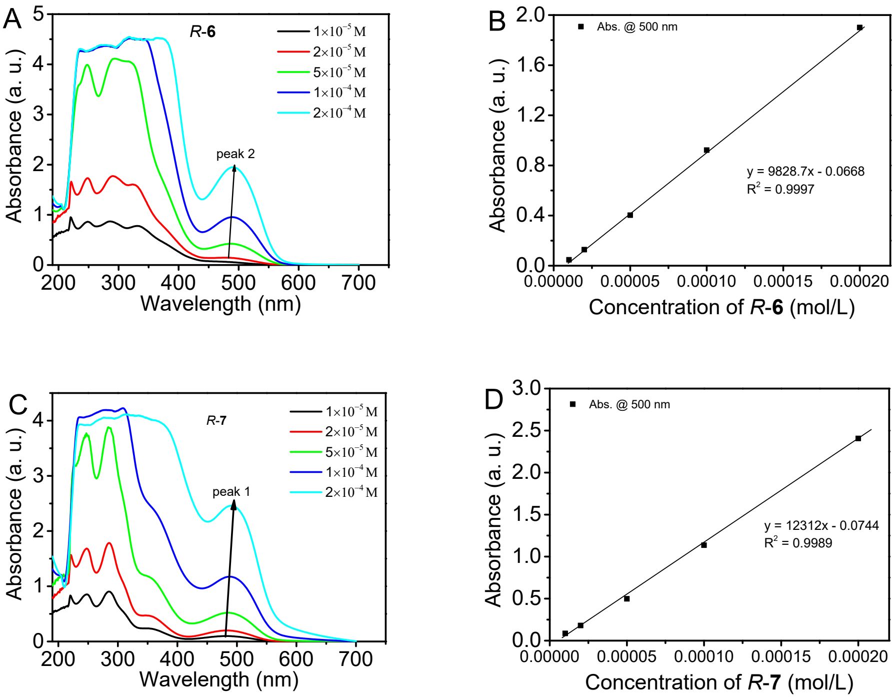

Fig. S55. Change in the UV-Vis spectra (A) and absorbance at 500 nm (B) of *R*-**6** in DCE with concentration, and change in the UV-Vis spectra (C) and absorbance at 500 nm (D) of *R*-**7** in DCE with concentration, the fitting curve is the relationship between absorption value and concentration of *R*-**6** or *R*-**7**.

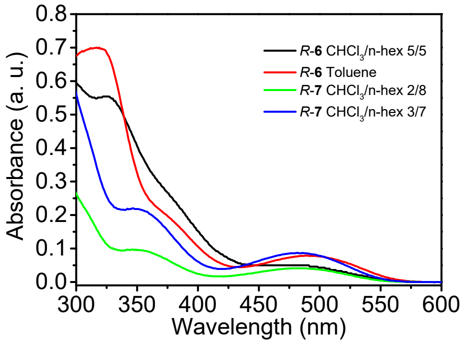

Fig. S56. The UV-Vis spectra were obtained from clearly saturated solutions of *R*-**6** and *R*-**7** in various solvents, which were attained through the filtration process of their respective self-assembled solutions.

Binding constant of self-inclusion of *R*-6 or *R*-7

After poor solvent is added, both *R*-**6** and *R*-**7** would aggregate and precipitate out of the solution, and have following equation:

$$nM \overset{K}{\rightarrow} (M)_n\downarrow$$

The binding constant, K, is therefore expressed as:

$$K = \frac{[(M)_n]}{[M]}$$

M represents the discrete macrocycle *R*-**6** or *R*-**7**, [*M*] denotes the concentration of the discrete macrocycle in solution, and $(M)_n$ represents aggregates of the macrocycle after self-assembly and self-inclusion occurred. Since $(M)_n$ is a solid, its concentration $[(M)_n]$ is a fixed value of 1, so the binding constant here is essentially the reciprocal of the monomer concentration:

$$K = \frac{1}{[M]}$$

After obtaining the self-assembled solution, the precipitate is filtered out to yield a clear solution, which can be considered to contain discrete compounds *R*-**6** and *R*-**7**. By further measuring the UV-Vis absorption spectra of different clear solutions, Fig. S56 is obtained. According to Beer's Law, at low concentrations, the absorption value of a compound exhibits a linear relationship with its concentration, as demonstrated in Fig. S55B and S55D. Consequently, based on the absorption value at 500 nm in Fig. S56, the corresponding concentration of compound *R*-**6** or *R*-**7** in the solution can be calculated. The reciprocal of these concentrations then corresponds to the binding constants, the calculation results are as follows:

Table S1. The calculation of binding constant of *R*-**6** or *R*-**7** in different solvents.

Entry	Absorption value at 500 nm (A, a.u.)	Fitting curve	Calculated concentration (C, mol/L)	Binding constant (M^{-1})
R-**6** in $CHCl_3$/n-hex 5/5	0.043	A = 9828.7C − 0.0668	1.1×10^{-5}	9.0×10^{4}
R-**6** in toluene	0.076	R^2 = 0.9997	1.5×10^{-5}	6.9×10^{4}
R-**7** in $CHCl_3$/n-hex 2/8	0.037	A = 12312C − 0.0744	9.1×10^{-6}	1.1×10^{5}
R-**7** in $CHCl_3$/n-hex 3/7	0.078	R^2 = 0.9989	1.2×10^{-5}	8.1×10^{4}

Chiral recognition and chiral analysis

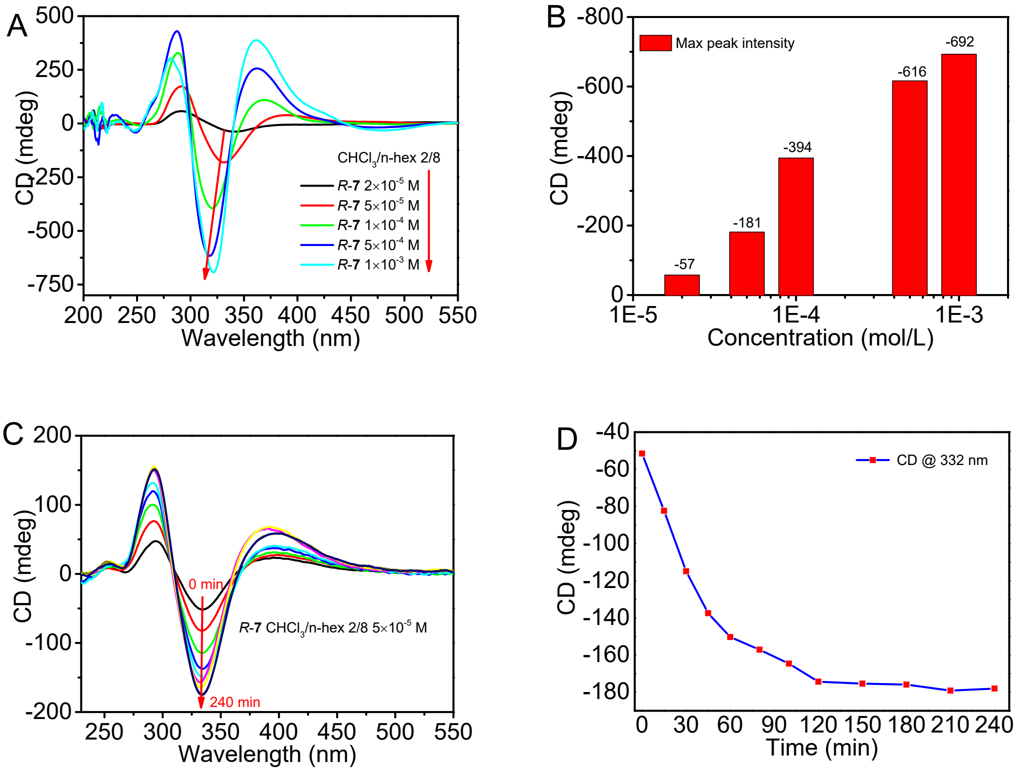

Fig. S57. Change in CD spectra of suspension of *R*-**7** in $CHCl_3$/n-hexane 2:8 with different concentration (A and B). Change in CD spectra of suspension of *R*-**7** with time that was left to stand after addition of 80% n-hexane into $CHCl_3$ (C) and change of CD intensity at 332 nm with time (D), [**7**] = 5.0×10^{-5} M.

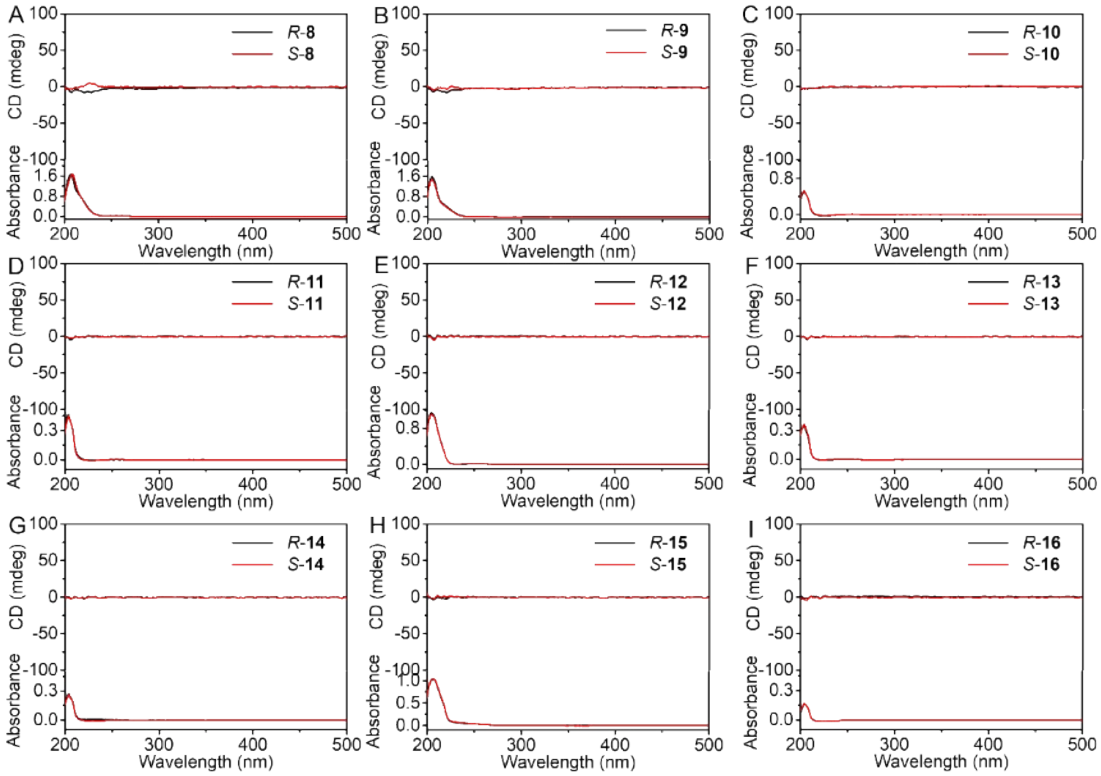

Fig. S58. CD spectra of two enantiomers of chiral molecules in $CHCl_3$/n-hexane 2:8. (A) 2-Chloromandelic acid **8**. (B) Mandelic acid **9**. (C) Camphorsulfonic acid **10**. (D) Malic acid **11**. (E) α-Methylbenzylamine **12**. (F) Cyclohexylethylamine **13**. (G) Cyclohexane-1,2-diamine **14**. (H) Phenylalanine **15**. (I) Menthol **16**. [**8**] = [**9**] = 2[**10**] = 2[**11**] = [**12**] = [**13**] = [**14**] = [**15**] = [**16**] = 1.0×10^{-4} M, the solvent for (H) contained 1% methanol, the solvent for (A)-(G) and (I) contained 1% ethanol.

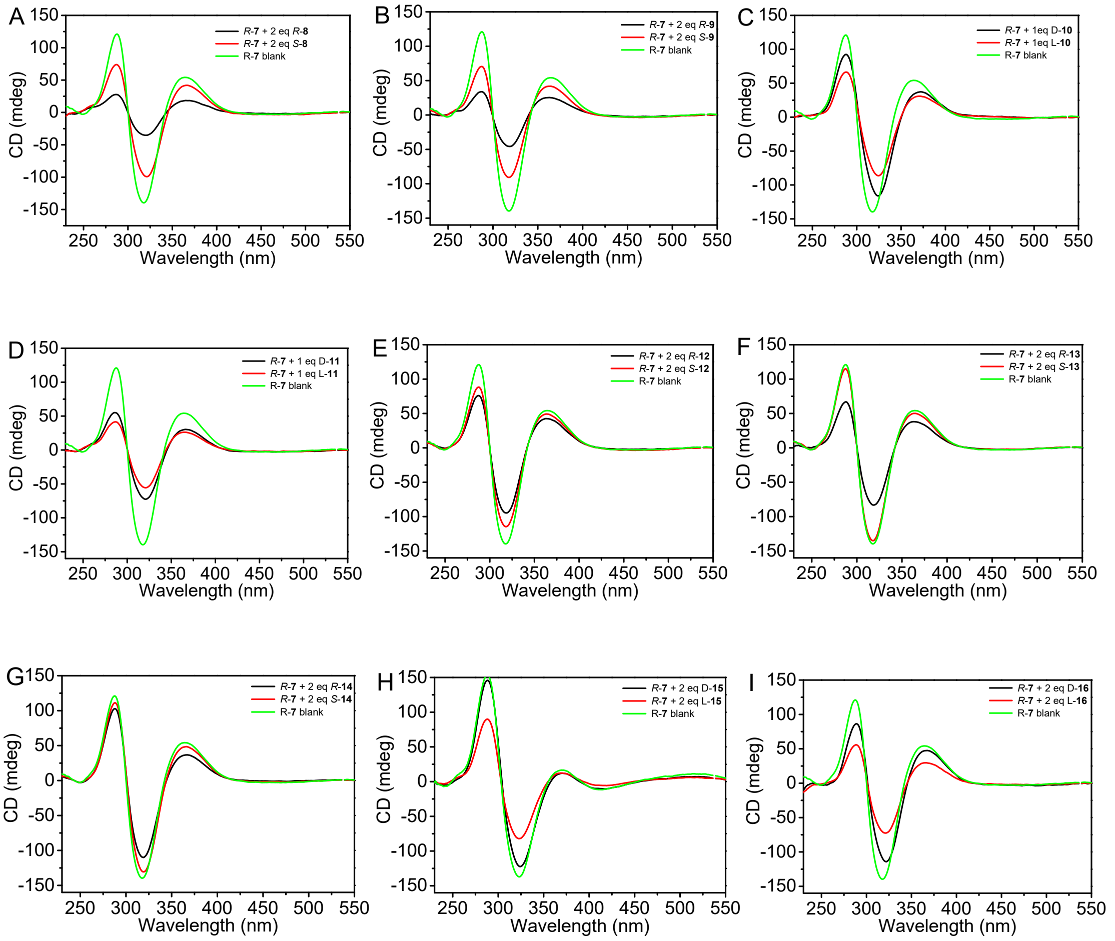

Fig. S59. Change in CD spectra of *R*-**7** with two enantiomers of a variety of chiral molecules in $CHCl_3$/n-hexane 2:8. (A) 2-Chloromandelic acid **8**/*R*-**7** 2:1. (B) Mandelic acid **9**/*R*-**7** 2:1. (C) Camphorsulfonic acid **10**/*R*-**7** 1:1. (D) Malic acid **11**/*R*-**7** 1:1. (E) α-Methylbenzylamine **12**/*R*-**7** 2:1. (F) Cyclohexylethylamine **13**/ *R*-**7** 2:1. (G) Cyclohexane-1,2-diamine **14**/*R*-**7** 2:1. (H) Phenylalanine **15**/*R*-**7** 2:1. (I) Menthol **16**/*R*-**7** 2:1. [*R*-**7**] = 5.0×10^{-5} M, the solvent for (H) contained 1% methanol, the solvent for (A)-(G) and (I) contained 1% ethanol.

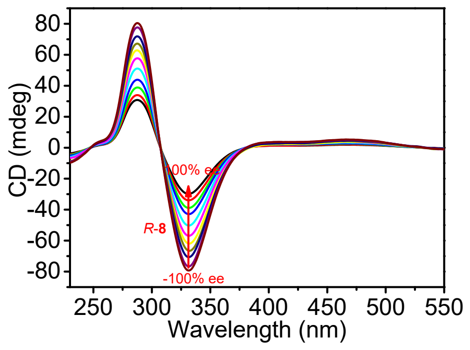

Fig. S60. Change in CD spectra of the suspension of *R*-**7** in $CHCl_3$/n-hexane/methanol 20:80:1 with ee% of *R*-**8**. [*R*-**7**] = 1/2[**8**] = 4.0×10^{-5} M.

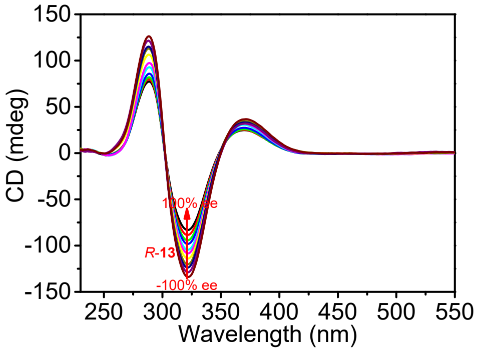

Fig. S61. Change in CD spectra of the suspension of *R*-**7** with ee% of *R*-**13** in $CHCl_3$/n-hexane/ethanol 20:80:1. [*R*-**7**] = 1/2[**13**] = 5.0 × 10^{-5} M.

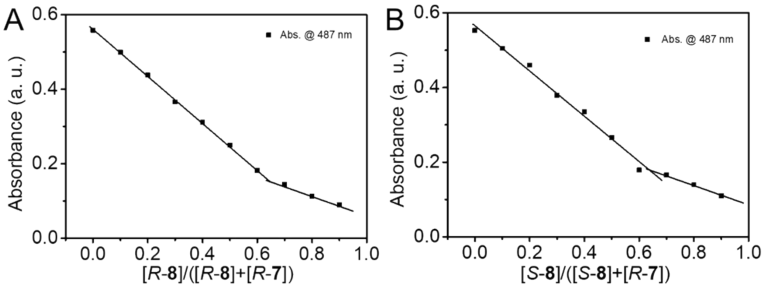

Fig. S62. Mole ratio plot for *R*-**7** and **8**, indicating 1:2 stoichiometry (A) *R*-**8**, (B) *S*-**8**. [*R*-**7**] + [**8**] = 5.0 × 10^{-5} M, $CHCl_3$/n-hexane 2:8.

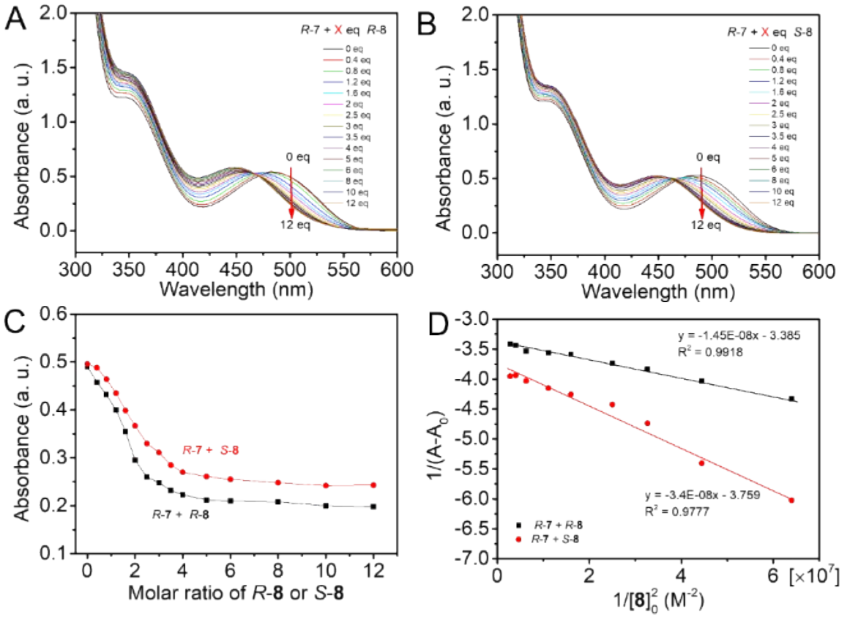

Fig. S63. Change of UV spectra of *R*-**7** with different molar ratio of (A) *R*-**8**, (B) *S*-**8** in solution, and (C) change of absorption values at 500 nm. The non-linear curve-fitting (UV titrations) for the host–guest complexation of *R*-**7** with different concentration of **8** (D), the binding constant was calculated by Benesi-Hildebrand equation for 1:2 association to be about 2.3 × 10^8 M^{-1} for *R*-**7**+*R*-**8**, and 1.1 × 10^8 M^{-1} for *R*-**7**+*S*-**8**. [*R*-**7**] = 5.0 × 10^{-5} M, $CHCl_3$/n-hexane 2:8.

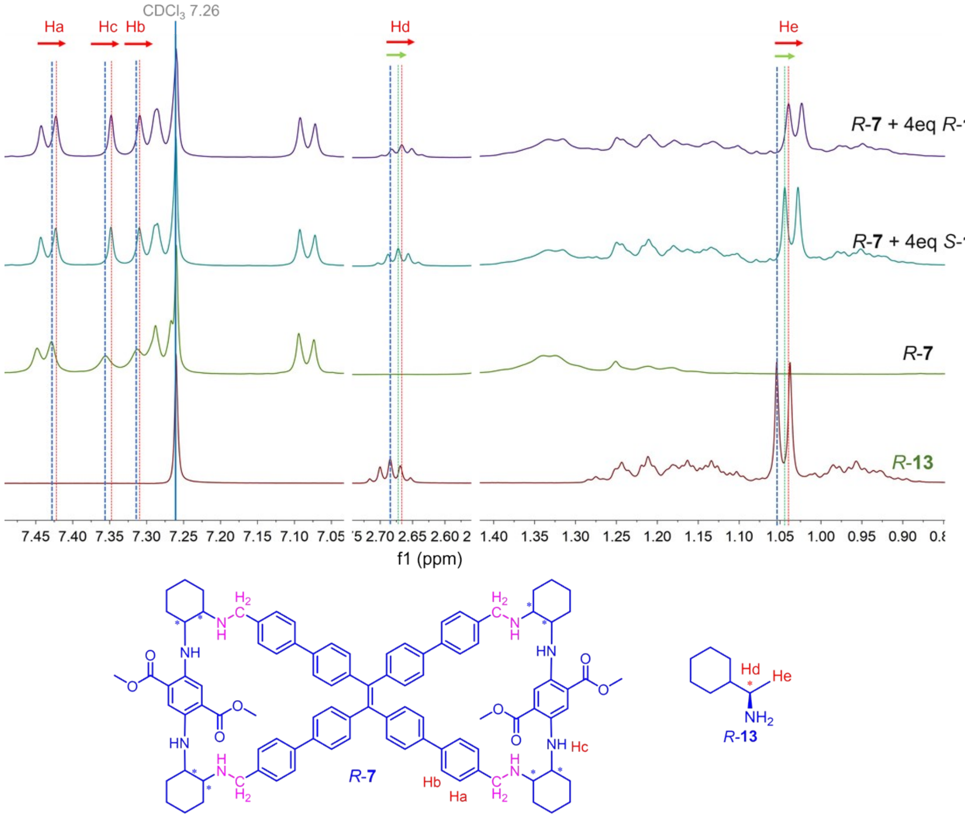

Fig. S64. ^{1}H NMR spectra of *R*-**7**, *R*-**13**, *R*-**7** + 4 eq *R*-**13** and *R*-**7** + 4 eq *S*-**13**. [*R*-**7**] = 1/4[**13**] = 2.0×10^{-3} M.

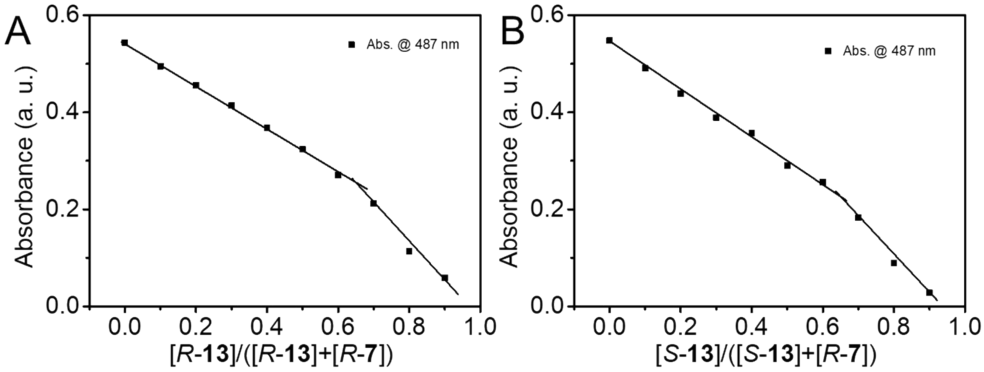

Fig. S65. Mole ratio plot for *R*-**7** and **13**, indicating 1:2 stoichiometry (A) *R*-**8**, (B) *S*-**8**. [*R*-**7**] + [**13**] = 5.0×10^{-5} M, $CHCl_3$/n-hexane 2:8.

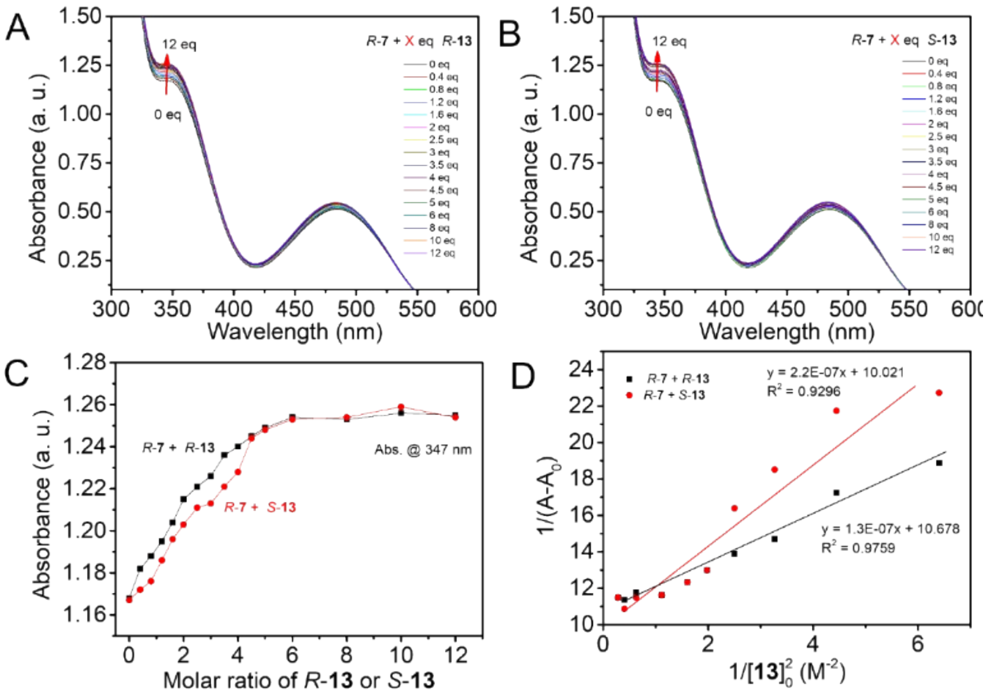

Fig. S66. Change of UV spectra of *R*-**7** with different molar ratio of (A) *R*-**13**, (B) *S*-**13** in solution, and (C) change of absorption values at 500 nm. The non-linear curve-fitting (UV titrations) for the host–guest complexation of *R*-**7** with different concentration of **13** (D), the binding constant was calculated to be about 8.2×10^7 M^{-1} for *R*-**7**+*R*-**13**, and 4.6×10^7 M^{-1} for *R*-**7**+*S*-**13**. [*R*-**7**] = 5.0×10^{-5} M, $CHCl_3$/n-hexane 2:8.

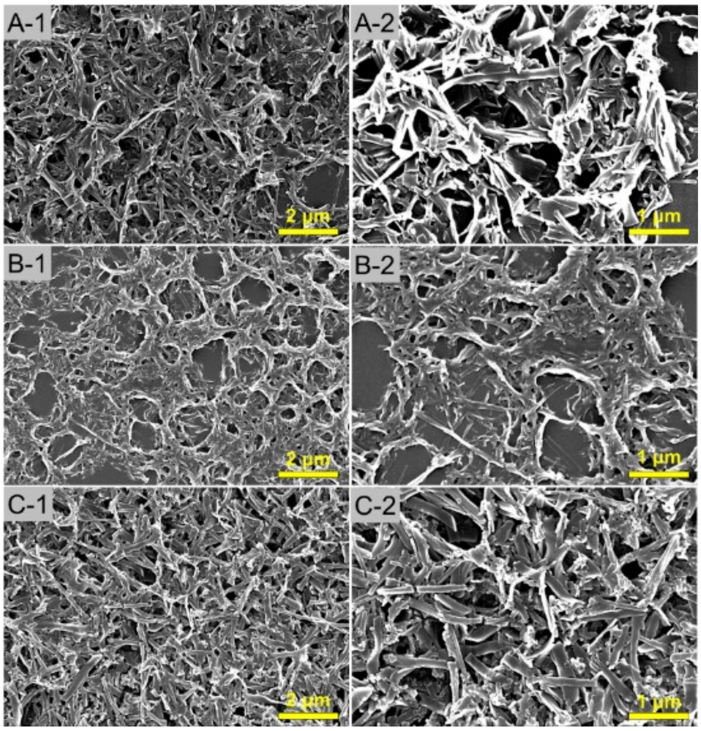

Fig. S67. SEM pictures of *R*-**7** (A, A-1), *R*-**7** + 2 eq *R*-**13** (B, B-1) and *R*-**7** + 2 eq *S*-**13** (C, C-1). [*R*-**7**] = 1/2[**13**] = 5.0×10^{-4} M, $CHCl_3$/n-hexane 2:8. *R*-**7** + 2 eq *R*-**13** has more fragmented assemblies, compared to *R*-**7** + 2 eq *S*-**13**.
